# Oxygen Evolution Reaction Catalysts for Acidic‐Media CO_2_ Electrolyzers

**DOI:** 10.1002/adma.72644

**Published:** 2026-02-27

**Authors:** Mingcheng Huang, Adnan Ozden

**Affiliations:** ^1^ Department of Mechanical and Nuclear Engineering Khalifa University Abu Dhabi UAE; ^2^ Electrochemical Systems Laboratory Khalifa University Abu Dhabi UAE; ^3^ Center for Catalysis and Separations (CeCaS) Khalifa University Abu Dhabi UAE

**Keywords:** acidic‐media CO_2_R, acidic‐media OER, electrochemical CO_2_ reduction, noble‐metal catalysts, non‐noble‐metal catalysts

## Abstract

Acidic‐media electrochemical CO_2_ reduction (CO_2_R) offers high single‐pass CO_2_ conversion (SPCE) and low purification cost, yet relies on scarce noble metals (e.g., iridium and ruthenium) for the anodic oxygen evolution reaction (OER). The limited abundance of these catalysts constrains large‐scale deployment. Drawing lessons from proton‐exchange membrane water electrolysis (PEMWE), this review integrates recent progress in acidic‐media OER catalysis with system‐level CO_2_R. It provides a cross‐scale perspective from mechanistic understanding and theoretical modeling to electrode architecture and operational optimization, highlighting how noble‐ and non‐noble‐metal catalysts can be engineered for enhanced activity, stability, and resource efficiency. The review further outlines diagnostic and computational approaches that connect atomic‐level descriptors with macroscopic durability. By bridging mechanistic insights and scalable system design, this work establishes a roadmap toward durable and economically viable acidic‐media CO_2_ electrolyzers.

## Graphical abstract

Acidic‐media CO_2_ electroreduction (CO_2_R) could decarbonize chemical production, despite relying on rare‐earth elements for anodic oxygen evolution reaction (OER). Transferring the learnings from mature sister technologies (water electrolysis) could accelerate technological development. This article, by focusing on acidic‐media OER for CO_2_R systems, overviews best practices for performance/stability assessment and progress in catalysts, catalysts characterization, and mechanistic approaches, providing a roadmap to practicality.

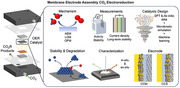

## 1 Introduction

The unprecedented growth in global population and economic activities creates an upward momentum in energy consumption. The rising energy demand is largely met by fossil‐fuel‐intensive processes. The resulting greenhouse gas emissions, such as carbon dioxide (CO_2_), pose a challenge for climate goals [1]. In 2023, global CO_2_ emissions reached a record high of 37.4 gigajoules (GJ) [2]. The trajectory of CO_2_ emissions reveals that global CO_2_ emissions will continue to increase, reaching 41–47.9 billion metric tons by 2050 [3]. The climate target goals set in the Paris agreement warrant carbon‐neutral and carbon‐negative technologies [4]. Technological advances contribute to the development of strategies, such as utilizing low carbon‐hydrogen‐ratio fuels in energy generation, increasing the efficiency of conversion processes, or using renewable energy sources. There are also emerging approaches, such as the utilization of CO_2_ emissions in the production of chemical feedstocks and fuels that are presently produced via fossil‐fuel‐intensive processes [5].

Electrochemical CO_2_ reduction (CO_2_R) is such a technology. CO_2_R, using CO_2_ emissions and renewable electricity, could produce a variety of fuels and chemical feedstocks, including carbon monoxide, methanol, ethylene, ethanol, and n‐propanol, among others [6]. These chemicals and fuels are ubiquitous to petrochemical industry, and they are presently manufactured from energy‐ and carbon‐intensive processes [6]. Thus, CO_2_R not only reduces CO_2_ emissions from conventional petrochemical processes but also facilitates the storage of intermittent renewable energy (e.g., solar and wind) in chemical bonds, stabilizing the global energy supply.

The efficiency, stability, and scalability of CO_2_R are influenced by the electrolyte and reactor configuration [7, 8]. Performing CO_2_R, using alkaline and neutral electrolytes, provides thermodynamic benefits by reducing the energy barrier of reaction intermediates [9]. The favorable reaction kinetics dominate CO_2_R over hydrogen evolution reaction (HER), enabling higher product selectivity. Besides, alkaline environment promotes multi‐carbon (C_2+_) products by favoring intermediate steps that are critical to carbon‐carbon (C─C) coupling [10, 11]. Steering CO_2_R toward C_2+_ products, such as ethylene, improves practicality by reducing energy intensity and production cost. However, alkaline and neutral electrolytes trigger the reaction between CO_2_ molecules and hydroxide (OH^–^) ions, causing CO_2_ loss to (bi)carbonate formation and impractical single‐pass CO_2_ conversion efficiencies (SPCEs). The negatively charged (bi)carbonate ions crossover to the anode through the anion exchange membrane (AEM). The (bi)carbonate ions are then converted back to CO_2_, mixing with oxygen produced from oxygen evolution reaction (OER) [12]. The recovery of CO_2_ from the anodic downstream involves energy‐intensive separation, deteriorating techno‐economics. The (bi)carbonate formation also deleteriously impacts the performance and stability of CO_2_R. The performance and stability decay primarily arises from (1) alkalinity loss due to the migration of OH^–^ ions from the microreaction environment and (2) salt precipitation in the CO_2_‐carrying channels of gas diffusion electrodes (GDEs) [13].

Acidic‐media CO_2_R systems could enable a SPCE of >85% by regenerating the (bi)carbonate ions formed at the cathodic micro‐reaction environment, and the regenerated CO_2_ then participates in CO_2_R [9, 14, 15]. This phenomenon limits the crossover of (bi)carbonate ions to the anode and ensuing CO_2_ loss. Acidic‐media CO_2_R systems typically utilize proton exchange membranes (PEMs). However, the protons generated via the OER crossover to the cathode through the PEM, creating an intense proton flux to the cathode [16]. A portion of the protons reaching the cathode reacts with OH^–^ ions to produce water, and the remaining portion reacts with (bi)carbonate ions (formed via the reaction between OH^–^ ions and CO_2_) to regenerate CO_2_ [17, 18]. The continuous flux of protons from the anode to the cathode creates new challenges, despite inhibiting the CO_2_ loss associated with neutral‐ and alkaline‐media systems [5, 19, 20]. For instance, the continuous proton migration from the anode could cause fluctuations in the acidity of the anode, depending on the proton utilization rate at the cathode [21]. This could degrade the anodic performance through the dissolution of the OER catalyst [22, 23]. Further, the ensuing cross‐over of the positively charged metallic species to the cathode through the PEM could degrade the performance of CO_2_R [24, 25]. Meanwhile, the continuous flux of protons to the cathode could also form a relatively less favorable reaction environment for CO_2_R and more favorable environment for HER [26, 27].

The acidic‐media CO_2_R research is presently centered around effective strategies that could steer CO_2_R toward C_2+_ products. Today's laboratory‐scale research focuses on designing active and selective catalysts [28, 29, 30, 31], leveraging cation effects [32, 33], modulating micro‐reaction environment [13], and modifying catalyst surface [34]. The catalyst design strategies hinge on reducing the energy barrier of key reaction intermediates, modulating the distribution and stabilization of reaction intermediates, and tuning the reaction pathways. The cation effects involve — by optimizing the cation identity and composition — the modulation of reaction pathways, reaction kinetics, and intermediate coverage and stabilization. The micro‐reaction environment tuning and catalyst surface modification strategies involve promoting local alkalinity, optimizing intermediate adsorption and cation effects, constraining proton transport to the active sites, or controlling cation availability at active sites. In essence, all these strategies target performance and stability enhancements.

Recent progress in acidic‐media CO_2_R systems is encouraging. Benchmark acidic‐media CO_2_R systems deliver a C_2+_ Faradaic efficiency (FE) of >80%, a C_2+_ full‐cell energy efficiency of >28%, a current density of 150 mA cm^−2^, and stable operation of 150 h [33]. However, achieving breakeven energy intensities (i.e., 80 GJ ton^−1^ for ethylene) warrants further improvements [35], including a FE of >95%, a productivity of >400 mA cm^−2^, a full‐cell energy efficiency of >60%, and a near‐unity SPCE [5]. Additionally, wide‐scale technology deployment warrants transferring these metrics to scalable systems and enduring acidic‐media CO_2_R for year‐long operation. Coupling these metrics will warrant effective system design approaches. This, in turn, requires developing scalable and stable acidic‐media CO_2_R systems by taking a full‐system approach.

In the context of system design, the anode catalyst's specifications are critical and could dominantly affect the performance, stability, scalability, and sustainability of acidic‐media CO_2_R systems. Ideally, an acidic‐media anodic catalyst should combine productivity, energy efficiency, operational stability, and scalability. Noble‐metal catalysts, such as iridium (Ir) and ruthenium (Ru), exhibit favorable activity and stability under acidic conditions [36, 37]. Today's benchmark CO_2_ electrolyzers rely heavily on iridium oxide (IrO_2_) catalysts to realize high‐rate and efficient OER at the anode [15, 16, 30, 33, 38, 39]. However, noble‐metal catalysts pose challenges that jeopardize the feasibility and scalability of acidic‐media CO_2_R. Ir and Ru, as the benchmark acidic‐media OER catalysts, are among the rarest and most expensive elements. The supply of these metals shows inhomogeneous geographic distribution, imposing supply instability, trade restrictions, and price fluctuations. These factors cause high price volatility: Ir price of $4, 500–$5500 per ounce (2025) and Ru price of $550–$900 per ounce (2025) [40, 41]. The heavy reliance on these metals for the manufacture of anode electrodes drives up the cost of electrolyzers, rendering capital expenditures prohibitive and imposing barriers ahead of industrial implementation. Overall, despite acidic‐media CO_2_R systems present an efficient solution for (bi)carbonate formation and associated separation costs, their stability and scalability require advancements in anodic reactions.

The OER plays critical roles in various electrochemical systems with technology‐readiness levels (TRLs) greater than that of CO_2_R systems. Water electrolyzers represent a good example to such electrochemical systems. The intrinsic system similarities between water and CO_2_ electrolyzers offer significant opportunities for transferring the know‐how and best practices to emerging CO_2_R systems. PEM water electrolyzers (PEMWEs) and acidic‐media CO_2_R systems resemble each other in a way that the OER is performed using acidic electrolytes typically over Ir‐based catalysts [36, 42, 43]. Decades of water electrolysis research has identified catalyst design and system integration strategies for activity, cost, and scalability enhancements. These advances have paved the way toward commercialization [44]. Today, PEMWEs are globally recognized as an efficient and scalable technology for hydrogen production [36, 43, 45]. Despite the certain discrepancies between the reaction environments, rapid transfer of the learnings and best practices from PEMWEs to CO_2_R systems could still accelerate performance, stability, and cost improvements. This would remove a critical barrier ahead of practical CO_2_R. A comprehensive review of transferable practices, including catalyst design and synthesis, performance assessment and characterization, and degradation mitigation strategies, could serve as a guide and contribute to technological advancement.

This review explores the recent advances and best practices for acidic‐media OER catalysts. The article focuses on the knowledge and practices that are transferable to acidic‐media CO_2_R systems, providing guidelines for the design of active, efficient, stable, and scalable OER catalysts for acidic‐media CO_2_R systems. The article begins with a technology overview of acidic‐media CO_2_R systems, highlighting their potential for carbon‐efficient CO_2_R while discussing challenges. The article analyzes the need for high‐performance, stable, and scalable OER catalysts for practical acidic‐media CO_2_R systems. The review then discusses recent advances in acidic‐media OER catalysts: the best practices for understanding reaction mechanisms and evaluating electrochemical performance; recent advances in the design of noble‐ and non‐noble OER catalysts; ex and in situ catalyst characterization strategies; and experimental, computational, and characterization approaches for mechanistic insights. The article ends with detailed prospects on strategies that could enhance the practicality, stability, scalability, and sustainability of acidic‐media OER (hence acidic‐media CO_2_R) through design, development, and system integration of innovative catalysts from experimental and theoretical standpoints. The review — by addressing these critical research themes — provides a viable roadmap for advancing CO_2_R technology, encouraging a full‐system approach for practical and scalable acidic‐media CO_2_R. The focus of this review is to bridge the advances in the field of acidic‐media OER with acidic‐media CO_2_R systems, emphasizing their unique interfacial and operational coupling — a perspective that has been rarely addressed in prior reviews.

## 2 CO_2_R Technology and Acidic‐Media CO_2_R

**FIGURE 1:**
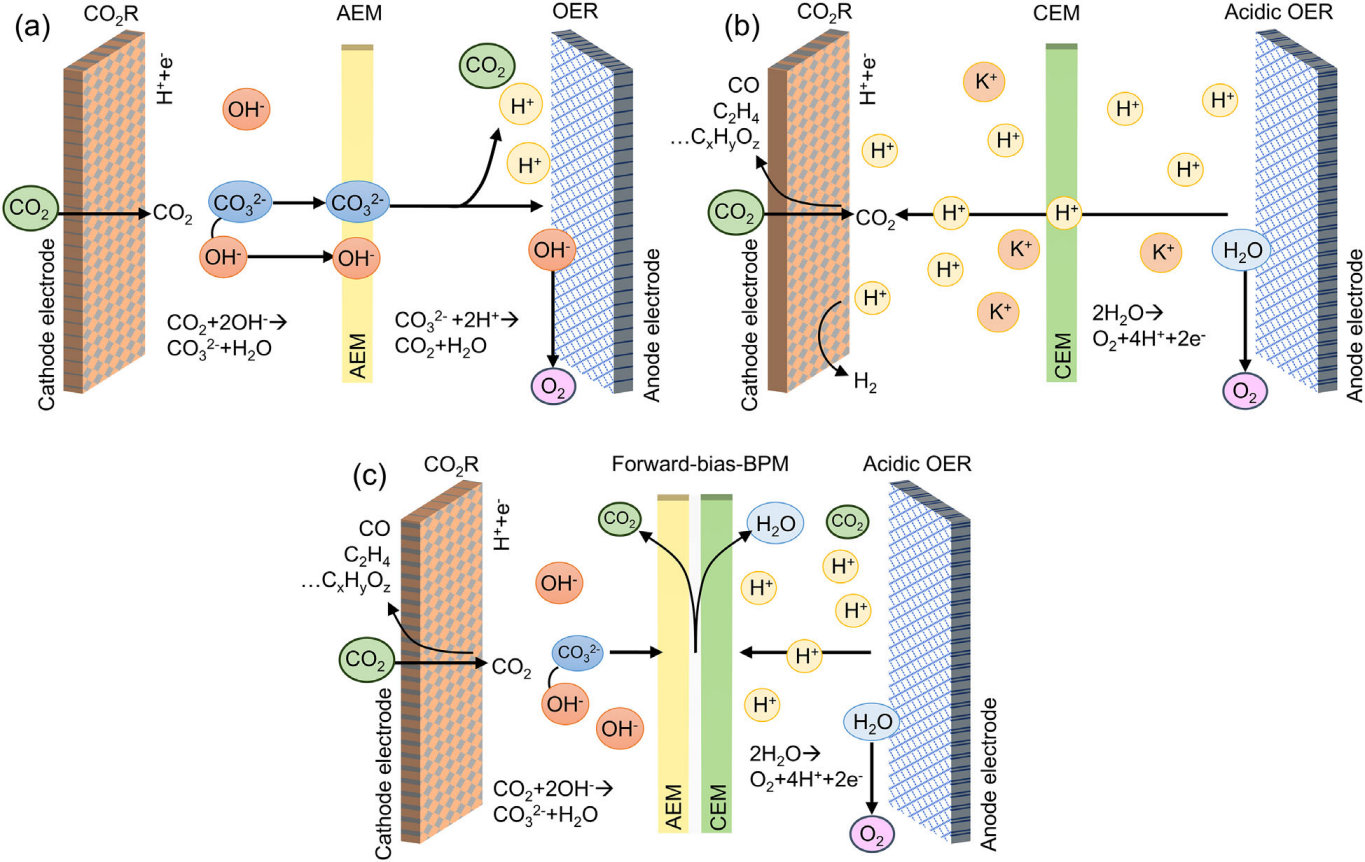
Schematic illustration of MEA and flow of ions and protons for the neutral/alkaline/acidic media MEA cells. (a) AEM‐based MEA electrolyzer, (b) CEM‐based MEA electrolyzer, (c) Forward‐bias BPM‐based MEA electrolyzer. (a–c) Reproduced with permission [9]. Copyright 2025, Elsevier.

CO_2_R can be performed in H‐type cells, flow cells, and membrane electrode assembly (MEA) electrolyzers using alkaline, neutral, and acidic electrolytes. Alkaline and neutral electrolytes enable a thermodynamically favorable environment for CO_2_R. From the system standpoint, performing OER with alkaline electrolytes offers performance and cost benefits. Scalable nickel (Ni)‐based catalysts can catalyze OER in alkaline electrolytes at high reaction rates and efficiencies [46, 47]. However, such CO_2_R systems encounter severe “(bi)carbonate formation”, due to the rapid reaction between reactant CO_2_ and locally generated OH^–^ ions (Figure 1a) [9, 48]. This unwanted reaction limits the SPCE toward C_2+_ products to impractical levels. While neutral‐media CO_2_R systems show less CO_2_ loss to (bi)carbonate formation (Figure 1a), they fail to achieve practical SPCEs. In these systems, the reactant CO_2_ turns into (bi)carbonate ions and crosses‐over the anode via the AEM, where the (bi)carbonate ions are converted back to CO_2_. The CO_2_ mixes with oxygen being produced via OER, introducing energy‐intensive anodic separation process (Figure 1a). The loss of reactant CO_2_ and energy‐intensive separation processes impose practicality challenges to neutral‐media CO_2_R systems.

Acidic‐media CO_2_R addresses the (bi)carbonate formation, ensuring effective CO_2_ utilization [5, 16, 27, 38, 49]. Acidic conditions minimize the reaction between CO_2_ molecules and OH^–^ ions, regenerating CO_2_ from (bi)carbonate species [15]. Acidic‐media CO_2_R is typically performed in flow cells or zero‐gap MEA electrolyzers. The acidic‐media flow cells are based on a gas‐feed configuration where CO_2_ is supplied to the catalytic sites through a GDE. The catalyst faces the cathode compartment where the acidic electrolyte is circulated. Similarly, the anode compartment is supplied with an acidic electrolyte [5, 19, 50, 51, 52, 53]. The acidic flow cells can be designed with two or three compartments. The three‐compartment system contains an intermediate chamber where the reference electrode is placed near the cathode. The two‐compartment system, also known as slim flow cell, contains cathode and anode compartments, and the cell voltage captures the anodic and cathodic potentials. MEA electrolyzers, also known as zero‐gap electrolyzers, are the systems where the cathode is free of electrolyte, and the electrodes and membrane are stacked as an assembly (Figure 1) [54]. MEA electrolyzers offer a suitable platform for C_2+_ products, owing to their high efficiency, compact design, and compatibility with industrial‐scale applications. Similar to the flow‐cell configuration, the anode performs acidic‐media OER, and thus it is supplied with pure water or acidic electrolyte. The proximity between the components minimizes ohmic losses, enabling greater energy efficiencies [55]. Additionally, the absence of electrolyte at the cathode avoids electrolyte‐related flooding or contamination, albeit introducing difficulties in modulating the micro‐reaction environment.

In both flow cell and MEA configurations, the anode and cathode chambers can be separated via a PEM (Figure 1b). In PEM‐based systems, the anode is fed with pure water or acidic electrolytes. In PEM‐based configurations, the protons generated via the OER crossover to the cathode, and the continuous proton flux balances the locally generated OH^–^ ions. The (bi)carbonate ions formed via the reaction between OH^–^ ions and CO_2_ molecules are also converted back to CO_2_ through their reaction with protons. MEA electrolyzers — performing acidic‐media CO_2_R — can also be equipped with bipolar membranes (BPMs) in forward‐bias (f‐BPM) configuration (Figure 1c) [39]. In f‐BPM systems, the cathode faces the anion exchange layer (AEL) of the BPM, whereas the anode faces the cation exchange layer (CEL). The protons generated by the OER and the OH^–^ ions generated by the CO_2_R crossover to the interface of CEL and AEL. The f‐BPM CO_2_R systems — by regenerating CO_2_ at the AEL/CEL interface in lieu of the catalyst surface — enable higher local alkalinity compared to PEM‐based CO_2_R systems, forming a more favorable platform for CO_2_R (Figure 1c). However, f‐BPM systems encounter voltage and stability losses, due to the accumulation of CO_2_ and water at the AEL/CEL interface [56]. Additionally, BPMs, due to the presence of two layers (AEL and CEL) and interfaces, typically encounter higher ohmic losses than PEM‐based systems, suffering lower energy efficiencies at practical reaction rates (i.e., >200 mA cm^−2^). However, in PEM‐based systems, steering CO_2_R toward C_2+_ products remains a critical challenge.

Catalyzing OER at high rates over low‐cost and scalable catalysts with low overpotentials in pure water or acidic electrolytes represents an ideal scenario for acidic‐media CO_2_R. However, the highly oxidative anode environment necessitates acid‐resistant OER catalysts. Noble‐metal catalysts, such as Ir and Ru, could combine high OER activity and stability. However, the prohibitive cost of these materials imposes scalability limitations [56]. Practical acidic‐media CO_2_R systems require robust, active, and low‐cost catalysts that can catalyze OER at practical rates and efficiencies throughout prolonged electrolysis.

Overall, conducting CO_2_R in an acidic electrolyte enables regeneration of CO_2_ via the reaction of protons with carbonate (CO_3_^2^^−^) and bicarbonate (HCO_3_^−^) ions, thereby preventing CO_2_ loss and enabling high SPCEs. The minimized reactant loss improves system techno‐economics by reducing the energy input associated with electrolyte regeneration or anodic gas separation. Maintaining these attributes over prolonged CO_2_R requires taking a full‐system approach, such as pairing the CO_2_R catalysts with active, stable, and scalable OER catalysts.

## 3 Significance of OER for Acidic‐Media CO_2_R

CO_2_R systems utilize anodic reactions to maintain a charge balance between the anode and cathode. In full systems, CO_2_R is typically paired with OER — a reaction that oxidizes water and produces oxygen. While oxygen offers no significant commercial value, it can be safely vented to the atmosphere without affecting CO_2_R, ensuring operational convenience, clean product streams, and continuous operation [57]. Thus, the utilization of OER enables operational convenience for large‐scale applications, rendering it favorable in the pursuit of CO_2_R. Because OER produces no salable product, it is critical to ensure that the process feasibility is not negatively impacted by its sluggish or unstable kinetics. Accordingly, an OER catalyst should be designed and optimized to minimize its contribution to capital and operational costs. Unfortunately, the voltage breakdown of a benchmark CO_2_ electrolyzer indicates that approximately 19% of the full‐cell potential is taken up by the anodic potential to drive OER at 150 mA cm^−2^ [58]. Herein, the anode electrode consists of a noble‐metal IrO_x_ catalyst supported on titanium (Ti) with a mass loading of 1 mg cm^−2^. Despite the use of noble‐metal catalysts with high mass loadings, the CO_2_R system suffers prohibitive potentials. However, the practicality‐enabling performance, stability, and cost requirements mandate active, efficient, low‐cost, and scalable OER catalysts.

Although half‐cell measurements provide intrinsic kinetic descriptors for OER catalysts under controlled electrochemical conditions, full‐cell operation represents the practical realization of these catalysts within membrane‐based devices. In acidic‐media CO_2_ electrolyzers, the anode operates within a coupled electrochemical environment, where oxygen evolution, proton transport through the membrane, ion crossover, and catalyst layer (CL) architecture collectively govern the apparent anode behavior and overall cell voltage. Consequently, full‐cell measurements should not be interpreted as a direct validation of half‐cell overpotentials, but rather as an assessment of how effectively intrinsic OER activity is realized under realistic operating conditions. This distinction underpins the discussion below, where full‐cell performance is analyzed from a system‐level perspective.

In recent years, several studies have explored the integration of acidic‐media OER catalysts into CO_2_ electrolyzers for system level investigations under low‐pH conditions. Most reported systems employ IrO_2_ or RuO_2_‐based catalysts coated on Ti meshes or porous transport layers (PTLs), analogous to PEMWEs, to maintain anodic stability. For example, IrO_x_/Ti anodes have been used in MEA‐type acidic‐media CO_2_R systems, achieving continuous operation for several hours but exhibiting voltage decay rates of 10–15 mV h^−^^1^ due to Ir dissolution and Ti corrosion [50]. Meanwhile, a spinel‐type Co_2_MnO_4_ catalyst demonstrated stable acidic‐media OER at pH 1, achieving continuous operation for > 1500 h at 200 mA cm^−^^2^, yet still suffering from partial Co dissolution and lower intrinsic activity than Ir‐based oxides [59]. Although still limited in durability, such studies underscore the potential of alternative materials beyond Ir and Ru. These emerging demonstrations establish an important technical bridge between conventional PEMWEs and acidic‐media CO_2_ electrolyzers, highlighting how the specifications of OER catalysts govern full‐cell efficiency, durability, and carbon utilization.

Despite their significance for CO_2_R systems, there is currently a dearth of performance and stability investigations into OER catalysts specifically designed for coupling with CO_2_R. The experimental testing and performance/stability demonstrations in acidic‐media CO_2_R systems rely solely on Ir‐based catalysts and Ti‐based substrates. Despite IrO_x_ and Ti substrates constituting one of the costliest catalyst/substrate pairs, acidic‐media CO_2_R systems still fail to maintain voltage stability for prolonged periods. Specifically, f‐BPM catholyte‐free MEA exhibits 8 h of CO_2_R using IrO_x_/Ti as the anode and pure water as the anolyte [17]. The system exhibits a high voltage decay rate of 10–15 mV h^−1^ at a modest current density of 100 mA cm^−2^. The dissolution of the anode and ensuing cross‐over and accumulation of metallic ions at the cathodic active sites cause rapid performance and voltage loss. It is worth noting that such degradation behavior observed under device‐relevant current densities (≥100 mA cm^−^^2^) cannot be directly inferred from conventional half‐cell stability tests conducted at low current densities (e.g., 10 mA cm^−^^2^), where mass‐transport limitations, gas‐evolution‐induced stresses, and cross‐electrode coupling effects are largely suppressed.

Beyond contributing to the anodic overpotential, the OER electrode plays a critical role in governing the durability of PEM‐based acidic‐media CO_2_R configurations through coupled degradation processes. Under strongly acidic and oxidizing conditions, IrO_x_ catalysts and Ti‐based supports are thermodynamically and kinetically prone to gradual dissolution, releasing Ir‐ and Ti‐containing cations into the anolyte. Driven by electro‐osmotic drag, concentration gradients, and the electric field across the membrane, these dissolved metal species can migrate toward the cathode and accumulate within the CO_2_R CL or at the membrane–cathode interface. The presence of foreign metal cations near the CO_2_R electrode could perturb the local ionic strength, electrical double‐layer structure, and effective proton activity, thereby altering CO_2_R kinetics and product selectivity while promoting parasitic reactions, such as HER. In addition, metal‐ion deposition or ion‐induced blockage of active sites and ionomer domains increases interfacial resistance, leading to a progressive rise in cell voltage during extended operation. This degradation cascade—spanning anode dissolution, ion crossover, and cathode contamination—highlights that the OER electrode in acidic‐media CO_2_ electrolyzers is not a passive counter electrode, but an active determinant of full‐cell stability and performance.

Despite being critical, literature presently lacks full‐system approaches. That is, the implications of OER catalysts on the performance, efficiency, stability, and scalability of acidic‐media CO_2_R systems have been somewhat overlooked. The scarcity of such comprehensive investigations originates from multiple constraints: (i) the harsh acidic environment accelerates catalyst dissolution and substrate corrosion, making long‐term testing challenging; (ii) noble‐metal Ir/Ru catalysts, though stable, are costly and scarce, limiting their exploration beyond lab‐scale; (iii) cross‐electrode coupling, including cation crossover and metal‐ion migration, complicates data interpretation and necessitates sophisticated membrane–electrode integration. Consequently, many CO_2_R studies focus on cathodic performance while adopting conventional PEMWE‐type OER electrodes without comprehensive mechanistic assessment. Expanding this research frontier requires systematic durability benchmarking and development of low‐loading noble catalysts or robust non‐noble catalysts. The CO_2_R catalysts have undergone extensive research. CO_2_R, with rapid progress, can now be selectively steered toward various CO_2_R products at practical productivities [32, 33, 38]. Despite slight discrepancies, the overpotentials of benchmark catalysts with similar product distributions show similarity. Similar overpotentials obtained with many catalysts suggest that there might be a small gap to be bridged (via catalyst design strategies) from the cathodic voltage loss standpoint. In contrast, the variations in the physical characteristics and activities of anode catalysts cause large fluctuations in current densities, overpotentials, energy efficiencies, and operational stabilities [58]. Additionally, from the scalability standpoint, the anode electrode consisting of an OER catalyst (i.e., Ir) and an acid‐resistant substrate (i.e., Ti) makes the largest contribution to the capital cost [60, 61, 62, 63]. These performance and cost implications underscore the significance of acidic‐media OER for the practicality of CO_2_R systems.

Table 1 compares a PEMWE and an acidic‐media CO_2_ electrolyzer, combining both quantitative performance metrics—such as overpotential, durability, and Ir loading—and a schematic overview of the distinct anodic and interfacial environments in the two systems. The representative data were compiled from recent benchmark studies and are indicative rather than strictly comparable, as variations in cell configuration, electrolyte composition, and testing duration inevitably affect absolute values. In a PEMWE, the anode performs the OER and the cathode performs the HER in a clean proton environment with minimal species crossover. In contrast, acidic‐media CO_2_ electrolyzers exhibit additional complexities arising from CO_2_ diffusion, local acidity fluctuations at the anodic interface induced by spontaneous proton‐involved reactions, and cation or metal‐ion migration across the membrane, which can modify both cathode selectivity and anode stability. Moreover, the coexistence of steep ionic gradients and mixed‐gas environments can accelerate Ir or Ru dissolution and Ti substrate corrosion, highlighting the need for OER catalysts that maintain high activity and structural integrity under low‐pH and high‐potential conditions while resisting contamination and electrode coupling effects.

**TABLE 1:** Comparison of a PEMWE and an acidic‐media CO_2_ electrolyzer.

Parameter	PEMWE (Typical)	Acidic‐media CO_2_ Electrolyzer (Typical)	Notes/Key differences
Anodic reaction	2H_2_O → O_2_ + 4H^+^ + 4e^−^	Same OER reaction	Shared anodic mechanism
Cathodic reaction	4H^+^ + 4e^−^ → 2H_2_	CO_2_ + nH^+^ + ne^−^ → fuels/chemicals	Different products
Main goal	H_2_ generation	Carbon conversion (CO_2_ → value‐added)	—
Electrolyte environment	Solid electrolyte + pure H_2_O	Strong acid + CO_2_ feed + potassium salt	CO_2_ modifies interfacial chemistry
Cross‐over effects	Minimal	CO_2_/metal‐ion crossover affects both electrodes	Increased contamination and voltage loss risks
Cell temperature (°C)	60–80 [37, 64]	25–45	—
Cell performance (V @ A cm^−^^2^)	1.5–1.7 @ 1 [36, 37, 65]	3.1–3.6 @ 0.1–0.6 [20, 38, 66]	Higher η due to differences in reaction kinetics, interfacial phenomena, and voltage losses
Durability (≥1 A m^−^^2^)	>1000 h for lab [36, 37, 64, 65] 40 000–80 000 h for industry [67, 68]	8–750 h @ 100 mA cm^−^^2^ [20, 38, 66, 69]	Limited by catalyst degradation and ion migration
Ir loading (mg cm^−^^2^)	0.1–1 [36, 64, 70]	1–2 [20, 38, 66, 69]	Reduced loading but faster voltage decay
Operating environment	Pure O_2_ evolution	O_2_ evolution	Complex gas diffusion and pH gradients
Main limitation	Ir cost, moderate degradation	Ion crossover, electrode corrosion, electrode contamination, and difficulty in suppressing HER	Requires acid‐tolerant design
Overall implication	Mature, commercialized	Emerging technology, requiring stable acidic‐media OER electrodes	Technology gap remains

Through decades of research and development, the OER catalysts have been integrated into commercialized electrochemical systems, such as PEMWEs. Leveraging the knowledge and best practices for the OER catalysts of PEMWEs presents a rapid technological development opportunity for acidic‐media CO_2_R systems. This motivates comprehensive analysis and overview of acidic‐media OER catalysts. Accordingly, the following sections will focus on catalyst design and synthesis strategies, electrochemical performance assessment procedures, ex and in situ catalyst characterization, mechanistic insights via experimental and computational studies, degradation mechanisms and mitigation strategies, and research priorities.

## 4 Mechanistic Insights into Acidic‐Media OER

The OER is the anodic half‐reaction in PEMWEs and CO_2_R systems, supplying protons and electrons for cathodic reactions. In acidic media, OER represents the most challenging step because of its multistep proton–electron transfer process and high activation barrier. The sluggish kinetics of OER lead to large anodic overpotentials, which significantly limit the overall energy efficiency and operational stability of CO_2_ electrolyzers.

The equilibrium potential of OER is 1.23 V at 0 pH [71]. However, the actual potential to drive the cell at practical current densities (≥400 mA cm^−2^) is larger, due to the irreversible losses [72, 73]. The full‐cell voltage captures various losses, including thermodynamic potential, anode overpotential, cathode overpotential, membrane, interfaces, and Nernstian pH losses. The anode overpotential, as one of the largest contributors of the cell voltage, accounts for 0.7 V at 150 mA cm^−2^ in today's benchmark systems [58]. Minimizing voltage losses is a prerequisite to achieve breakeven energy intensity in the production of target CO_2_R products. Lower anode overpotentials require rationale catalyst design and synthesis. This, in turn, mandates understanding of OER mechanisms.

**FIGURE 2:**
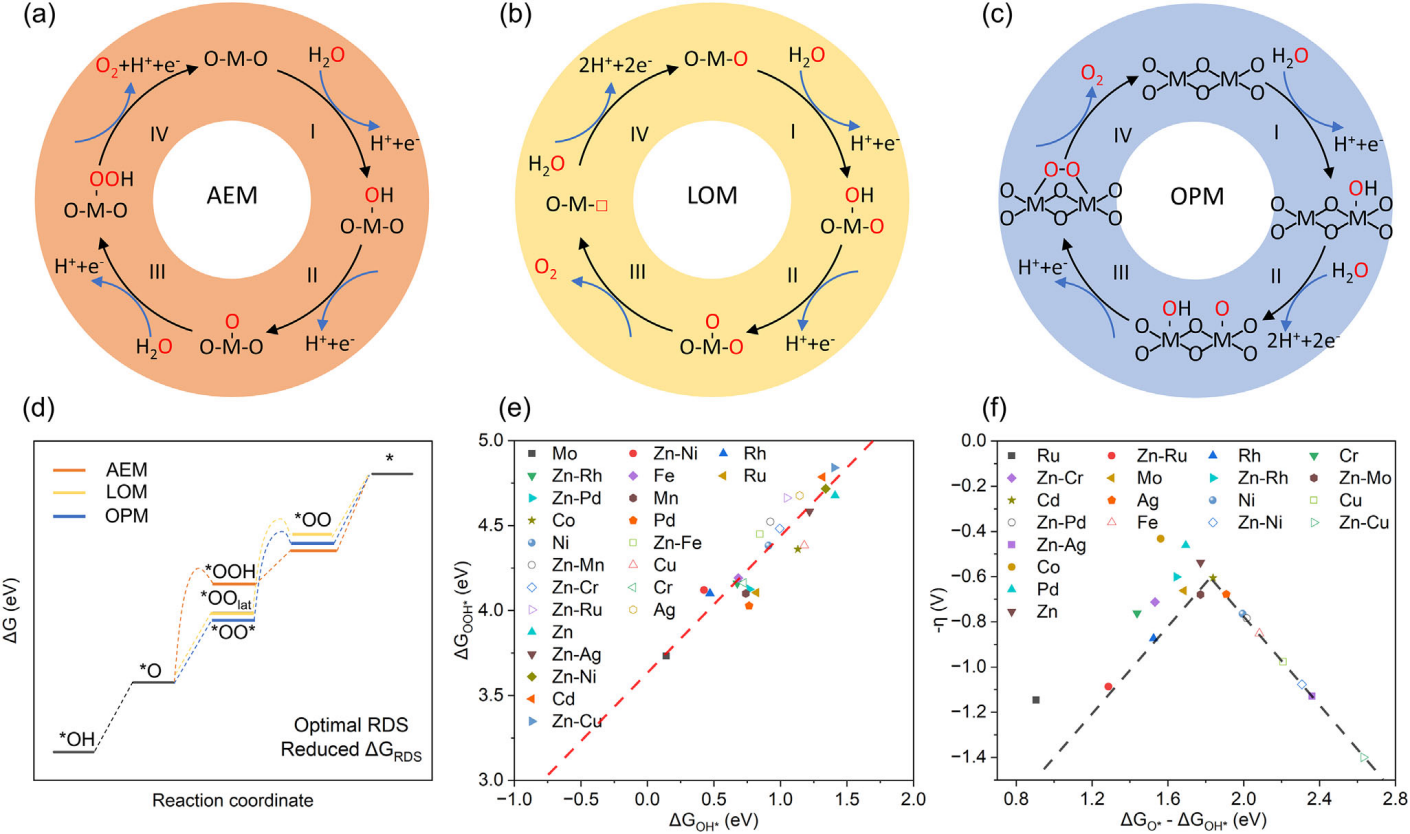
Mechanistic insights into the OER process. Common acidic‐media OER mechanisms for metal oxide‐based OER electrocatalysts of (a) AEM, (b) LOM, and (c) OPM. (a–c) Reproduced with permission [74]. Copyright 2024, Springer Nature. (d) DFT calculations based on first principles are utilized to simulate the Gibbs free energy of the reaction. Reproduced with permission [79]. Copyright 2025, Oxford University Press. (e) Linear plot between ΔG_*OOH_ and ΔG_*OH_ on the oxide surface from DFT calculations, (f) Metal oxides that leverage the difference in binding strengths of *O and *OH as activity descriptor. (e,f) Reproduced with permission [80]. Copyright 2020, Royal Society of Chemistry.

The OER is a complex process involving multiple proton‐electron transfer steps, causing high overpotentials and sluggish kinetics (Figure 2a–f). The field presently considers three primary mechanisms as the driver of acidic‐media OER: adsorbate evolution mechanism (AEM), lattice oxygen evolution mechanism (LOM), and oxide path mechanism (OPM) [74]. Each mechanism represents a unique pathway, with distinct intermediates, rate‐determining steps (RDSs), and structural implications [75, 76, 77]. The three mechanisms are not mutually exclusive; in fact, they often proceed simultaneously. For instance, AEM and LOM can concurrently contribute to OER, with their relative contributions governed by operating conditions and catalyst structure. Understanding the interplay between these mechanisms is crucial for optimizing catalyst design. Integrating diverse pathways to activate complementation between different mechanisms could accelerate the OER kinetics and improve stability [78]. Integrating the advantages of these mechanisms while mitigating their limitations could enable the development of efficient and stable acidic‐media OER catalysts.

### 4.1 Adsorbate Evolution Mechanism (AEM)

AEM, which was introduced by Nørskov et al. [81, 82], is based on density functional theory (DFT) calculations for acidic‐media OER. AEM involves four coordinated proton‐electron transfer mechanisms (Figure 2a) [83]. AEM initiates with the absorption of water molecules on the catalyst surface with oxygen‐coordinated metal sites (*), forming a hydroxyl intermediate (adsorbed *OH) via one‐electron oxidation (Figure 2a). The adsorbed *OH is oxidized with deprotonation to *O intermediate, followed by the formation of *OOH species via the interaction between water molecule and *O intermediate. The process ends with oxygen formation and active site regeneration for the next catalytic cycle via deprotonation and electron transfer. Each reaction step shows a specific energy barrier governed by the binding energies of key intermediates. The theoretical overpotential of OER is determined by the step possessing the largest free energy difference (ΔG_max_ = max[ΔG_1_, ΔG_2_, ΔG_3_, ΔG_4_]) [84].

(1) $$\Delta G_{1}=\Delta G_{*OH}-\Delta G_{water}-eU+k_{B}Tln\left(10\right)\cdot pH$$

(2) $$\Delta G_{2}=\Delta G_{*O}-\Delta G_{*OH}-eU+k_{B}Tln\left(10\right)\cdot pH$$

(3) $$\Delta G_{3}=\Delta G_{*OOH}-\Delta G_{*O}-eU+k_{B}Tln\left(10\right)\cdot pH$$

(4) $$\Delta G_{4}=4.92\;eV-\Delta G_{*OOH}-eU+k_{B}Tln\left(10\right)\cdot pH$$

where ΔG is the Gibbs free energy of the reaction step and U is the potential vs. normal hydrogen electrode (NHE) under standard conditions (P = 1 bar, T = 298.15 K, pH = 0). Nernst equation (∆G_H+_ (pH) = –k_B_T ln (10)∙pH) represents the protons’ free energy change in a specified electrode at pH ≠ 0 [85]. A constant value of –2$\Delta G_{H2O}^{exp}$ = 4 × 1.23 = 4.92 eV is typically considered [86, 87]. The Gibbs free energy of each step is governed by the adsorption energy of intermediates, *OOH, *OH, and *O. An ideal catalyst should deliver equal free energy changes, initiating the OER above the equilibrium potential of 1.23 V. The potential determining step (PDS) is governed by the reaction energy barrier of the oxidation process from *OH to *O or *O to *OOH (i.e. ΔG_2_ or ΔG_3_) [43, 87]. There exists a linear correlation between the adsorption free energies of *OOH and *OH (ΔG_*OOH_ = ΔG_*OH_ + 3.2 eV) [88]. This causes a theoretical overpotential threshold of 370 mV (η= ΔG_max_/e‐1.23 V). While the scaling correlation enables rapid catalyst screening, it restricts catalytic efficiency by setting a minimum overpotential limit (Figure 2e). Sabatier principle suggests that an ideal catalyst must strike a balance between strong and weak adsorption of reaction intermediates [89]. In the context of acidic‐media OER, this means that the catalyst should bind oxygen intermediates (*OH, *O, and *OOH) neither too strongly nor too weakly [90]. Excessively strong binding induces the blockage of active sites by intermediates, inhibiting further reaction progression. Excessively weak binding limits the adsorption of reactants, reducing reaction rate. This trade‐off creates a volcano‐shaped activity relationship (Figure 2f).

DFT calculations provide insights into catalytic activity by estimating the adsorption energies of reaction intermediates (Figure 2d). Acidic‐media OER catalysts are typically assessed based on their oxygen binding energy (ΔG_*O_), which follows a similar volcano‐shaped trend as HER catalysts do with hydrogen binding energy (ΔG_*H_) [91]. An ideal OER catalyst shows an intermediate ΔG_*O_. DFT models primarily focus on thermodynamic factors, neglecting kinetic and environmental influences, such as solvent interactions and interfacial electric fields. Thus, DFT‐predicted volcano plots require experimental validation. Despite these limitations, AEM could still guide the design of stable and efficient acidic‐media OER catalysts. AEM analysis could also reveal catalyst deactivation mechanisms, including activity and stability loss (catalyst poisoning) caused by poorly desorbed intermediates. Additionally, AEM motivates the design of catalytic activity promotion and deactivation mitigation strategies, such as tuning d‐band center and oxygen adsorption energies, strain modulation, and doping [92]. The AEM's insights have not only shaped the development of noble metal‐based catalysts like Ru and Ir oxides but have also informed innovations in non‐noble and composite systems.

### 4.2 Lattice Oxygen Evolution Mechanism (LOM)

LOM is a distinctive pathway that differs from AEM in the origin of oxygen production and reaction dynamics [93]. In LOM, oxygen is generated not only from water molecules but also from the lattice oxygen atoms within the catalyst structure (Figure 2b) [94]. LOM and AEM share the same first two steps, including *O formation. In LOM, *O combines with the lattice oxygen to release an oxygen molecule, leaving an oxygen vacancy in the lattice [95]. The oxygen vacancy is refilled by deprotonation of the absorbed water molecule [96]. This mechanism facilitates the direct O–O coupling between the lattice oxygen and the adsorbed intermediates, breaking the scaling limitations concomitant with AEM. Accordingly, the LOM‐based electrocatalysts surpass theoretical overpotential limitations, achieving higher activity [97, 98]. The lattice oxygen participates in the entire process as an active site. The catalyst undergoes dynamic equilibrium process during the reaction, and the replenishment of lattice oxygen can be identified with on‐line differential electrochemical mass spectrometry (DEMS) measurements of the ^18^O‐labelled catalyst [83, 99].

Despite offering activity advantages, the LOM‐based catalysts typically encounter stability challenges. The migration of lattice oxygen creates vacancies, destabilizing neighboring metal sites and causing metal dissolution and structural collapse [85]. The stability challenges are particularly pronounced in acidic media where the catalyst becomes more vulnerable to degradation. Addressing the stability trade‐off requires strategies that could prevent structural disintegration. Incorporating protective layers or anchoring catalysts onto stable substrates are good representatives of such strategies [86, 87]. LOM and AEM can proceed simultaneously. The relative contribution of each mechanism is governed by the catalyst specifications and operating conditions [88]. LOM provides an insightful framework for the design of active and efficient acidic‐media OER catalysts. LOM also leverages lattice oxygen dynamics and addresses stability issues associated with oxygen vacancy formation. Overall, optimizing structural features and integrating protective measures are critical for the design of efficient and durable LOM‐based OER catalysts for acidic‐media CO_2_R.

### 4.3 Oxide Path Mechanism (OPM)

OPM is another mechanistic pathway in OER. OPM proceeds via direct O–O radical coupling without involving the lattice oxygen or additional reactive *OOH intermediates [89, 90]. In OPM, the water molecules produce *OH intermediates on adjacent metal active sites through proton‐coupled electron transfer (PCET) (Figure 2c) [91, 93]. These intermediates deprotonate to form *O radicals, which then undergo direct O─O coupling to release oxygen. OPM — by avoiding the lattice oxygen participation and oxygen vacancy creation — ensures high OER activity and structural stability. Thus, OPM addresses the major limitation of LOM [93, 94, 95, 96]. However, OPM requires specific geometric configurations for its pathway to be effective. Symmetric bimetallic sites with optimal interatomic distances could facilitate O─O coupling with lower energy barriers [93]. For example, the Ru‐array patches supported by MnO_2_ exhibit shortened Ru─Ru distances, favoring the OPM pathway and enhancing catalytic activity and stability [93]. OPM involves only M─O and M─OH intermediates [97]. Thus, OPM — compared to AEM and LOM — simplifies the reaction steps and reduces energy barriers, improving efficiency [100]. However, designing metal sites with the geometric precision mandated by OPM remains a challenge. The OPM pathway is more suitable for well‐crystallized metal oxides with minimal defects [64]. In contrast, LOM becomes active in amorphous structures with abundant oxygen vacancies [99, 101]. OPM bridges the activity‐stability trade‐offs concomitant with AEM and LOM. OPM — by ensuring high activity and structural integrity — offers a promising pathway to efficient and durable acidic‐media OER catalysts. Leveraging OPM through rationale design of advanced OER catalysts would provide an efficient pathway to acidic‐media OER, and hence acidic‐media CO_2_R.

### 4.4 Dominant Pathways and Performance‐Determining Factors

Under acidic conditions, stable noble‐metal oxides, such as IrO_2_ and RuO_2_, typically follow AEM, in which OER proceeds through adsorbed intermediates on the metal surface [102, 103, 104]. Catalysts possessing high metal–oxygen covalency or defect‐rich lattices often exhibit pronounced contribution from LOM [105, 106, 107], whereas systems containing ordered dual‐metal active sites with low defect density may trigger OPM [108]. The predominance of one pathway over another is governed by a combination of intrinsic factors—including electronic structure, lattice strain, and defect concentration—and extrinsic factors, such as applied potential, temperature, pH, and electrolyte composition [109, 110].

The acidic‐media OER activity is closely correlated with the electronic configuration and adsorption energetics of the catalyst, particularly the e_g_ orbital occupancy and the position of the O 2p band relative to the Fermi level [111, 112, 113]. Moderate metal–oxygen bond covalency provides optimal adsorption energies for *OH, *O, and *OOH intermediates, enabling faster kinetics and longer stability [114, 115]. In contrast, excessive covalency favors lattice‐oxygen redox activity and accelerates dissolution, whereas insufficient covalency causes sluggish PCET and poor activity [116, 117].

Defects, amorphization, and surface reconstruction also play critical roles in activity and durability [109, 118]. Structural defects and amorphous domains introduce unsaturated metal sites that facilitate water adsorption and intermediate but at the expense of poorer structural integrity. For instance, amorphous hydrous IrO_x_ typically exhibits higher OER activity but dissolves more rapidly than crystalline IrO_2_ [119, 120]. Likewise, oxides rich in oxygen vacancies or subject to lattice strain tend to promote the LOM pathway, enhancing activity but typically at the expense of stability [105, 121].

Composition and structure engineering offer a powerful means to tune the M─O bond strength and electronic structure [122]. Alloying or doping, by combining Ir or Ru with Co, Ni, or Fe, or introducing Ti, W, or Sn into the matrix of Ir and Ru, can balance activity, stability, and cost [123, 124, 125, 126, 127]. Single‐atom catalysts (SACs) and high‐entropy oxides (HEOs) maximize noble‐metal utilization while providing synergistic multi‐metal active environments [128, 129, 130]. Conductive supports, such as TiN and doped carbides, further strengthen interfacial bonding, suppress metal dissolution, and stabilize active sites under high potentials [131, 132].

Lastly, operational parameters markedly influence catalytic behavior and degradation [109]. High anodic potentials promote the formation of high‐valent species (i.e., IrO_4_ and RuO_4_) that accelerate oxidative dissolution [133]. Elevated temperatures enhance ion mobility and lattice rearrangement, while electrolyte composition and local pH modify adsorbate binding and surface reconstruction [109]. A comprehensive understanding of these dynamic interactions is essential to steer the OER toward favorable mechanistic pathways while maintaining structural integrity and extending catalyst lifetime.

Taken together, the dominance and transition among AEM, LOM, and OPM under acidic‐media OER conditions can be understood as a dynamic outcome of catalyst chemistry and operating environment rather than a fixed mechanistic choice. In general, AEM is favored on stable, well‐crystallized noble‐metal oxides with moderate metal–oxygen covalency, where optimal adsorption energies of *OH, *O, and *OOH intermediates enable PCET without significant lattice involvement. As metal–oxygen covalency increases or defect density rises, lattice oxygen becomes more redox‐active, lowering the energetic barrier for oxygen vacancy formation and promoting the contribution of LOM, albeit often at the expense of stability loss. In contrast, OPM becomes more competitive in systems featuring ordered dual‐metal active sites and well‐defined geometric configurations, where direct O–O coupling between adjacent *O species can proceed without lattice oxygen participation. Importantly, these pathways are not mutually exclusive: elevated anodic potentials, pH‐dependent proton–electron transfer kinetics, and operando surface reconstruction can continuously shift the relative contributions from predominantly AEM to mixed AEM/LOM behavior and, in extreme cases, to pronounced LOM or OPM contributions. In practical electrolyzers, such elevated anodic potentials are typically required to sustain high operating current densities, implying that mechanistic contributions identified under mild conditions may evolve under high‐current operation. This framework implies that pathway transitions in acidic‐media OER might be governed by electronic structure, lattice dynamics, intermediate adsorption energetics, and reaction conditions.

### 4.5 Representative Case Studies of AEM–LOM Interplay

Recent studies have further clarified how structural and electronic modulation can steer the OER pathway between the AEM and LOM in acidic media [134, 135].

For instance, the OER primarily proceeds via LOM, which is typically detrimental to catalyst stability due to lattice oxygen depletion. To counteract this limitation, the AgRuIr catalyst was engineered with stepped disconnections and a crack‐patterned oxide shell [134]. These structural motifs enhance water adsorption and activation, facilitating the replenishment of lattice oxygen through OH^–^ ions derived from water, as verified by operando analyses and DFT calculations [136, 137]. Moreover, Ag atoms anchored at dislocation sites increase the diffusion energy barrier for lattice migration and metal dissolution, yielding a synergistic balance between activity and structural robustness under acidic‐media OER conditions [134].

In contrast, Zhao et al. demonstrated that Ru‐based catalysts, which are typically associated with the LOM pathway due to their strong Ru─O covalency, can be tuned to follow the AEM pathway through strain engineering [135]. DFT calculations showed that tensile strain downshifts the Ru 4d‐band center, weakens the Ru─O bonding, and optimizes the adsorption energies of *OH, *O, and *OOH intermediates [138]. The RDS was identified as the *O → *OOH, consistent with the AEM pathway. Operando spectroscopic characterization and kinetic isotope effect (KIE) measurements confirmed the absence of lattice oxygen participation, supporting a PCET‐dominated mechanism [135]. This strain‐engineered modulation not only enhances intrinsic activity but also suppresses Ru dissolution and lattice oxygen loss, enhancing durability in acidic environments.

These two cases collectively underscore the decisive role of metal–oxygen covalency and lattice strain in governing OER pathways. Fine‐tuning of these parameters could balance catalytic activity and structural stability, offering practical guidance for the design of high‐performance and stable OER catalysts.

### 4.6 Mechanistic Distinctions between Noble‐Metal and Non‐Noble‐Metal Catalysts

Noble‐metal oxides, such as IrO_2_ and RuO_2_, predominantly follow the AEM pathway under acidic conditions [139]. Their moderate metal–oxygen covalency ensures optimal adsorption of *OH, *O, and *OOH intermediates, providing high activity and superior stability [114]. RuO_2_ exhibits stronger covalency and thus higher intrinsic activity but suffers from partial lattice‐oxygen participation and dissolution through the formation of volatile RuO_4_ at high potentials [140, 141]. Alloying or doping (e.g., Ru─Mn, Ir─Ti, and Ir─Ni) can effectively modulate M─O covalency, stabilizing the lattice oxygen and mitigating over‐oxidation [127, 142].

In contrast, non‐noble‐metal oxides (i.e., Co‐, Fe‐, and Mn‐based systems) exhibit stronger M─O covalency and abundant oxygen vacancies, which promote LOM [143, 144]. While the promotion of LOM enhances activity by breaking the AEM scaling limitations, it also accelerates structural degradation in acidic electrolytes [108, 145]. Surface passivation layers (i.e., TiO_2_, Ta_2_O_5_, and Nb_2_O_5_) or heterostructure design are typically required to suppress dissolution while maintaining electronic conductivity [146]. Integrating trace amounts of noble metals at the interface has proven effective in stabilizing base‐metal frameworks through strong interfacial bonding and optimized electron transfer [147].

Overall, the mechanistic distinction between noble‐ and non‐noble‐metal catalysts highlights the trade‐off between activity and durability dictated by metal–oxygen covalency. Understanding and leveraging this relationship provides the theoretical foundation for the rational design of next‐generation acidic‐media OER catalysts, as further detailed in Section 6.

### 4.7 Theoretical Modeling and Operando Correlation in Acidic‐Media OER

Understanding the acidic‐media OER requires a synergistic integration of first‐principles theoretical modeling and operando experimental validation. DFT studies remain the most widely adopted computational framework for establishing atomic‐level relationships between catalyst structure, surface energetics, and activity trends [148, 149, 150].

DFT‐based modeling enables the quantitative construction of reaction free‐energy diagrams by evaluating the adsorption energies and Gibbs free‐energy changes (ΔG) of key intermediates (i.e., *OH, *O, and *OOH) [151]. Such analyses identify the PDS/RDS (in thermodynamic sense), thereby predicting limiting potentials (overpotentials). These approaches can also be practical for estimating the reaction barriers when coupled with explicit kinetic treatments, such as the nudged elastic band (NEB) method, ab initio molecular dynamics (AIMD) simulations, or microkinetic modeling, as demonstrated in recent DFT/metadynamics and classical OER studies [152, 153, 154, 155, 156]. By establishing linear scaling relationships between intermediate adsorption energies, DFT studies could link thermodynamics with activity trends and provide a unified framework to interpret how dopants, strain, or electronic modifications modulate metal–oxygen covalency and intermediate binding [157, 158, 159]. While these DFT‐based thermodynamic and kinetic analyses are powerful for identifying activity trends and mechanistic preferences, they do not, by themselves, guarantee direct guidance for experimental catalyst synthesis under acidic‐media OER conditions.

Within this framework, DFT studies underpin multiple interrelated applications in acidic‐media OER: (i) mechanistic elucidation, by comparing the thermodynamic feasibility of AEM, LOM, and OPM pathways in conjunction with operando evidence to assess the dominant O─O formation route; [74] (ii) descriptor‐based screening, where ΔG_*O_ or ΔG_*OOH_ values guide high‐throughput discovery; [87] (iii) electronic‐structure optimization, quantifying how alloying, heterostructuring, or single‐atom dispersion regulate M─O covalency and stability [160, 161, 162]; and (iv) model–experiment benchmarking, in which DFT‐predicted limiting potentials and intermediate configurations are compared with Tafel slopes, onset potentials, and operando spectroscopic fingerprint [119, 163]. Representative examples include the dynamic structural modulation strategy proposed by Zhang et al. [164], which alleviates conventional scaling constraints, and the strain‐ or dopant‐induced Ru─O bonding optimization demonstrated by Liu et al. [165]. Among these applications, electronic‐structure optimization and model–experiment benchmarking are generally more directly translatable to experimental synthesis, as they often yield specific compositional, dopant, or structural design targets. In contrast, mechanistic elucidation and descriptor‐based screening primarily provide qualitative or semi‐quantitative insights that define activity trends and design principles rather than synthesis‐ready prescriptions.

Despite their predictive power, DFT investigations remain an approximation to the real electrochemical interface [166]. Most calculations are performed at static 0 K reference conditions, typically under vacuum or within implicit solvation and fixed‐slab approximations. Thus, such studies typically neglect the critical phenomena, including explicit solvation, interfacial electric fields, and electrode‐potential dependence [148, 167, 168]. These simplifications also fail to capture the dynamic three‐phase microenvironment involving solvent reorganization, hydrogen‐bond fluctuations, and entropy–enthalpy compensation at the electrode–electrolyte interface [26, 27]. In addition to such interfacial approximations, limitations also arise from the treatment of electronic structure. Conventional generalized gradient approximation (GGA)‐level exchange–correlation functionals, while widely applied, often exhibit limited accuracy in describing the electronic structures and metal–oxygen bonding of Ru‐ and Ir‐based oxides, leading to functional‐dependent variations in calculated energetics and motivating the adoption of hybrid or beyond‐DFT approaches [169, 170]. Computational cost constraints also restrict system size, often omitting surface defects, large‐scale reconstruction, and explicit solvent dynamics, all of which can significantly affect OER energetics [118, 171, 172].

These discrepancies highlight that many conventional DFT predictions remain qualitative or exploratory when applied to acidic‐media OER, particularly in the absence of explicit treatment of surface reconstruction, dissolution, and electrochemical potential effects. Notably, several representative discrepancies between static DFT predictions and operando observations have been widely observed for acidic‐media OER systems, underscoring the limitations of conventional modeling assumptions. For example, slab‐based DFT calculations typically assume structurally rigid oxide surfaces with fixed active sites, whereas operando spectroscopic and microscopic studies (e.g., operando X‐ray absorption spectroscopy (XAS), Raman spectroscopy, and transmission electron microscopy (TEM)) often reveal surface reconstruction, oxygen enrichment, and even partial metal dissolution for RuO_2_‐ and IrO_2_‐based catalysts at OER potentials. Likewise, conventional DFT analyses often favor the AEM based on the thermodynamic stability of *OH, *O, and *OOH intermediates, while operando isotope‐labeling and spectroscopic measurements typically show direct lattice oxygen participation, particularly for defect‐rich or highly covalent oxides. In addition, activity trends inferred from ΔG‐based volcano relationships occasionally deviate from experimentally observed potential‐ and time‐dependent activity evolution, highlighting the critical roles of interfacial electric fields, solvent reorganization, and coverage‐dependent energetics that are absent in fixed‐charge, fixed‐structure DFT treatments.

To overcome these inherent limitations, recent theoretical advances have focused on improving the physical realism of electrochemical interface models and the accuracy of electronic‐structure descriptions [166]. The implementation of explicit interface and constant‐potential frameworks, often coupled with AIMD, enables realistic simulations that account for solvent molecules, solvated ions, and interfacial electric fields, thereby capturing potential‐dependent solvation structures and dynamic hydrogen‐bond network reorganization at the electrode–electrolyte interface [168, 173, 174]. By explicitly incorporating electrode potential, pH effects, and interfacial solvation, these advanced theoretical frameworks substantially improve the experimental relevance of DFT predictions and move computational screening closer to actionable guidance for catalyst synthesis.

In addition to potential‐dependent modeling, incorporating pH effects has become increasingly important for accurately describing electrochemical interfaces. Conventional DFT simulations typically treat the proton chemical potential via the computational hydrogen electrode (CHE) approximation, which neglects explicit protonation–deprotonation equilibria under varying pHs [175, 176]. To address this limitation, grand‐canonical and constant‐pH DFT frameworks have been developed to enable dynamic control on electron chemical potential, and in emerging approaches, proton activity, thereby extending traditional fixed‐charge DFT assumptions to more realistic electrochemical conditions. In particular, constant‐potential and constant inner‐potential (CIP) DFT methods allow simulations under fixed electrode potentials by treating the electron number as a thermodynamic variable, while fully converged constant‐potential (FCP) DFT and related grand‐canonical implementations achieve self‐consistent potential convergence [166, 177, 178]. When these frameworks are coupled with explicit‐solvent or constant‐potential AIMD simulations, they capture potential‐ and pH‐dependent solvation and interfacial charge responses, revealing how electrolyte acidity modulates surface charge distribution, adsorption energetics, and PCET kinetics at the electrode–electrolyte interface [174, 179, 180].

In parallel, the limitations of conventional GGA‐level DFT in describing localized d states and metal–oxygen bonding of transition‐metal oxides have been progressively mitigated through the adoption of more advanced electronic‐structure approaches. Methods, such as DFT plus Hubbard U (DFT+U) and meta‐generalized gradient approximation (meta‐GGA) functionals, help correct self‐interaction and correlation errors, while hybrid functionals and constrained random‐phase approximation (cRPA) techniques provide more accurate treatments of exchange and screening effects. These approaches have been successfully applied to oxides like RuO_2_ and IrO_2_ to improve electronic‐structure predictions and reduce functional‐dependence errors [139, 181, 182].

Collectively, these advances are transforming DFT simulations from idealized thermodynamic approximations into quantitatively predictive and experimentally grounded frameworks while clarifying that many conventional DFT studies provide primarily qualitative or exploratory insights, and that integrated activity–stability modeling under realistic electrochemical conditions represents the most promising route toward synthesis‐guiding computational predictions for acidic‐media OER catalysts.

## 5 Electrochemical Performance Evaluation

The electrochemical performance evaluation elucidates the activity, overpotential, and stability of OER catalysts. A complete assessment requires well‐defined performance metrics and measurement protocols. Cyclic voltammetry (CV) and linear sweep voltammetry (LSV) analyses are typically used to quantify key parameters, including overpotential, onset potential, electrochemically active surface area (ECSA)‐normalized current density, turnover frequency (TOF), Tafel slope, and exchange current density. Insights into long‐term catalytic activity and stability can be obtained via chronoamperometry (CA), chronopotentiometry (CP), and repeated CV.

While half‐cell measurements provide access to intrinsic OER kinetics under well‐defined electrochemical conditions, they do not fully capture the complexity of device‐level operation. In full‐cell configurations, such as slim flow cells or MEA electrolyzers, additional phenomena—including oxygen bubble accumulation, mass transport limitations (within thick CLs), membrane–electrode interfacial interactions, and local proton activity gradients—introduce extra voltage losses that are typically minimum in conventional three‐electrode measurements. Accordingly, half‐cell and full‐cell evaluations should be regarded as complementary: the former establishes intrinsic kinetic benchmarks, whereas the latter reflects the extent to which such intrinsic activity can be realized under practical operating conditions.

Full‐cell measurements further enable optimization of system‐level parameters, including electrolyte identity and composition, cell temperature, and electrolyte flow conditions. The following section overviews the electrochemical measurement techniques, best practices for catalyst benchmarking, and methodologies for performance testing. Particular attention is given to how the interpretation of activity and stability metrics depends on the applied current‐density regime, which is critical for bridging half‐cell benchmarking and device‐relevant operation.

### 5.1 Half‐Cell Measurements

Accurate performance evaluation requires control of key factors, including impurity contamination, reference electrode stability, uncompensated resistance, working electrode preparation, and mass‐transfer effects. In this context, half‐cell measurements are primarily employed to probe the intrinsic OER activity and kinetics of catalysts under controlled and simplified environments, in lieu of directly representing their performance in full‐cell devices. Reproducible and reliable performance and stability assessments require proper selection of vessel configuration, electrolytes, and electrodes.

Three‐electrode setup enables accurate measurement of intrinsic activity and overpotential. A three‐electrode cell is composed of a working electrode (WE), a counter electrode (CE), a reference electrode (RE), and a gas inlet [111]. A transparent glass cell shows chemical resistivity to acidic conditions while enabling direct observation into reaction volume. During the measurement, the electrolyte is saturated with high‐purity oxygen to fix the reversible oxygen potential [112]. The saturation of the electrolyte with oxygen enables maintaining the equilibrium potential at the standard value, i.e., 1.23 V vs. reversible hydrogen electrode (RHE) for OER. The gas saturation process is critical, as the partial pressure of the introduced gas enters the Nernst equation. The Nernst equation for OER can be described as follows [183]:

(5) $$E_{OER}=E_{OER}^{0}-0.059\times pH+\frac{RT}{4F}\ln\left(P_{O2}\right)$$

where *E_OER_* is the equilibrium potential for OER, $E_{OER}^{0}$ is the standard potential for OER (1.23 V vs. RHE at 25°C), *R* is the universal gas constant (8.314 mol^−1^ K^−1^), *T* is the temperature (K), *F* is the Faraday constant (96485 C mol^−1^), and *P*_*O*2_ is the partial pressure.

Half‐cell measurements are typically performed in a three‐electrode setup using a CE, a RE, and a WE. Pt wires, foils or meshes are widely used as the CE to sustain large current, balancing the charge at the WE. The CE should possess a much larger projected area than the WE, ensuring that the reaction rate is not limited by the CE [114]. The CE should be placed nearby the WE to ensure homogeneous electric field [115]. The potential of the WE is measured with respect to the stable potential of the RE. The composition and concentration of both electrolytes should be the same to obtain credible data from the WE. A commercial or custom‐made RE is recommendable. Despite the availability of various REs (i.e., Hg/Hg_2_SO_4_ and Ag/AgCl) [74, 184, 185, 186], RHE is more commonly used in acidic‐media OER experiments. The RHE can go through facile preparation, which involves fusing one end of a glass tube, sealing a Pt wire inside as the RHE, filling the tube with acidic electrolyte, and generating hydrogen through electro‐water splitting [187, 188, 189]. The RE potential can alter if the hydrogen concentration is not sufficiently maintained, or the dissolved species from the WE or CE reach the RE [190]. The RE should be calibrated before electrocatalytic measurements. The recorded potential is converted to the RHE potential for fair comparison [43, 74, 191, 192]. The measured potentials are calibrated to the RHE scale as follows [193]:

(6) $$E\left(\mathrm{vs}.\;\mathrm{RHE}\right)=E\left(\mathrm{vs}.\;\mathrm{RE}\right)+E\left(\mathrm{calib}\right)$$

where E(calib) is the RE potential calibrated to RHE and E(calib) is the CV at a low scan rate of 1–5 mV s^−1^ in hydrogen‐saturated electrolyte. CV analysis is conducted in the voltage range of hydrogen electrocatalysis (HER/HOR) using Pt wires or foils as the CE and WE [194]. The HER/HOR CV possesses two voltage intercepts at zero current, and E(calib) is obtained by averaging them.

**FIGURE 3:**
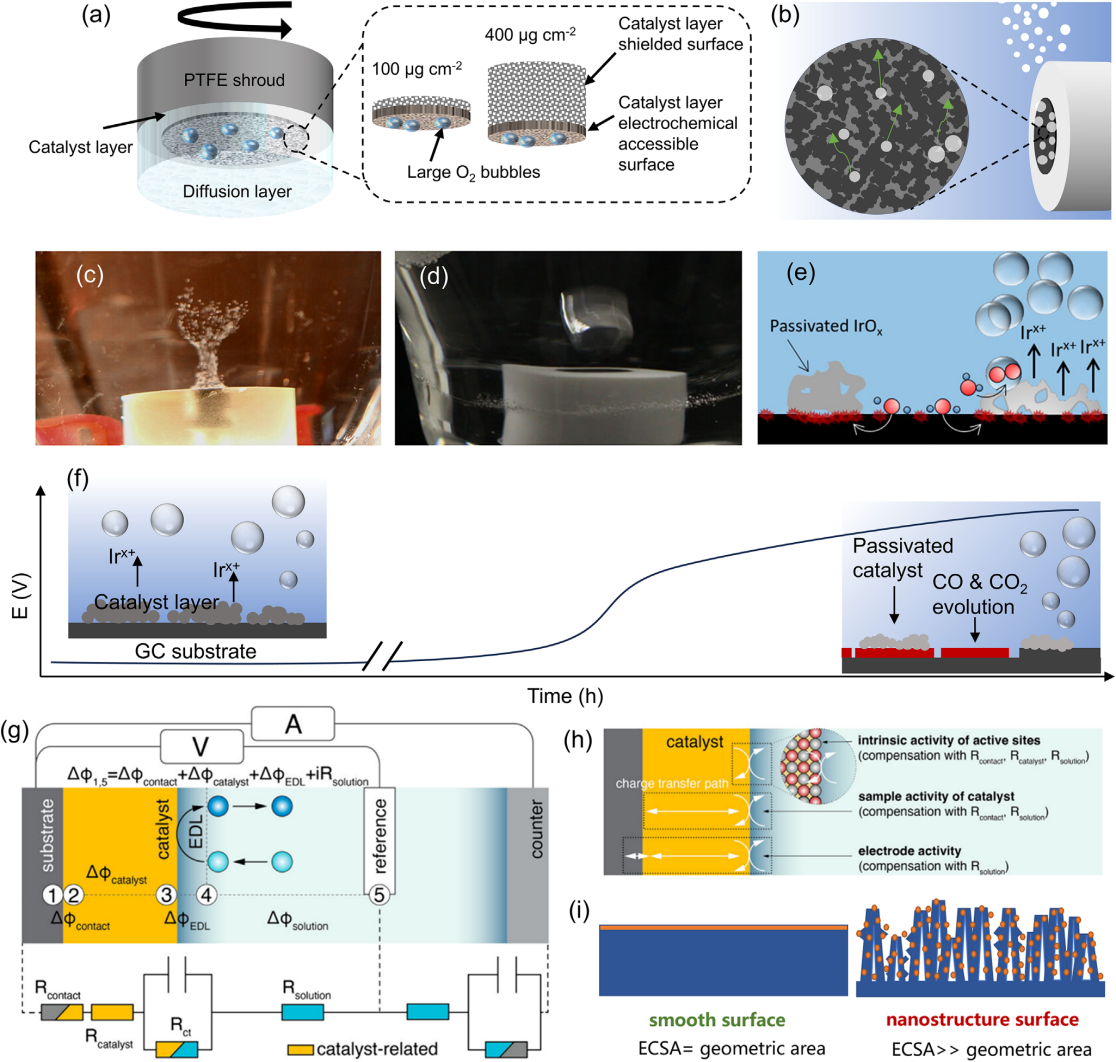
Schematic overview of key experimental factors affecting GC/RDE‐based OER measurements. (a) Schematic illustration of microscopic bubble accumulation within the CLs of various catalyst loadings during potential cycling. Reproduced with permission [198]. Copyright 2022, American Chemistry Society. (b) The release of small bubbles trapped inside the catalyst network. Reproduced with permission [197]. Copyright 2023, American Chemistry Society. (c) Bubble removal on an inverted RDE without forced convection, and (d) with forced convection. (e) Small catalyst clusters become passivated through electrical detachment from the GC substate, and the still active catalyst domains undergo harsher oxidizing conditions leading to higher local catalyst dissolution. (f) Degradation mechanism of the GC backing substrate in the course of galvanostatic OER electrocatalyst testing. (c–f) Reproduced with permission [199]. Copyright 2024, American Chemistry Society. (g) Schematic representation of potential drop across a simplified three‐electrode system with a modified WE and the corresponding equivalent circuit diagram: (1) surface of the substrate; (2) surface of the catalyst contacting the substrate; (3) surface of the catalyst contacting the electrolyte solution; (4) outer boundary of the diffuse layer; (5) junction of the RE. (h) Schematic representation of a modified electrode and the region of interest (dotted box) with different definitions of activity. White arrows represent the charge transfer path. (g,h) Reproduced with permission [208]. Copyright 2023, American Chemical Society. (i) Illustration of ECSA and geometric area.

Glassy carbon (GC) electrodes, i.e., static GC electrode and rotating disk electrode (RDE), are used as the WE. GC electrodes are typically decorated with an ink‐casting method. Considering the oxygen bubble accumulation and mass transfer limitations (Figure 3a), RDEs are tested using a standard OER measurement protocol [195, 196, 197]. However, the potential increase during RDE stability tests is not necessarily due to the catalyst degradation but it could rather result from the accumulation of nano‐ and micro‐bubbles within the porous CL [195, 198, 199]. The bubbles block the active sites, causing activity loss (Figure 3b). The state‐of‐the‐art approaches for bubble removal include sonication in the vicinity of the WE, electrolyte flow through the CL, magnetic stirring, and inverse RDE (Figure 3c,d) [197, 199]. WEs are prepared using a homogeneous catalyst ink consisting of catalyst particles, polymeric binder, and organic solvents [200]. In some cases, the catalyst ink can also include carbon or acetylene black to improve suspension by preventing the precipitation of metal oxides [201, 202]. Herein, carbon black can also increase the electrical connection within the CL [187, 203, 204]. Unfortunately, carbon particles tend to oxidize and degrade under oxidative conditions (Figure 3e,f) [205]. The catalyst loading on the WE needs to be optimized, as only the outermost layer participates in OER (Figure 3a) [198]. Low catalyst loadings typically cause poor catalytic‐layer coverage and electronic contact. High catalyst loadings, however, could cause aggregation and performance loss [206]. The loading of the polymeric binder should also be optimized to prevent catalyst detachment and improve mass and electron transport [207].

*iR* compensation is applied to account for the ohmic drop caused by the resistance of the electrolyte and other cell components. Capturing this resistance otherwise would complicate the activity comparisons of the catalysts in different setups. The *iR* drop originates from the electrolyte resistance (R_solution_, due to the ion transport in electrolyte), contact resistance (R_contact_, due to the interface of catalyst and electrode support), and CL resistance (R_catalyst_, due to the resistance within catalyst material) (Figure 3g) [209, 210, 211]. Reliable *iR* compensation requires measuring and correcting for resistance while minimizing artifacts. The first step is to determine total resistance via electrochemical impedance spectroscopy (EIS). This involves performing EIS measurements over a frequency range of 100 kHz to 0.1 Hz and identifying the high‐frequency resistance (R_HFR_), which corresponds to the sum of electrolyte resistance (R_solution_), contact resistance (R_contact_), and CL resistance (R_catalyst_) (Figure 3h) [212]. The phase angle closest to zero in a Nyquist plot indicates the correct value for compensation [213]. Following the identification of the resistance, the next step is to apply an appropriate level of *iR* compensation. 100% compensation could eliminate all resistance effects, recommendable for intrinsic catalyst activity studies. However, the common practice is to use partial compensation, typically 85%, to prevent overcorrection [208], which can introduce artifacts. For electrode stability studies, only the electrolyte resistance (R_solution_) is corrected, ensuring that the contact and CL resistances remain part of the analysis. An optimized cell configuration reduces the *iR* drop, improving measurement accuracy. Shortening the distance between WE and RE reduces the ion‐transport resistance. Conductive substrates lower the contact resistance (R_contact_). Similarly, high‐purity electrolytes reduce impurity‐related resistance variations [208, 213].

Despite their utility in establishing intrinsic kinetic descriptors, half‐cell measurements inherently neglect several factors that become dominant at the device level. In full‐cell operation, OER occurs within thick CLs interfaced with polymeric films, where oxygen bubble removal, proton and water transport, ionomer distribution, and membrane–electrode contact resistance significantly influence the apparent anode overpotential. Moreover, local proton activity in full‐cell environments can deviate from bulk electrolyte values due to coupled anodic and cathodic reactions, leading to behavior that cannot be reproduced in conventional three‐electrode setups. Therefore, half‐cell results should be interpreted as an upper bound for anode kinetics, while full‐cell testing is required to assess CL utilization and transport‐induced losses under realistic operating conditions. In this context, stability tests conducted in half‐cell configurations are most commonly performed at low current densities (typically around 10 mA cm^−^^2^), where mass‐transport limitations, gas‐evolution‐induced mechanical stresses, and local chemical gradients are minimized. While such conditions are valuable for probing intrinsic electrochemical robustness and enabling cross‐study comparisons, they do not reproduce the coupled transport, interfacial, and mechanical stresses encountered at device‐relevant current densities (≥100 mA cm^−^^2^). Consequently, stability trends derived from low‐current half‐cell measurements should be regarded as indicative rather than predictive of catalyst durability under practical electrolyzer operation.

### 5.2 Key Performance Parameters and Measurement Methods

#### 5.2.1 Overpotential (η)

Overpotential represents the additional voltage required to drive the OER beyond the thermodynamic equilibrium potential of 1.23 V vs. RHE. Overpotential can be measured at various current densities, such as 10 mA cm^−2^ (η_10_, catalyst benchmarking) or greater for industrial relevance [214]. Low overpotentials are crucial for reducing energy losses. The overpotential is derived from CV or LSV measurements, where the current is normalized to the electrode's geometric surface area. This metric has limitations, as electrocatalysts could possess heterogeneous surfaces with pores, steps, and structural defects [215, 216]. Since these irregularities influence catalytic activity, normalizing current density based on ECSA could enable higher accuracy.

Unless otherwise specified, overpotentials reported in the literature are predominantly obtained from half‐cell measurements and therefore reflect intrinsic catalytic properties rather than realizable full‐cell performance. In particular, while η measured at 10 mA cm^−^^2^ (η_10_) is widely used for catalyst benchmarking in half‐cell studies, this low‐current metric does not capture transport‐ and bubble‐related losses that dominate full‐cell operation at industrially relevant current densities. Accordingly, both activity and stability metrics reported at low current densities should be interpreted within the context of intrinsic catalyst screening, whereas performance and durability at higher current densities require complementary high‐current or device‐level evaluations.

#### 5.2.2 Electrochemical Active Surface Area (ECSA)

ECSA estimates the electrochemically accessible surface area, which is often used as the representative of active site density (Figure 3i). It is commonly determined using capacitance *C_dl_* from CV measurements in a non‐Faradaic region according to:

(7) $$ECSA=\frac{C_{dl}}{C_{s}}$$

where *C_dl_* is measured from the scan‐rate‐dependent CVs and *C_s_* is the specific capacitance of the catalysts per unit area under identical electrolyte [217]. A higher ECSA often correlates with better catalytic activity, as it exposes a greater number of accessible reactive sites. However, determining ECSA remains a challenge, particularly for precious metal‐free catalysts, as no universal method exists. Common approaches include underpotential deposition (Cu or Pb), CO stripping, redox peak integration, double‐layer capacitance measurements, and EIS [218, 219, 220, 221, 222]. Moreover, each method has its own limitations, requiring careful selection and experimental validation. Given the complexity of measuring ECSA, explicit description of the methodologies could contextualize electrocatalyst activity and active site distribution.

#### 5.2.3 Mass Activity

Mass activity is defined as the current normalized to the catalyst mass (A g^−1^) at a given overpotential, as follows [217]:

(8) $$j_{mass\;activity}=\frac{j_{geo}\times A_{geo}}{m_{noble\;metal}}$$

where *j_geo_* is the geometric current density, *A_geo_* is the geometric area, and *m*_*noble* *metal*_ is the calculated mass of noble metal loaded onto the electrode. Mass activity is commonly reported for noble‐metal‐based catalysts, and its accurate determination is crucial for assessing intrinsic catalytic properties. The normalization of catalytic activity to the mass of active material enables assessing catalyst utilization efficiency and optimizing material loading. High mass activity is desirable for reducing material costs without compromising performance. Measuring catalyst mass is straightforward for RDEs with drop‐casted catalysts when the ink composition and deposition parameters are reported. However, it is relatively more challenging for self‐supported catalysts or those grown on conductive substrates. In such cases, catalyst mass is quantified via mass balance methods or proper characterization techniques, including energy dispersive X‐ray spectroscopy (EDS) [43], X‐ray fluorescence (XRF) [223], and inductively coupled plasma mass spectrometry (ICP‐MS) [224].

#### 5.2.4 Tafel Slope

Tafel slope (b) quantifies the relationship between the overpotential (η) and the logarithm of the current density (logj), as follows [225]:

(9) η=a+blogj

It serves as a key descriptor of reaction kinetics, offering insights into RDSs and mechanistic pathways [225]. A lower Tafel slope denotes to the more rapid ascent of current density with increasing applied voltage. The exchange current density (j_0_), defined as the current density for zero overpotential, represents the kinetic characteristics and intrinsic activity [226]. Tafel slope is conventionally considered independent of surface area normalization, since area normalization shifts the curve horizontally without altering the slope, with the condition that the current originates purely from Faradaic processes and the system is under true kinetic control [227, 228]. However, porous or non‐uniform electrodes may suffer from capacitive contributions, inhomogeneous current distributions, or structural effects that can distort the slope, particularly if ohmic resistance or transient artifacts remain unaccounted for [229, 230]. Tafel slope remains not always constant across different applied potentials, due to the varying surface coverage of reaction intermediates [231]. Tafel slope is susceptible to non‐kinetic contributions, including mass transport limitations, ohmic resistance variations, and capacitive currents [232]. This obscures kinetic interpretations [233]. Another challenge arises from the use of potentiodynamic techniques, i.e., LSV and CV [234]. CA and CP provide more accurate steady‐state measurements. Additionally, the gas bubble formation and ohmic drop compensation impact the accuracy of Tafel slope analysis [225, 228, 234]. Consequently, Tafel slopes derived from half‐cell measurements should be interpreted as intrinsic kinetic descriptors, and their extrapolation to full‐cell operation—particularly at high current densities—must be treated with caution due to emerging transport and interfacial limitations.

#### 5.2.5 Turnover Frequency (TOF)

TOF is defined as the number of times the catalytic reaction occurs, or the amount of target products or reactants consumed per unit time and per unit active site [235]:

(10) TOF=jA/zFn

where j is the current density at a specific applied voltage, A is the electrode working area, z is the electron transfer number per molecule, F is the Faraday constant, and n is the mole number of active sites on the electrode. Accurate TOF determination requires the knowledge of catalyst structure, surface composition, and active site density. For crystalline materials, crystallographic data, catalyst dispersion, and atomic site calculations must also be considered [236]. The total number of exposed active sites is estimated based on the catalyst loading and surface area. Explicit description of estimations, assumptions, and methodologies in the calculation of TOF will be critical to ensuring accuracy and reproducibility.

#### 5.2.6 Electrochemical Impedance Spectroscopy (EIS)

EIS evaluates the catalyst impedance as a function of frequency [237]. It provides in‐depth information on the process dynamics and evolution at the electrolyte/catalyst interface. EIS discloses the nature of reaction mechanisms. It provides insights into the control step of the reaction by clarifying whether it is the charge transfer, substance diffusion, or chemical reaction. EIS allows for predicting the diffusion coefficient when combined with appropriate modeling and experimental setups [238]. EIS can also elucidate the electrode interface, interfacial kinetics, and OER mechanisms [239].

#### 5.2.7 Faraday Efficiency (FE)

FE describes the efficiency of electrons catalyzing the OER [240]. FE quantifies the molar ratio between the hydrogen/oxygen generated and the theoretical amount of hydrogen/oxygen. FE measurements also provide a means to identify side reactions.

### 5.3 Thermodynamics

OER, also known as water oxidation, is a fundamental half‐cell reaction in water splitting. OER produces oxygen, protons, and electrons through oxidation of water molecules [241]. Acidic‐media OER proceeds as follows [200].

(11) $$2H_{2}O\left(l\right)\to O_{2}\left(g\right)+4H^{+}+4e^{-}\;E^{0}=1.23\;V\;vs.\;SHE$$

**FIGURE 4:**
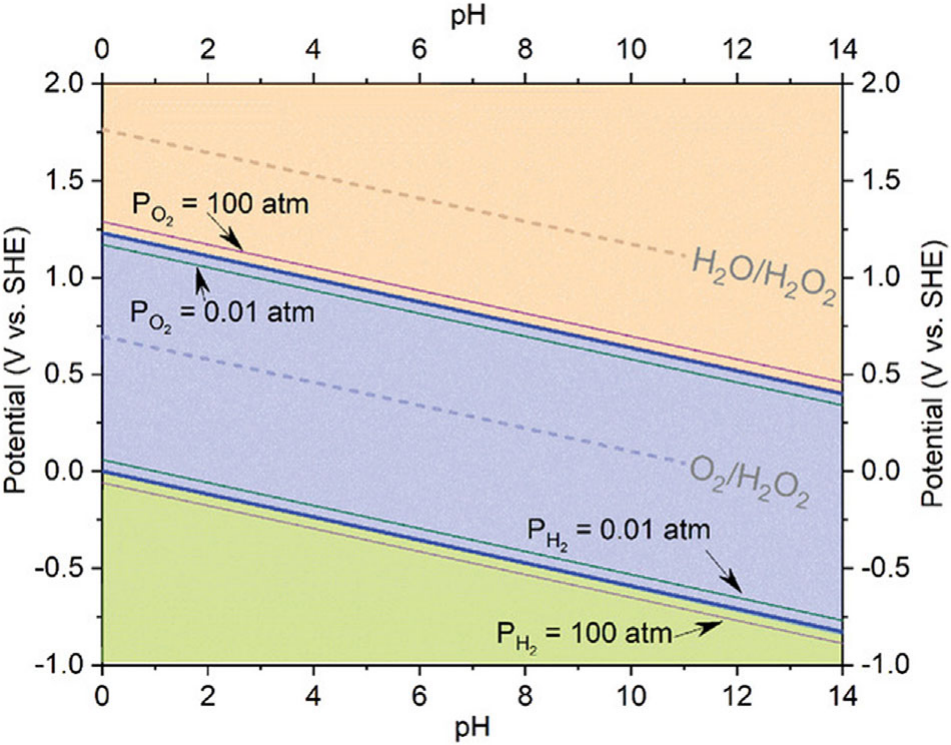
Oxygen, water, and hydrogen equilibrium potential‐pH diagram. Reproduced with permission [245]. Copyright 2021, John Wiley & Sons.

The equilibrium potential of the reactions varies with oxygen pressure and electrolyte pH (Figure 4), and it can be determined by the Nernst equation [242]. The reaction medium affects the thermodynamic equilibrium and kinetic barriers [243]. The relationship between pH and OER activity follows a specific reaction order [244]. Zero‐order kinetics indicate that the reaction rate is independent of proton concentration. First‐order kinetics suggest a linear dependence on proton concentration. The efficiency of proton exchange between the electrolyte and active sites is influenced by the pKa of the active site relative to the electrolyte pH. Active sites with low pKa (high acidity) favor fast deprotonation, enabling rapid reaction kinetics. Conversely, the active sites with high pKa (low acidity) and low oxidation potential facilitate electron transfer, forming positively charged intermediates and driving the OER [84].

Beyond pH, the electrolyte identity and composition influences OER kinetics and stability. The cations and anions in the electrolyte impact the electrocatalytic performance. Acidic‐media OER is typically performed using sulfuric acid (H_2_SO_4_) and perchloric acid (HClO_4_). H_2_SO_4_ shows structural similarity with the sulfonated groups in perfluorosulfonic acid‐based PEMs. HClO_4_ possesses perchlorate anions that are weakly adsorbed on the catalyst surface, reducing the interference with OER [246, 247]. The adsorption of anions on the catalyst surface could enhance or deteriorate OER activity. HClO_4_ electrolytes generally enable higher catalytic performance than H_2_SO_4_ for certain catalyst types [248], i.e., Ir‐black nanoparticles. Additionally, the concentration of HClO_4_ (0.05 m vs. 0.1 m) possesses a minimal effect on OER, suggesting that the electrolyte identity might play a more significant role than its concentration in catalytic efficiency [249].

## 6 Acidic‐Media OER Catalysts for CO_2_ Electrolyzers

Acidic‐media OER catalysts constitute the anodic backbone of PEMWEs and acidic‐media CO_2_ electrolyzers. In both systems, the OER dictates overall energy efficiency and cell durability. However, the anodic environments show certain differences. In PEMWEs, the OER occurs in a low‐pH environment where degradation is governed mainly by metal dissolution and structural reconstruction. In acidic‐media CO_2_ electrolyzers, the anode is typically exposed to pH fluctuations, due to the spontaneous reactions between protons (originating from OER) and OH^–^ and (bi)carbonate ions at the cathode. These additional variables could alter catalyst stability and interfacial chemistry, influencing the entire cell's long‐term performance.

Catalysts that perform well in PEMWEs might not automatically be assumed to function effectively in CO_2_R systems. In acidic‐media CO_2_R systems, the dissolved metal cations and volatile oxides from the anode can migrate across the membrane and deposit on the cathode, poisoning CO_2_R catalysts and reducing FE toward CO_2_R products. Consequently, OER catalysts for acidic‐media CO_2_R systems must meet more stringent criteria: not only high activity and intrinsic acid stability, but also chemical inertness and a higher degree of resistance to pH fluctuations. Evaluating catalysts under isolated half‐cell tests provides only partial insights; therefore, full‐cell validation within realistic CO_2_R configurations is essential for accurate assessment of activity, stability, and ion transport behavior.

Recent studies have clarified that the anodic environment of acidic‐media CO_2_ electrolyzers differs profoundly from that of PEMWEs. In addition to low‐pH oxygen evolution, the anode could be simultaneously exposed to CO_2_ and its derivatives. Fan et al. [33] and Huang et al. [15] demonstrated that even trace amounts of (bi)carbonate regeneration occurring at the anode rather than the cathode can alter the local pH near Ir‐based anodes and accelerate Ti substrate oxidation, leading to voltage drift within several tens of operating hours. Yu et al. [250] further confirmed that at pH ≈ 1, IrO_2_ anodes sustain 300 h of operation but remain the dominant source of energy loss, accounting for >25% of total cell voltage. These results highlight why the OER side dictates the long‐term durability of acidic‐media CO_2_R systems and justify focusing this section on acid‐stable OER catalysts.

**FIGURE 5:**
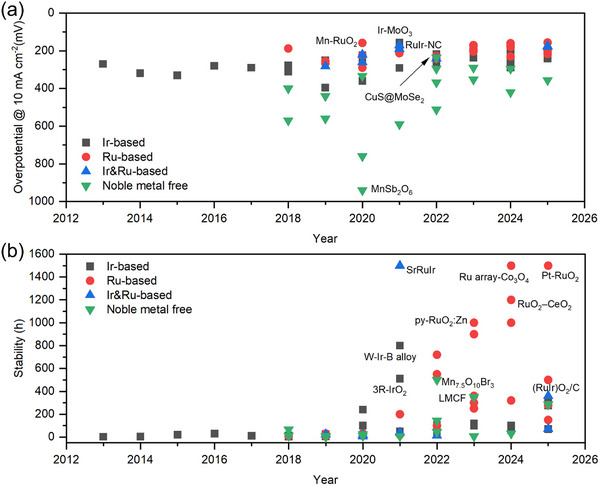
Progress in performance and practicality indicators of acidic‐media OER. (a) The overpotentials of acidic‐media OER catalysts at a constant current density of 10 mA cm^−2^ (η_10_). (b) Operational stability of acidic‐media OER catalysts. Note: Reported overpotential and stability data are collected from independent studies using different electrolyzer architectures, electrolyte compositions, and operating parameters. Therefore, these results should be interpreted as indicative rather than strictly comparable benchmarks. The stability durations represent reported operation periods and may not correspond to the intrinsic maximum durability of each catalyst, as some tests were terminated by experimental constraints rather than catalyst failure.

Figure 5a,b summarizes the progress of OER catalysts operating in acidic media in terms of intrinsic performance and practical viability. Among these, Ir‐ and Ru‐based catalysts remain the most active and durable systems reported to date, serving as benchmarks for acidic environments. Nevertheless, each class of catalyst presents a distinct balance between activity, stability, and cost. Identifying materials that combine acid stability with scalable synthesis and economic feasibility is therefore central to advancing practical acidic‐media CO_2_R systems. The following discussion examines the state‐of‐the‐art noble‐metal, non‐noble, and hybrid OER catalysts, highlighting their structural features, degradation behaviors, and emerging strategies to reconcile performance with sustainability. Acidic‐media OER catalysts reported to date have been evaluated primarily using three‐electrode half‐cell configurations. However, it should be emphasized that the performance and stability data summarized in Figure 5 are derived from studies employing substantially different testing protocols. These variations include electrolyte composition (e.g., 0.5 m H_2_SO_4_ vs 0.1 m HClO_4_), catalyst loading (ranging from tens of µg cm^−^^2^ for Ir‐based catalysts to several mg cm^−^^2^ for non‐noble systems), and stability evaluation criteria (e.g., operation at 10 or 100 mA cm^−^^2^), which together preclude direct quantitative comparison across studies.

### 6.1 Noble‐Metal‐Based OER Catalysts

#### 6.1.1 Ir‐Based Catalysts

Ir‐based oxides remain the benchmark for acidic‐media OER because of their superior intrinsic activity and corrosion resistance under highly oxidative environments. Table 2 summarizes the specifications and performance metrics of state‐of‐the‐art Ir‐based OER catalysts. Despite showing substantial performance and stability advances, practical applications demand further improvements in activity, durability, and Ir utilization efficiency through structure and composition engineering.

**TABLE 2:** Performance of benchmark acidic‐media OER catalysts.

Catalyst	η_10_ (mV)	Tafel slope (mV dec^−^^1^)	Mass loading (mg cm^−^^2^)	Electrolyte	S number	Stability (hours @ mA cm^−^^2^)	Cell performance (V @ A cm^−^^2^)	Cell stability (hours @ A cm^−^^2^)	Refs.
Ta_0.1_Ru_0.9_O_2‐x_	226	47.1	0.25	0.5 m H_2_SO_4_	∼10^5^	—	1.704 @ 1	2800 @ 1	[37]
M‐RuIrFeCoNiO_2_	189	49	0.476	0.5 m H_2_SO_4_	5.7 × 10^4^	120 @ 10	—	500 @ 1	[251]
IrO*_x_*/t‐ZrO_2_	287.5	51.5	0.1	0.1 m HClO_4_	3.6 × 10^6^	1000 @ 10	1.9 @ 3.1	1600 @ 1	[70]
RIE‐Ir/CeO_x_	—	—	—	—	—	—	1.49 @ 1 1.62 @ 2 1.72 @ 3	6000 @ 2	[36]
Ir‐RuO_2_	167	48.2	1	0.5 m H_2_SO_4_	2 × 10^4^	1023 @10 255 @ 100	1.6 @ 1 1.8 @ 2	300 @ 1	[252]
Ir/TiN	277	43	1.5	0.5 m H_2_SO_4_	3.7 × 10^5^	1000 @ 10	1.6 @ 1 1.8 @ 2	500 @ 1	[253]
Ir−Mn IMC	—	38.2	0.255	0.1 m HClO_4_	2.8 × 10^5^	—	1.851 @ 3	2160 @ 2	[64]
RuIrFeCoCrO_2_	185	41	1	0.5 m H_2_SO_4_	—	1000 @ 10	—	600 @ 1	[93]
Se‐RuOx	188	64.6	—	0.5 m H_2_SO_4_	2.2 × 10^5^	150 @ 100	1.67 @ 1	1000 @ 1	[65]
RuO_2_/LiCoO_2_	150	51.97	1	0.5 m H_2_SO_4_	—	2300 @ 10	1.68 @ 1	2000 @ 1	[254]
Nb_0.1_Mn_0.1_Ru_0.8_O_2_	207	55.3	0.4	0.5 m H_2_SO_4_	2.27 × 10^6^	200 @ 10	1.7* @ 1	1000 @ 0.5	[73]
Cr_0.2_Ru_0.8_O_2‐x_	170	75.3	0.5	0.1 m HClO_4_	1.1 × 10^6^	2000 @ 10	1.77 @1	200 @ 1	[255]
RuO_x_‐nanoarray‐400	—	47.7	0.22	0.5 m H_2_SO_4_	—	200 @ 1000	1.88 @ 2	500 @ 1	[256]
Pt–RuO_2_	215	63.89	0.88*	0.5 m H_2_SO_4_	—	1500 @ 10	1.567 @ 1 1.673 @ 2 1.791 @ 3	500 @ 0.5	[257]
SnRuO_x_	194	38.2	0.04	0.5 m H_2_SO_4_	1.04 × 10^6^	250 @ 100	1.565 @ 1 1.655 @ 2 1.735 @ 3	1300 @ 1	[258]
LMCF	353	60*	0.26	0.1 m HClO_4_	N/A	350 @ 10	3 @ 4	100 @ 0.2	[259]
HfS_0.52_O_1.09_	295	64	—	0.5 m H_2_SO_4_	N/A	24 @ 50	2 @ 1.072*	120 @ 0.02	[260]
Mn_7.5_O_10_Br_3_	295	68	—	0.5 m H_2_SO_4_	N/A	500 @ 10	1.75 @ 0.1*	300 @ 0.1	[261]
CoFeNiMoWTe	373	66.8	1.04	0.5 m H_2_SO_4_	N/A	100 @ 10	1.81 @ 1	100 @ 1	[262]

*Note*: The symbol * indicates data extracted from reference datasets or graphical results, while — denotes information that is not disclosed in the referenced sources. N/A indicates that the S‐number is not applicable for noble‐metal‐free catalysts.

Crystalline rutile IrO_2_ features robust Ir^4^^+^─O bonding that ensures excellent stability but limits surface reactivity, resulting in high overpotentials. In contrast, amorphous IrO_x_ exhibits superior activity owing to its flexible Ir─O coordination and abundant active sites [89, 263, 264]. The coexistence of Ir(III) species facilitates subsurface deprotonation and electron doping, lowering the reaction barrier but accelerating dissolution. The controlled calcination, by converting Ir(III) to Ir(IV), restores durability, confirming that crystallinity governs the trade‐off between activity and stability [265]. Nanostructuring provides an additional means to improve Ir utilization. More specifically, nanowires, mesoporous Ir, and nanoporous IrO_2_ architectures increase the ECSA and optimize intermediate adsorption, thereby achieving high OER activity at low Ir loadings [266, 267, 268, 269].

Alloying Ir with transition metals offers an effective route to tailor the electronic structure and reduce Ir consumption. Incorporation of Ru, Ni, or Co modifies the d‐band center and adsorption strength of oxygen intermediates, accelerating the kinetics while reshaping the activity–stability balance [270]. Ir–Ru alloys are among the most studied systems for their outstanding activity [271]. However, Ru tends to dissolve under anodic potentials, forming volatile RuO_4_ and limiting long‐term durability [272]. Ir–Ni and Ir–Co alloys enhance activity through electronic redistribution but require suppression of 3d‐metal leaching to sustain acid stability [273, 274, 275]. Complex oxides, such as Ir‐based perovskites, double perovskites, and pyrochlores, reduce Ir usage without compromising catalytic performance [276, 277, 278]. Heteroatom doping (e.g., Sn, Ti, and W) and defect engineering further modulate Ir–O covalency and local electronic density, suppressing over‐oxidation and improving activity and corrosion resistance [279, 280, 281, 282].

High‐entropy alloys (HEAs) and HEOs introduce severe lattice distortion and multi‐metal coupling that generate defect‐rich active centers [283]. The “cocktail effect” enables simultaneous enhancement of activity and stability [284]. HEAs synthesized through liquid‐metal or thermal‐shock routes exhibit high mass activity and smaller Tafel slopes than monometallic Ir catalysts [285, 286, 287, 288]. For instance, IrFeCoNiCu‐HEA nanoparticles show superior performance but undergo selective dissolution of Fe, Co, Ni, and Cu, forming Ir‐enriched surface layers during acidic‐media electrolysis [289]. Mn incorporation in ZnNiCoIrMn HEA downshifts the Ir d‐band center and promotes OER kinetics [290]. Likewise, the HEO RuIrFeCoCrO_2_ prepared via naked‐ion assembly operates through the adsorbate evolution mechanism, reaching an η_10_ of 185 mV and maintaining 1000 h of stability, while sustaining 1 A cm^−^^2^ for 600 h in a PEMWE [93].

Supported Ir‐based catalysts achieve improved Ir utilization and corrosion resistance through strong metal–support interactions (SMSIs) [291, 292]. Conductive and chemically stable supports, such as TiO_2_, antimony‐doped tin oxide (ATO), and Ta_2_O_5_ anchor Ir nanoparticles, prevent agglomeration and reduce dissolution under acidic conditions [293, 294, 295, 296]. These supported catalysts exhibit promising full‐cell OER performance, including a cell voltage of 1.9 V, a current density of 2 A cm^−^^2^, and stability of 200 h [297]. Tin (Sn)‐based oxides also provide robust stability; fluorine‐doped SnO_2_ (FTO) maintains structural integrity up to 2.7 V vs. RHE, outperforming other Sn‐based supports [298]. Conductive oxides thus enable uniform Ir dispersion and protect active sites from oxidative degradation.

SACs represent the most atom‐efficient Ir configurations, in which isolated Ir atoms are stabilized on strong supports, such as TiC, graphene, or WO_3_ [299, 300]. Atomic dispersion maximizes site exposure and enables charge‐transfer‐induced modulation of Ir oxidation states, fine‐tuning intermediate adsorption energies and accelerating OER kinetics [301, 302]. Experimental studies reveal a volcano‐type relationship between Ir loading and activity, with optimal performance obtained at ∼14 wt.% Ir loading, balancing active‐site density and electronic coupling [303]. Excessive Ir loading induces charge redistribution and partial Ir reduction, decreasing intrinsic activity. These observations highlight the importance of precise control over Ir dispersion and interfacial electronic structure in achieving durable and cost‐effective acidic‐media OER catalysts.

In PEMWEs and acidic‐media CO_2_R systems, Ir‐based catalysts demonstrate industrially relevant performance, underscoring the close technological linkage and transferability between these two electrolysis platforms. Ir nanodendrites supported on ATO achieve current densities of 3 A cm^−^^2^ at ∼2.0 V with a mass activity of 339 A gIr^−^^1^ cm^−^^2^, while supported systems, such as Ir/Nb_2_O_5__−__x_ and IrO_2_/TiO_2_, sustain multi‐ampere operation for hundreds to thousands of hours [234, 235, 236, 237]. Representative multi‐metal systems, including RuIrFeCoCrO_2_ HEOs, further confirm that PEMWE‐validated anode architectures can deliver long‐term stability under acidic conditions [76].

Collectively, these results demonstrate that optimizing Ir oxidation state, dispersion, and metal‐support electronic interactions, and design principles extensively validated in PEMWEs is central to minimizing precious‐metal usage while maintaining long‐term durability, thereby providing a transferable framework for the design of anode electrodes for acidic‐media CO_2_ electrolyzers [265, 272, 291, 292].

#### 6.1.2 Ru‐Based Catalysts

Ru‐based catalysts have emerged as promising alternatives to Ir‐based systems owing to their comparable OER activity and much lower cost, as Ru costs roughly one‐fifth that of Ir [40]. RuO_2_ could deliver comparable intrinsic activity to IrO_2_, mainly due to Ru atoms with unsaturated coordination (Ru_CUS) that accelerate OER kinetics [304]. Table 2 summarizes the specifications and performance metrics of state‐of‐the‐art Ru‐based OER catalysts. Despite demonstrating promising activity, Ru‐based catalysts tend to encounter a severe stability‐limiting challenge: rapid oxidation to high‐valent RuO_4_ causes severe dissolution under acidic conditions, defining the key activity–stability trade‐off [140, 165]. Research has therefore focused on doping and structural modification to suppress Ru over‐oxidation and corrosion.

Elemental doping provides an effective strategy to stabilize Ru oxides by tuning their electronic structure. Mn‐ and Sn‐doped RuO_2_ lower the energy barrier of the RDS and improve durability by downshifting the Ru d‐band center [74, 258]. Incorporation of Ru into Mn‐based oxide frameworks initially enhances activity but often fails to prevent long‐term dissolution, motivating the development of asymmetric M–O–Ru motifs [217]. In Mn_0_._75_Ru_0_._25_O_2_, Mn–O–Ru dual sites promote radical‐coupling (I2W/OPM) pathways while stabilizing lattice oxygen, effectively mitigating Ru over‐oxidation and extending catalyst lifetime [305].

Structural engineering further balances activity and stability by decoupling active Ru sites from stabilizing components. Core–shell architectures, such as Ru@IrO_x_, exploit interfacial charge redistribution to maintain high OER activity while inhibiting Ru dissolution, enabling operation at 1 A cm^−^^2^ for over 1000 h in PEMWEs [306, 307]. Mixed‐metal oxides and pyrochlores similarly leverage multi‐metal synergy and vacancy engineering to stabilize Ru─O bonding. Metal‐organic framework (MOF)‐derived Cr_0_._6_Ru_0_._4_O_2_, Ru–Ir pyrochlores, and rare‐earth pyrochlores, such as Nd_2_Ru_2_O_7_, weaken Ru─O hybridization through electronic and structural modulation, achieving improved stability relative to commercial RuO_2_ [308, 309, 310, 311, 312]. Perovskite‐type and amorphous Ru oxides further benefit from structural flexibility, which accommodates dynamic reconstruction and delays dissolution during OER [313, 314].

Despite these advances, the intrinsic instability of Ru‐based catalysts remains closely linked to their electronic configuration. Unsaturated Ru_CUS sites enhance activity but also promote over‐oxidation to soluble RuO_4_ [304]. Stabilization strategies—including elemental doping, interfacial charge redistribution, and vacancy engineering—moderate Ru─O covalency and suppress lattice‐oxygen participation [258, 306, 312]. Nevertheless, proton penetration into the oxide lattice continues to drive structural degradation under acidic conditions. Future progress will therefore depend on restricting Ru over‐oxidation while preserving high intrinsic activity through robust interfacial protection and controlled electronic‐structure design, enabling Ru‐based materials to evolve into cost‐efficient anodes for acidic‐media CO_2_ electrolyzers.

### 6.2 Non‐Noble Metal‐Based OER Catalysts

To overcome the economic and resource constraints associated with Ir and Ru, extensive efforts have been devoted to developing non‐noble and hybrid OER catalysts for acidic‐media CO_2_ electrolyzers. These materials offer lower cost, flexible electronic structures, and compatibility with conductive or corrosion‐resistant supports. However, under the strongly acidic and highly oxidative conditions required for OER, their long‐term stability remains fundamentally limited [315]. Table 2 summarizes the specifications and performance metrics of state‐of‐the‐art non‐noble metal‐based OER catalysts. Rapid cation dissolution and acid‐induced structural degradation dominate catalyst failure, particularly when non‐noble oxides are integrated into full CO_2_R systems that experience dynamic pH fluctuations and ion crossover [15, 33].

At a fundamental level, the instability of non‐noble, 3d‐metal‐based oxides in acidic‐media OER originates from coupled thermodynamic and mechanistic constraints. Pourbaix analyses indicate that at low pHs and high anodic potentials, most 3d‐metal oxides lack stable solid‐phase regions and preferentially dissolve into soluble high‐valence cations. In addition, OER‐relevant potentials readily drive 3d metal centers into highly oxidized states, weakening metal–oxygen bonds and accelerating proton‐assisted leaching. Strategies that enhance activity—such as increasing metal–oxygen covalency or activating lattice‐oxygen redox—often intensify structural degradation by promoting oxygen vacancy formation, surface reconstruction, and framework collapse. Consequently, 3d‐metal oxides inherently suffer from a pronounced activity–stability trade‐off in acidic environments, explaining why purely non‐noble oxide catalysts remain far from industrial‐level deployment in acidic‐media CO_2_ electrolyzers, where sustained operation at industrially relevant current densities and full‐cell durability against pH fluctuations and ion crossover are required. Related 3d non‐oxide compounds also face rapid surface oxidation or dissolution under acidic‐media OER. Thus, these compounds are more often used as conductive frameworks in hybrid anodes.

**FIGURE 6:**
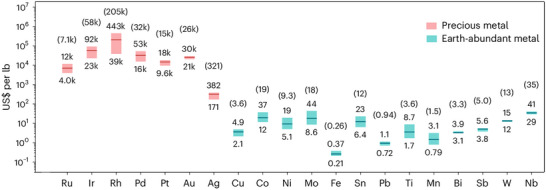
Prices of noble‐ (precious metals) and non‐noble elements (earth‐abundant metals) between January 2019 and January 2024. Each column represents the price range, with the maximum and minimum values indicated, respectively, above and below each shaded area, where k denotes ×1000. The darker line between the maximum and minimum values denotes the average price, which is given in parentheses for each metal above the maximum value. Reproduced with permission [317]. Copyright 2024, Springer Nature.

Nevertheless, insights from these systems have informed several design principles—particularly entropy‐stabilized lattices, multi‐metal synergy, and conductive hybrid frameworks—that might guide the development of acid‐tolerant anodes for acidic‐media CO_2_R [130]. Recent reviews emphasized that base‐metal oxides and hybrid frameworks, while unable to remain intact in strong acids, can act as conductive and partially protective scaffolds that electronically and structurally stabilize Ir or Ru nanophases [250, 316]. These composite architectures extend lifetime and reduce noble‐metal consumption in acidic‐media CO_2_ electrolyzers. Figure 6 illustrates the cost disparity between noble and earth‐abundant metals, highlighting the economic rationale for pursuing non‐noble or hybrid OER catalysts in acidic‐media CO_2_R [317].

Despite these intrinsic limitations, studies on non‐noble and hybrid systems have yielded valuable design principles—particularly entropy‐stabilized lattices, multi‐metal synergy, and conductive hybrid frameworks—that may partially mitigate corrosion and guide the development of acid‐tolerant anodes. Recent reviews emphasize that base‐metal oxides and hybrid frameworks, while unable to remain intact as stand‐alone catalysts in strong acids, can function as conductive and partially protective scaffolds that electronically and structurally stabilize Ir or Ru nanophases.

#### 6.2.1 Transition‐Metal Oxides (Co, Mn, Fe): Activity–Stability Limitations

Transition‐metal oxides containing Co, Mn, and Fe have long been regarded as cost‐effective OER catalysts. These catalysts also offer flexible redox chemistry and electronic tunability. Despite their promising alkaline‐media performance, these oxides are intrinsically unstable under strongly acidic conditions, rendering them incompatible with the anodic environment of acidic‐media CO_2_R [318]. Rapid cation dissolution, lattice‐oxygen redox participation, and proton penetration accelerate structural collapse, preventing sustained operation [317]. At low pHs and high anodic potentials, most 3d‐metal oxides lack stable solid‐phase regions and preferentially dissolve into soluble high‐valence cations, while OER‐relevant potentials drive metal centers into highly oxidized states, weakening metal–oxygen bonds and promoting proton‐assisted leaching. Strategies that enhance activity—such as increasing metal–oxygen covalency or activating lattice‐oxygen redox—often intensify structural degradation by inducing oxygen vacancies, surface reconstruction, and framework collapse. Consequently, 3d‐metal oxides tend to suffer from a pronounced activity–stability trade‐off in acidic environments, and no purely base‐metal oxide has yet demonstrated long‐term stability or durable activity as the anode of acidic‐media CO_2_R. Nevertheless, mechanistic studies of their instability have yielded valuable insights for stabilizing hybrid and high‐entropy anodes [124].

Co‐based oxides, such as Co_3_O_4_ and Co_2_MnO_4_, exhibit moderate OER activity but dissolve rapidly under oxidative potentials. Using operando spectroscopy, Huang et al. demonstrated that interfacial Co oxidation states vary strongly with pH, leading to sluggish Co^3^^+^/^4^^+^ redox kinetics, higher overpotentials, and progressive acidic‐media instability [319]. Simondson et al. further decoupled catalytic and degradation processes, showing that active Co(3+δ)+–oxo species coexist with continuous Co^2^^+^ leaching and that Co corrosion and catalytic turnover proceed independently, indicative of a dynamic dissolution–redeposition cycle sustaining only quasi‐steady operation [320]. Alloying Co with Fe or Mn or doping with Sb and Ba can transiently enhance electronic conductivity but fails to suppress cation dissolution under acidic conditions, reflecting weak Co─O bonding and facile proton‐assisted oxidation [315]. Fe‐based materials, such as α/γ‐Fe_2_O_3_, have also been examined for acidic‐media OER. However, Fe^3^^+^ rapidly dissolves under oxidative potentials, and only partial protection can be achieved through structural confinement or heteroatom incorporation. Co‐doped α‐Fe_2_O_3_ thin films and γ/α‐Fe_2_O_3_ mixed‐phase architectures improve activity via oxygen‐vacancy formation and lattice distortion, but operation is typically limited to tens of hours [321, 322, 323]. Embedding Fe species into conductive scaffolds, such as TiO_x_ nanowires or PbO_2_ matrices, enhances charge transfer and delays Fe leaching but cannot prevent gradual corrosion [324, 325]. These observations confirm that Co‐ and Fe‐based oxides exhibit transient activity intrinsically coupled with continuous metal dissolution.

Mn‐based oxides offer redox flexibility through multiple oxidation states (Mn^2^^+^–Mn^7^^+^), yet their intrinsic instability in acid severely limits practical use [326]. The dominant degradation pathway is the disproportionation of Mn^3^^+^ intermediates into soluble Mn^2^^+^ and insoluble Mn^4^^+^ species, causing mass loss and lattice collapse [327]. Among the polymorphs, γ‐MnO_2_ shows the highest transient stability, attributed to its tunnel structure that facilitates electron transport and delays dissolution, but sustained operation in strong acid remains challenging [318, 328]. Compositional tuning and structural confinement—such as Co─Mn spinels (Co_2_MnO_4_) or Mn─Sb─O frameworks that stabilize Mn^3^^+^—provide only short‐term benefits, while surface modifications (Ti, Si doping or oxide coatings) merely slow Mn^4^^+^ reduction without eliminating degradation [59, 329, 330, 331, 332]. Notably, Li et al. demonstrated a MnO_x_ OER system capable of maintaining activity for >2000 h at pH 2 through a built‐in self‐healing mechanism, in which the Guyard redox cycle (4 Mn^2^^+^ + Mn^7^^+^ → 5 Mn^3^^+^) enables periodic catalyst regeneration under fluctuating potentials [333]. Such dynamically regenerative anodes hold promise for acidic‐media CO_2_R systems and suggest that exploiting redox equilibria may partially overcome the stability gap of Mn‐based systems and inform the design of adaptive, long‐lived hybrid anodes.

Overall, transition‐metal oxides show encouraging intrinsic activity but lack the chemical resilience required for acidic‐media CO_2_R. Their instability arises from lattice‐oxygen redox, cation dissolution, and acid‐induced surface reconstruction, and is further aggravated by interfacial pH fluctuations and ion crossover in full CO_2_ electrolyzers [33, 59, 334]. Nevertheless, mechanistic understanding—particularly of dissolution–redeposition cycles and self‐healing Mn oxides—has provided critical insights for stabilizing hybrid, high‐entropy, and support‐coupled OER catalysts that can better reconcile activity with acid tolerance [124, 333]. As a result, despite their attractive intrinsic activity, 3d‐metal oxides remain far from practical deployment in acidic‐media OER applications due to their inherent instability under low‐pH and high‐potential conditions, which pose critical challenges for long‐term operation in acidic‐media CO_2_ electrolyzers.

#### 6.2.2 High‐Entropy and Mixed‐Metal Oxides: Emerging Acid‐Tolerant Systems

Mixed‐metal oxides (MMOs) and HEOs have emerged as promising non‐noble catalysts for acidic‐media OER and CO_2_R, where the anode must sustain high activity while resisting corrosion [335, 336]. Incorporating multiple metallic elements into a single lattice enables multi‐cation synergy that enhances electronic conductivity, optimizes intermediate adsorption, and suppresses localized dissolution [337]. Compared with single‐metal oxides that degrade rapidly in acid, multi‐metal frameworks distribute oxidative stress among several cations, providing partial resistance to corrosion. These synergistic effects, combined with the ability to tune surface energies and electronic states via elemental composition, make MMOs particularly attractive for acidic‐media OER [338].

**FIGURE 7:**
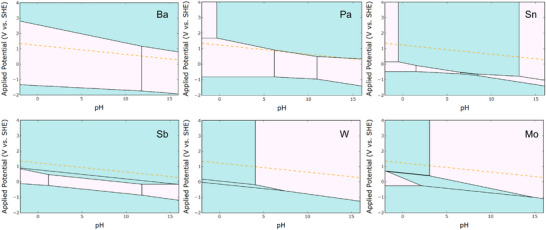
Stabilizing elements and their Pourbaix diagrams at an ion concentration of 1 × 10^−6^ m. The green‐shaded regions indicate stable solid species. Data sets are generated with the Materials Project. Reproduced with permission [347]. Copyright 2012, American Physical Society.

Representative MMOs, including Co–Fe, Co–Mn, and Mn–Sb systems, exemplify the benefits of elemental cooperation. In these materials, one component typically contributes OER activity, while others stabilize the lattice through stronger metal–oxygen bonding and improved charge transport [336, 337, 339]. Incorporation of stabilizing elements (e.g., Ba, Pb, and Sb) further expands the electrochemical stability window by forming acid‐resistant oxide layers near the membrane interface, as illustrated by the Pourbaix stability regions in Figure 7 [340, 341, 342]. Nevertheless, selective dissolution of less noble elements remains unavoidable, leading to gradual compositional drift during prolonged acidic‐media OER. Advanced synthesis approaches—including solvothermal methods, atomic‐layer deposition, and electrochemical reconstruction—improve cation dispersion and enable partially self‐healing surfaces, but long‐term durability in strong acid remains limited [343, 344, 345, 346].

HEOs extend the concept of local multi‐metal synergy to global lattice stabilization by integrating five or more metallic elements into a single‐phase structure [261, 348]. Increased configurational entropy stabilizes the crystal lattice and mitigates metal dissolution by distributing oxidation stress among multiple cations [226, 341, 342]. HEOs containing predominantly transition metals with trace noble‐metal substitution can achieve measurable acidic‐media OER activity while significantly reducing noble‐metal loading [262]. In particular, minor Ir or Ru incorporation (<5 at.%) sustains activity while lowering precious‐metal content by more than 80% [130, 251]. The homogeneous distribution of multiple cations redistributes redox charge and minimizes selective leaching, thereby preserving lattice integrity more effectively than binary or ternary oxides [349, 350].

Despite these advantages, entropy‐stabilized oxides are not immune to acid‐induced degradation. Achieving phase‐pure HEOs with uniform elemental distribution typically requires high‐temperature synthesis, which limits scalability and may introduce compositional inhomogeneity that accelerates corrosion [124, 351]. Moreover, durability demonstrations generally remain limited to hundreds of hours and moderate current densities, falling short of industrial requirements. Consequently, MMOs and HEOs should be viewed not as stand‐alone replacements for noble‐metal anodes, but as platforms that provide valuable design insights—such as stress distribution, cooperative bonding, and partial self‐stabilization—that can be integrated with conductive supports or thin noble‐metal layers to advance acid‐tolerant anode architectures [130, 349].

#### 6.2.3 Alternative Non‐Oxide Compounds: Phosphides, Sulfides, Carbides, Nitrides, and Borides

Conductive non‐oxide compounds, including metal phosphides, sulfides, carbides, nitrides, and borides, have attracted interest as potential alternatives to noble‐metal oxides for acidic‐media OER. These materials typically exhibit metallic bonding, high electrical conductivity, and structural tunability [338]. These attributes render non‐oxide materials conceptually appealing for acidic‐media CO_2_ electrolyzers; however, none has demonstrated sustained catalytic activity as a primary OER phase under strongly oxidative and corrosive acidic conditions. Most non‐oxide catalysts undergo surface oxidation, phase transformation, or dissolution, which converts the active phase into insulating oxides and leads to rapid performance decay [352]. Consequently, their role in acidic‐media OER has increasingly shifted away from stand‐alone catalysts toward conductive or structural components in hybrid anodes.

Metal phosphides and sulfides exemplify this limitation. Although strong metal–non‐metal bonding and high intrinsic conductivity can deliver appreciable initial activity, these materials readily oxidize to metal oxyhydroxides or oxides under OER potentials, accelerating corrosion and cation leaching [353, 354, 355, 356, 357]. Compositional tuning or composite formation may delay degradation, but long‐term stability in acidic environments remains elusive. Similarly, transition‐metal carbides and nitrides combine metallic conductivity with mechanical robustness and exhibit comparatively higher acid resistance; nevertheless, electrochemical oxidation leads to the formation of insulating surface oxides (e.g., TiO_x_ or WO_x_), which impedes charge transport and ultimately limits durability [358, 359, 360]. Metal borides, such as Ni_3_B, also display strong covalent bonding and high conductivity, yet rapid oxidation and boron leaching under acidic conditions prevent their sustained operation as active OER catalysts [361]. Despite their intrinsic instability as catalytic phases, non‐oxide materials provide significant advantages as conductive and corrosion‐resistant scaffolds. Hybrid anodes, such as Ir/TiN and Ru/TiN, leverage the nitride backbone to ensure electronic continuity and structural integrity, while thin noble‐metal layers supply OER activity [362, 363, 364]. Likewise, carbides and borides (e.g., WC and Ni_3_B) have been employed as robust supports for anchoring Ir nanoparticles, enabling reduced noble‐metal loading without sacrificing stability [346, 365]. These examples underscore that the primary value of non‐oxide compounds in acidic‐media CO_2_ electrolyzers lies not in replacing noble‐metal oxides, but in serving as electronically conductive frameworks that stabilize active phases and mitigate corrosion.

Overall, while non‐oxide compounds are fundamentally unsuitable as stand‐alone OER catalysts in acidic environments, their combination of high conductivity, mechanical robustness, and interfacial compatibility makes them well suited as structural and electronic scaffolds in hybrid anode architectures. Future advances are therefore likely to emerge from integrating non‐oxide frameworks with entropy‐stabilized oxides or ultrathin noble‐metal layers, enabling durable, low‐Ir anode designs for acidic‐media CO_2_R systems.

### 6.3 Summary and Comparison between PEMWE and Acidic‐Media CO_2_R

Compared with PEMWE, acidic‐media CO_2_R encounters additional challenges. For instance, CO_2_ regeneration at the cathode and ensuing fluctuating proton activity at the anode accelerate metal dissolution and interfacial corrosion, drastically shortening anode and system lifetimes. While PEMWEs suffer well‐defined Ir or Ru degradation routes, acidic‐media CO_2_ electrolyzers require full‐cell evaluation to capture coupled processes, such as ion crossover and product selectivity loss. Today, IrO_2_ and RuO_2_ remain the activity benchmarks, but sustainable operation demands their integration with multi‐metal lattices or conductive non‐oxide frameworks. The field thus shifts from composition‐driven discovery toward interfacial and structural engineering of acid‐tolerant OER catalysts, as further discussed in Sections 7 and 12.

## 7 Degradation and Stability Issues

This section focuses on the stability evaluation and degradation mechanisms of OER catalysts under acidic‐media CO_2_R conditions. Stability has emerged as the principal challenge that limits the industrial viability of acidic‐media CO_2_ electrolyzers. Although noble‐metal catalysts, such as Ir and Ru, exhibit promising initial activity, their dissolution and cross‐membrane migration under the dynamic micro‐reaction environment of acidic‐media CO_2_R systems typically cause cathode contamination and performance loss. Non‐noble and hybrid materials encounter even more severe corrosion in acidic environments, rendering stability as the decisive factor in system design. Importantly, catalyst stability in acidic‐media OER is not an intrinsic material constant but strongly depends on the applied current‐density regime. Degradation pathways that appear negligible under low‐current half‐cell testing (<10 mA cm^−^^2^) can become dominant under device‐relevant current densities (>200 mA cm^−^^2^), where elevated anodic potentials, intensified gas evolution, and coupled transport phenomena prevail.

This section systematically addresses three key aspects of catalyst stability: (i) the metrics and indicators used to evaluate catalyst degradation, (ii) the test protocols for accelerated and realistic assessment, and (iii) the mechanistic understanding of degradation pathways and their mitigation. Together, these insights provide a foundation for developing reliable, durable anodes for acidic‐media CO_2_ electrolyzers.

### 7.1 Stability Test and Metrics

Stability evaluation of OER catalysts is primarily conducted using electrochemical methods, such as CA, CP, and CV analyses. These techniques assess the catalyst's ability to retain its initial activity and selectivity over time. In CA, the catalyst is held at a constant potential while monitoring current variations, whereas CP involves applying a constant current and recording changes in potential. Both methods probe catalyst degradation under steady‐state operation, and small fluctuations in current or potential are typically interpreted as indicators of stable performance. CV cycling, by contrast, provides insights into the structural and chemical reversibility of active sites through potential‐induced reconstruction [365].

To quantify degradation, the S‐number—defined as the moles of oxygen evolved per mole of dissolved catalyst—has become a widely accepted metric [109].

(12) $$S=\frac{Moles\;of\;O_{2}}{Moles\;of\;catalyst\;dissolved}$$

A higher S‐number reflects greater dissolution resistance and long‐term durability. Although this parameter offers a convenient normalization method, it assumes steady‐state electrolysis in pure acidic electrolyte and therefore fails to represent the dynamic environment of acidic‐media CO_2_ electrolyzers. For example, under realistic conditions, the consumption of protons during the regeneration of CO_2_ (via the reaction between (bi)carbonate ions and protons) causes fluctuations in the acidity of the micro‐reaction environment, which alters dissolution kinetics and renders direct S‐number comparison unreliable [366, 367].

Overall, existing stability diagnosis tools, such as CA, CP, CV, and S‐number, provide useful benchmarks for evaluating catalyst robustness in acidic‐media OER [367]. However, these tools fail to provide insights into the contextual relevance [366]. In particular, stability metrics derived under low‐current conditions do not capture degradation processes activated by high current densities, such as accelerated metal dissolution, oxygen bubble–induced mechanical stress, and enhanced ion migration across the membrane. Future work should thus combine in situ metal‐dissolution tracking (e.g., online ICP‐MS), ion‐crossover quantification, and FE retention to develop unified stability descriptors applicable to acidic‐media CO_2_R systems [22, 56, 368].

### 7.2 Stability Test Protocols

**FIGURE 8:**
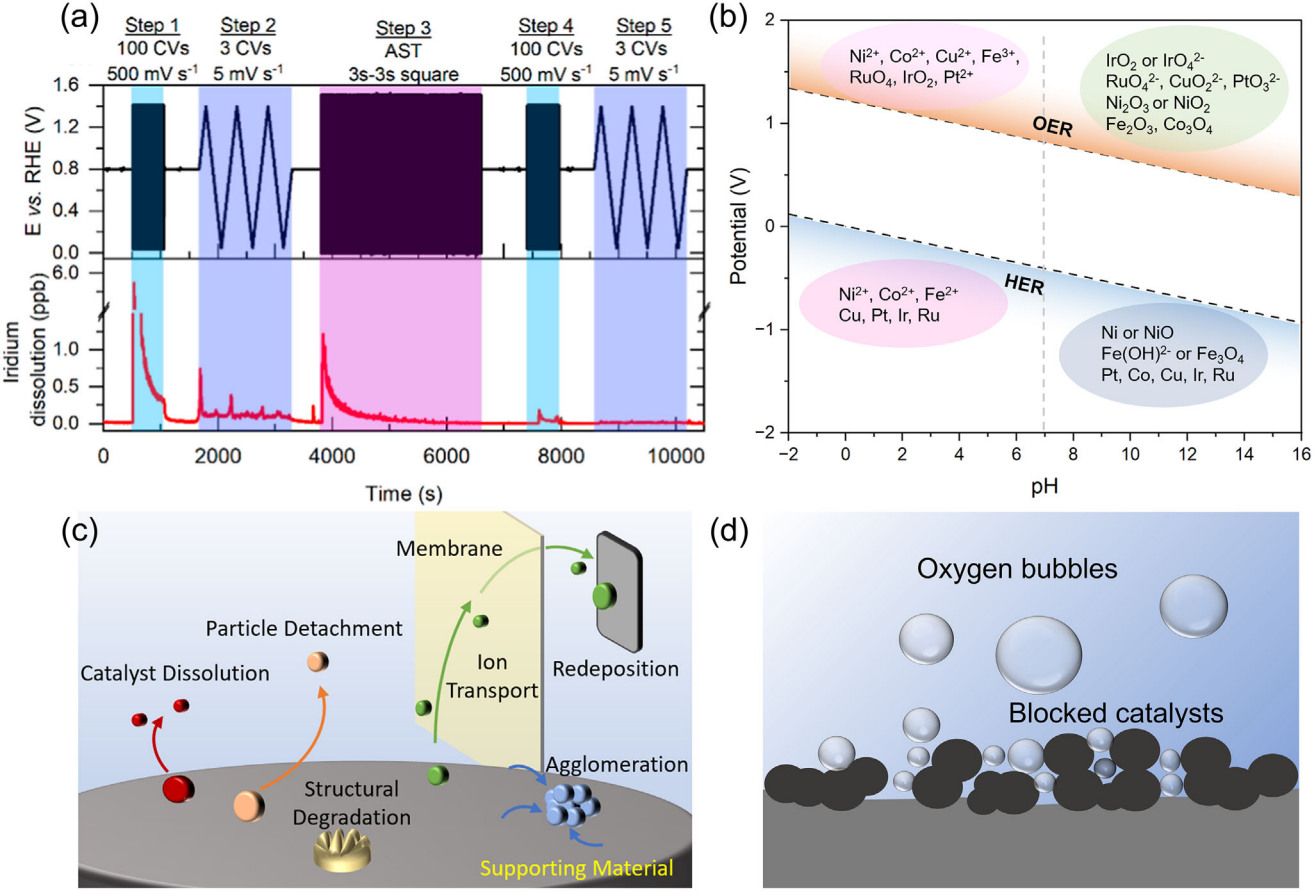
Stability challenges of acidic‐media OER catalysts. (a) AST and dissolution profiles of an OER catalyst (Ir). Reproduced with permission [265]. Copyright 2025, American Chemistry Society. (b) Pourbaix diagram of various OER catalysts. Reproduced with permission [371]. Copyright 2020, John Wiley & Sons. (c) Common degradation mechanisms for OER catalysts immobilized on a support material. (d) Formation and accumulation of microscopic oxygen bubbles in the micro‐reaction environment shield active sites.

Evaluating catalyst stability commonly relies on accelerated stress tests (ASTs) or accelerated degradation tests (ADTs), which expose materials to controlled electrochemical and environmental stressors to simulate long‐term operation within a compressed time frame. These protocols are indispensable for probing dissolution behavior, identifying failure modes, and comparing catalyst robustness under standardized conditions. Typically, an AST involves cyclic potential or current operation in the moderate anodic range (∼1.4–1.8 V vs. RHE) or at current densities up to several A cm^−^^2^ to accelerate degradation phenomena, such as metal dissolution, oxide restructuring, or particle agglomeration [369, 370]. Complementary CA or CP tests are often conducted under steady‐state conditions to provide a reference for sluggish degradation kinetics [63]. Figure 8a presents representative AST profiles for Ir‐based catalysts, showing how dissolution rates and current transients correlate with the extent of structural degradation [265].

In conventional PEMWEs, these protocols are widely used to assess catalyst endurance. Voltage or current cycling mimics start‐up and shut‐down events, while temperature and humidity variations emulate operational dynamics or intermittent renewable‐electricity inputs [372, 373]. During these tests, EIS is often employed to monitor the changes in charge‐transfer resistance, double‐layer capacitance, and overall cell impedance. These parameters are typically indicative of interfacial degradation [374, 375]. Post‐mortem characterization, using X‐ray photoelectron spectroscopy (XPS), X‐ray diffraction spectroscopy (XRD), scanning electron microscopy (SEM), or TEM, provides complementary insights into phase transformation and particle evolution [376]. Through the combination of accelerated testing and structural analysis, ASTs and ADTs have become the basis for reproducible benchmarking of catalyst stability across laboratories [377, 378].

However, these well‐established PEMWE‐oriented protocols fail to adequately represent the operating environment of acidic‐media CO_2_ electrolyzers. The fluctuating local acidity at the anode (due to the spontaneous reaction between protons and (bi)carbonate/OH^−^ ions at the cathode) and cross‐electrode gas interactions alter the degradation dynamics considerably [366, 379]. Standard AST procedures—designed for PEMWEs—might not completely reproduce the chemical buffering, membrane hydration, or gas crossover that govern corrosion and metal‐ion migration in acidic‐media CO_2_ electrolyzers [376, 380]. Consequently, results obtained under conventional AST conditions may underestimate dissolution rates or fail to predict catalyst lifetime in acidic‐media CO_2_R systems [367].

To address these discrepancies, customized durability protocols must be developed. Such tests should explicitly specify (i) the gas composition and humidity level, (ii) electrolyte pH and buffering species, and (iii) the total charge passed or oxygen evolved per cycle. Real‐time analysis techniques, such as online ICP‐MS or electrochemical quartz‐crystal microbalance (EQCM), should be integrated to quantify metal dissolution and ion crossover dynamically. Furthermore, correlating dissolution data with FE retention and membrane performance will enable more accurate prediction of full‐cell durability. Establishing a standardized testing framework for acidic‐media CO_2_ electrolyzers—through coordinated academic and industrial efforts—will be essential to ensuring consistent benchmarking and translating half‐cell catalyst evaluations into meaningful device‐level performance comparisons [381, 382]. Accordingly, a hierarchical stability evaluation framework—combining low‐current intrinsic screening with high‐current and device‐proximal durability testing—is essential for reliably assessing catalyst lifetime in acidic‐media CO_2_ electrolyzers.

### 7.3 Degradation Mechanisms

Catalyst degradation in acidic‐media CO_2_R systems originates from the combined effects of low pH, spontaneous local pH fluctuations, and high potential, creating a chemically dynamic environment that is far more aggressive than that of conventional PEMWEs.

From a mechanistic perspective, these degradation pathways can be broadly categorized into (i) intrinsic catalyst degradation, which is inherent to the catalyst material itself, and (ii) system‐induced degradation, which arises from the dynamic operating environment and cross‐component interactions in acidic‐media CO_2_ electrolyzers. In practice, the apparent performance decay of the anode often reflects a convolution of these two effects. The major degradation pathways include metal dissolution, structural degradation, nanoparticle agglomeration and detachment, and cross‐membrane ion migration and redeposition (Figure 8c) [109, 314, 383]. The relative contribution of each pathway depends on catalyst composition, morphology, and operating parameters.

#### 7.3.1 Metal Dissolution

Metal dissolution is the most direct and irreversible intrinsic degradation process. Even noble metals, such as Ir and Ru, oxidize into soluble high‐valence species (IrO_4_^2^^−^, RuO_4_) under anodic potentials, consistent with the predictions from Pourbaix thermodynamic diagrams [384, 385]. Such soluble species are particularly favored in amorphous hydrous IrO_x_, where LOM dissolution and high defect density promote cation release [386]. Metal dissolution can occur via various pathways: chemical dissolution, oxidative dissolution, and LOM dissolution [317].

Chemical dissolution:

(13) $$MO_{x}+2xH^{+}\to M_{aq}^{2x+}+xH_{2}O$$

Oxidative dissolution:

(14) $$MO_{x}+yH_{2}O\to MO_{\left(x+y\right),\;aq}^{2\left(y-x\right)-}+2yH^{+}+2xe^{-}$$

LOM dissolution:

(15) $$MO_{x}{\left(\mathrm{OH}\right)}_{y}\to M_{\mathrm{solid}}^{2x+}+\frac{x+y}{2}O_{2}+yH^{+}+\left(2x+y\right)e^{-}$$

Operando ICP‐MS and electrochemical mass spectrometry detect transient Ir^n^^+^ release that diminishes as surface sites re‐oxidize to Ir^4^^+^, indicating partial self‐passivation [22, 367]. This “kinetic stabilization” explains the reduced dissolution rate after initial conditioning (activation cycles). However, in acidic‐media CO_2_R, system‐induced fluctuations in local pH and hydration continuously perturb this stabilization, effectively restarting the dissolution–redeposition loop and leading to a higher net mass loss than that observed under conventional PEMWE conditions [387, 388].

#### 7.3.2 Structural Degradation and Reconstruction

Defects and oxygen vacancies frequently enhance OER activity by facilitating intermediate adsorption; however, they simultaneously weaken lattice stability and accelerate intrinsic structural degradation [95]. In amorphous IrO_x_, high defect densities favor LOM, promoting Ir release and irreversible amorphization [119]. Crystalline IrO_2_ and RuO_2_ catalysts, despite being more robust, often experience local distortion and crack propagation due to repeated redox cycling. Similar structural degradation has been reported for Co–Fe and Mn‐based MMOs, where entropy‐stabilized structures gradually segregate under prolonged polarization [389]. Such processes highlight the delicate trade‐off between catalytic reactivity and structural integrity.

#### 7.3.3 Nanoparticle Agglomeration and Detachment

During long‐term operation, nanoparticles could undergo coarsening through Ostwald ripening–like processes, which are thermodynamically favored but strongly coupled to potential‐driven metal dissolution under acidic‐media OER conditions. In this process, smaller, high‐curvature particles dissolve preferentially and partially redeposit onto larger particles, resulting in coarsened clusters with reduced ECSA [390, 391]. Ex situ SEM analyses of Ir/Ti electrodes after extended OER confirm the enlarged particles and void formation within the CL [392, 393, 394].

While nanoparticle coarsening is intrinsically linked to the thermodynamic and electrochemical instability of the catalyst material, particle migration, sintering, and detachment are predominantly system‐induced processes. Oxygen bubble evolution, local pH gradients, and Joule heating generate mechanical stress within the CL, which accelerates particle displacement and detachment, particularly for unsupported or weakly bound catalysts [389, 395]. Together, these intrinsic and system‐induced processes synergistically diminish the ECSA and accelerate voltage decay during prolonged operation.

#### 7.3.4 Ion Migration, Transport, and Redeposition

While metal dissolution is an intrinsic degradation pathway of acidic‐media OER catalysts, the subsequent migration, transport, and redeposition of dissolved species across the PEM constitute a system‐induced degradation process that is specific to full‐cell operation in acidic‐media CO_2_ electrolyzers. Once released into the electrolyte, dissolved metal cations (e.g., Ir^n^^+^, Ni^2^^+^, and Ti^4^^+^) are driven by the electric field and concentration gradients from the anode toward the cathode [396, 397, 398].

During transport, these cations could accumulate within the PEM, where they disrupt the polymer backbone, reduce proton conductivity, and locally modify the membrane acidity, or reach the cathode, where they are reduced or precipitate as metallic clusters or metal oxides. The latter scenario is particularly detrimental, as redeposited species suppress CO_2_R activity by promoting competing HER [399, 400].

Cross sectional XPS, SEM–EDS, and online ICP‐MS analyses in PEMWEs directly reveal the Ir transport and redeposition within the membrane and cathode layers [396, 397]. Analogous behavior is observed in acidic‐media CO_2_ electrolyzers, where dissolved Ni^2^^+^ species cross over the membrane, forming NiCO_3_/Ni(OH)_2_ deposits that alter product selectivity and raise cell voltage [399]. For Ti‐based PTLs, dissolution of Ti^4^^+^ and formation of TiO^2^^+^/ TiF_a_^(4−a)+^ complexes under high potentials lead to Ti accumulation in the ionomer and cathode, blocking proton‐carrying pathways and accelerating local degradation [401, 402, 403]. Collectively, these processes form a self‐reinforcing degradation loop linking anode dissolution, membrane contamination, and cathode poisoning, ultimately diminishing overall device performance.

#### 7.3.5 System‐Level Implications

The system‐induced transport and redeposition of dissolved species could cause broad consequences for the durability of acidic‐media CO_2_ electrolyzers. First, the loss of active anode material increases the anodic overpotential and decreases efficiency [393]. Second, membrane contamination by cationic species (Ir^n^^+^, Ti^4^^+^, Ni^2^^+^) lowers proton conductivity and initiates chemical attack on PFSA polymers, triggering Fenton‐type reactions [401, 402, 404]. Third, redeposition of metallic clusters on the CO_2_R‐active catalyst suppresses CO_2_R selectivity while triggering competing HER [24, 405]. Together, these effects cause an intertwined deterioration of the electrodes and membrane, undermining device lifetime and energy efficiency.

Anodic degradation remains the principal bottleneck linking catalyst instability to device failure in acidic‐media CO_2_R. The fluctuating acidity at the active sites of the anode accelerates Ir dissolution [406] or Ru volatilization [407]. The dissolved Ir^3^^+^ and volatile RuO_4_ species can migrate across the membrane and poison the CO_2_R sites [408]. These coupled processes connect anode degradation directly to cathode and membrane deterioration. Addressing these challenges requires catalyst designs that not only improve intrinsic corrosion resistance but also mitigate system‐induced degradation, such as corrosion‐resistant coatings (i.e., Ta_2_O_5_ and TiN), robust supports that inhibit metal leaching, and self‐healing or entropy‐stabilized oxides capable of maintaining lattice integrity under dynamic reaction conditions. Such anode innovations are essential to enable system‐level stability and sustainable operation of acidic‐media CO_2_ electrolyzers.

## 8 Design Strategies for Acidic‐Media OER Catalysts

In acidic‐media CO_2_ electrolyzers, the OER plays a key role in overall efficiency and durability. To date, Ir and Ru remain among the few metals capable of retaining structural and chemical integrity under strongly acidic, high‐potential conditions [133, 409, 410]. However, their scarcity and dissolution susceptibility continue to drive efforts to lower noble‐metal loadings while preserving activity. Alloying, support engineering, and design of ultrathin architectures have shown promise in improving utilization and mitigating electrochemical corrosion [408].

In acidic‐media CO_2_ electrolyzers, local proton depletion and hydration or ion‐transport gradients can accelerate anodic degradation, directly linking catalyst stability to overall device performance [366]. Addressing these challenges requires coupling atomic‐scale catalyst design with mesoscale electrode architecture and operational control, including membrane selection and mitigation of potential cycling. Future progress will rely on translating mechanistic understanding into scalable, resource‐efficient systems. This section summarizes recent advances in Ir‐ and Ru‐based OER catalysts and theoretical insights guiding predictive catalyst designs.

Before detailing individual catalyst design strategies, it is important to explicitly connect these approaches to the dominant degradation pathways encountered during acidic‐media OER in CO_2_ electrolyzers. As discussed in Section 7, catalyst degradation primarily originates from noble‐metal dissolution under high anodic potentials, over‐oxidation–induced lattice destabilization, oxygen‐vacancy‐driven structural collapse associated with LOM activity, and interfacial degradation leading to metal‐ion crossover and electrode contamination. Accordingly, effective catalyst design strategies should be evaluated not only in terms of activity enhancement, but also by their ability to suppress specific failure modes. In this context, composition engineering mainly targets electronic and thermodynamic stabilization against dissolution and over‐oxidation; structural engineering governs defect density, lattice strain, and reconstruction pathways that dictate activity–stability trade‐offs; and interface/support engineering mitigates interfacial corrosion, catalyst detachment, and metal‐ion migration. This degradation‐oriented framework provides a mechanistic basis for comparing and integrating different design strategies beyond empirical performance metrics.

### 8.1 Catalyst Design Strategies

#### 8.1.1 Composition Engineering: Maximizing Ir/Ru Utilization

Compositional optimization aims to balance intrinsic OER activity with chemical robustness while minimizing noble‐metal loading [130, 411]. Alloying and doping strategies (i.e., Ir─Ru, Ir─Ni, and Ir─Co) modulate the d‐band center and oxygen‐intermediate binding, improving kinetics and corrosion resistance through electronic and lattice‐strain effects [42, 412, 413]. Ru incorporation enhances activity but accelerates dissolution, whereas Ir stabilizes the Ru lattice, mitigating this trade‐off [42].

HEOs and MMOs distribute oxidative stress across multiple cations, suppressing selective leaching and enhancing acid tolerance [414, 415]. Systems, such as M‐RuIrFeCoNiO_2_ and Ru_0_._6_Cr_0_._2_Ti_0_._2_O_2_, exemplify multi‐metal synergy and configurational‐entropy stabilization during extended electrolysis [251, 384].

Single‐atom and ultra‐low‐Ir architectures further improve atomic utilization efficiency [131]. Isolated Ir atoms anchored on conductive, acid‐stable supports (e.g., nitrides or carbides, such as TiN, TaC, and WC) maintain high activity via optimized Ir–support coupling, where strong Ir─N or Ir─C bonds delocalize charge and suppress Ir^4^^+^ over‐oxidation [128, 129, 416]. Heteroatom doping (i.e., Ti, Sn, and W) enhances Ir─O covalency and limits lattice‐oxygen participation, thereby reducing Ir dissolution [417, 418, 419, 420]. Ti‐doped IrO_2_ thus exhibits markedly slower degradation in acidic‐media OER [123]. Collectively, alloying, entropy‐driven mixing, and atomically dispersed Ir utilization outline a path toward durable acidic‐media OER. From a degradation perspective, these compositional strategies primarily mitigate noble‐metal dissolution and high‐valence over‐oxidation through stabilization of metal–oxygen bonding and suppression of irreversible lattice oxidation pathways under acidic‐media OER conditions.

#### 8.1.2 Structural Engineering: Balancing Activity and Durability

Structural modulation is a central approach to alleviating activity–stability trade‐offs that are intrinsic to Ir‐ and Ru‐based OER catalysts [421]. Introducing controlled defects and lattice strain can expose more active sites and accelerate reaction kinetics, although excessive disorder weakens lattice cohesion [422]. Engineered oxygen vacancies improve conductivity and intermediate adsorption but also promote Ir or Ru dissolution under high anodic potentials. Accordingly, strain engineering seeks to balance intermediate binding with structural robustness [423]. Self‐healing and dynamic reconstruction have emerged as key pathways for prolonged durability [424]; in situ spectroscopy reveals that hydrous IrO_x_ can reversibly interconvert between Ir^3^^+^ and Ir^4^^+^, reorganizing into corrosion‐resistant crystalline domains during operation [119]. Mechanistic analyses demonstrate that the PDS shifts with surface reconstruction: on pristine IrO_2_, OOH formation controls kinetics, whereas vacancy‐rich lattices activate LOM. While this phenomenon accelerates OER, it could also amplify dissolution [425]. Strengthening Ir─O bonds through Ti or W doping effectively suppresses LOM and improves stability [123, 426, 427]. In this regard, structural engineering directly regulates whether OER proceeds via benign surface reconstruction or detrimental LOM‐dominated pathways that accelerate oxygen‐vacancy formation, lattice collapse, and metal dissolution. Entropy‐stabilized multi‐cation lattices further combine defect control and self‐healing behavior, dissipating electrochemical stress and delaying segregation [130, 226]. Together, these mechanisms establish a robust structural framework for sustaining high‐current acidic‐media CO_2_R [11].

#### 8.1.3 Interface and Support Engineering: Protecting Active Sites

The catalyst–support interface is a primary determinant of electrochemical durability in acidic‐media OER and CO_2_ electrolyzers because it governs charge transfer, local redox equilibria, and structural evolution [428]. SMSI enhances adhesion, conductivity, and corrosion resistance; operando spectroscopy has revealed that site‐specific MSI can even toggle the activity of isolated Ir atoms [416]. For Ir‐ and Ru‐based catalysts, conductive, acid‐tolerant supports, such as TiN and oxygen‐deficient niobium or tantalum oxides (i.e., Nb_2_O_5__−__x_ and Ta_2_O_5_), create interfacial charge redistribution and mechanical confinement, thereby suppressing nanoparticle detachment, Ostwald ripening, and ECSA loss [391, 429, 430].

Protective overlayers and diffusion barriers—ultrathin Ta_2_O_5_ or TiO_2__−__x_ shells and conductive carbon coatings—further isolate active metals from aggressive anions (i.e., F^−^ and CO_3_^2^^−^/HCO_3_^−^) while maintaining electronic connectivity. Operando ICP‐MS and XAS confirm that such layers markedly retard Ir dissolution and stabilize oxidation states under high current densities [431, 432, 433]. By stabilizing the catalyst–support interface and blocking direct exposure to aggressive ionic species, these strategies specifically address interfacial degradation pathways, including nanoparticle detachment, support corrosion, and metal‐ion crossover that compromise full‐cell durability in acidic‐media CO_2_ electrolyzers. Beyond classical MSI, dual‐site coupling between single atoms and adjacent clusters provides additional stabilization: isolated Ru atoms adjacent to RuO_x_ or IrO_x_ nanoclusters share charge density, resist over‐oxidation, and promote redox reversibility [434, 435].

In acidic‐media CO_2_ electrolyzers, engineered interfaces and barriers also hinder metal‐ion crossover, mitigating Ir contamination of the cathode and sustaining CO_2_R selectivity [436]. These insights point toward minimal‐Ir/Ru architectures embedded in corrosion‐resistant, high‐entropy, or hybrid frameworks that preserve performance while advancing resource sustainability [62, 370]. Taken together, composition, structure, and interface engineering forms a complementary degradation‐mitigation toolbox in lieu of functioning as isolated optimization routes. Their combined implementation enables simultaneous suppression of metal dissolution, control of lattice‐oxygen activity, and stabilization of catalyst–electrode interfaces, which are all essential for sustaining high‐current operation in acidic‐media CO_2_ electrolyzers.

### 8.2 Theoretical Insights into Catalyst Designs

Understanding the atomic and electronic origins of catalyst activity and stability in acidic media is crucial for rational catalyst design. Recent progress in first‐principles simulation, data‐driven exploration, and multi‐scale modeling has provided fundamental guidance for optimizing Ir‐ and Ru‐based OER catalysts under realistic electrolysis conditions [139, 437, 438].

#### 8.2.1 DFT‐Based Mechanistic Understanding

DFT studies provide atomistic insights into acidic‐media OER pathways and performance degradation. On well‐ordered rutile IrO_2_ surfaces, AEM is often observed, whereas hydrous/defective IrO_x_ and some Ru‐rich oxides can activate LOM that boosts rates but triggers dissolution [119, 439]. Defect‐rich sites lower O─O coupling barriers, accelerating kinetics while weakening metal–oxygen bonding, thus rationalizing the activity–stability trade‐offs [427]. Operando‐anchored models link the build‐up of high Ir valence (Ir^>4+^) and dynamic disorder to elevated dissolution probability, rather than a single deterministic step [440]. Strain calculations further show that lattice strain tunes Ir─O covalency and adsorbate binding; tensile strain enhances activity but compromises cohesion if excessive [441]. Beyond idealized slabs, explicit electrolyte/anion effects modulate interfacial fields and intermediate binding; at the device level, potential cross‐over of CO_2_‐derived (bi)carbonate crossover (even at minimum levels) could alter the anode environment and aggravate Ir loss, consistent with modeling trends [436, 438]. These results highlight the need for coupling DFT studies with realistic potentials/solvation and operando validation when interpreting durability trends in acidic‐media OER.

Despite major progress, standard DFT workflows still struggle to capture the full electrochemical complexity of acidic‐media OER interfaces. Particularly, the calculations often rely on vacuum, fixed‐charge, and 0 K approximations, thereby underrepresenting solvent dynamics, potential control, and double‐layer effects that govern stability and kinetics [166, 177]. In practice, GGAs can misrepresent redox energetics of high‐valent Ir/Ru species, motivating hybrid functionals (HSE06) or DFT+U to improve Ir─O/Ru─O electronic structure and cohesive‐energy predictions [169]. To overcome these limitations, grand‐canonical/constant‐potential DFT and AIMD with explicit water/ions have been adopted to include electrode potential, solvation, and dynamic reconstruction [168, 169, 173, 177]. At the reactor‐scale, microkinetic (MKM) and kinetic Monte Carlo (kMC) models translate DFT energetics into rate constants and coverages, enabling mechanism‐level interpretation under operating conditions [442]. Complementarily, machine‐learning‐accelerated screening and interface‐aware modeling broaden accessible timescales and composition space, while operando spectroscopies provide the validation loop that constrains models to reality [438]. Together, these multi‐scale strategies extend DFT calculations beyond static approximations and deliver quantitatively testable guidance for optimizing Ir‐ and Ru‐based OER catalysts under realistic electrolysis conditions.

#### 8.2.2 Machine Learning and High‐Throughput Screening

Machine‐learning (ML) and high‐throughput computational strategies are transforming the exploration of complex Ir‐ and Ru‐based oxides for acidic‐media OER. ML models trained on DFT‐derived descriptors—including *OH, *O, *OOH adsorption energies, metal–oxygen covalency, and calculated dissolution potentials—can rapidly estimate catalytic activity and stability across large compositional spaces [384, 437, 438].

Recent ML‐guided searches have identified multi‐metallic systems, such as Ir–Ru–Ti–W and Ir–Ru–Mo, as promising combinations for acid‐tolerant OER [109, 384]. These models suggest that minor Ir incorporation (a few atomic percent) into multi‐metal frameworks can preserve high activity and lattice robustness, whereas excessive Ir yields could diminish stability benefits. High‐throughput DFT and Bayesian optimization workflows further construct activity–stability volcano relations, quantifying how Ir content, lattice distortion, and dissolution potential co‐evolve [438, 443]. Such analyses provide quantitative guidance for compositional tuning in high‐entropy and doped IrO_2_ systems.

The convergence of ML prediction and operando validation establishes a closed feedback loop for catalyst discovery. When coupled with automated synthesis and accelerated electrochemical screening, these approaches enable adaptive composition optimization in response to measured durability trends [444, 445, 446, 447]. Emerging reinforcement‐learning and generative‐design frameworks continue to demonstrate proof‐of‐concept autonomous searches over vast composition spaces, dynamically steering synthesis toward regions of predicted acid stability [448, 449, 450, 451].

#### 8.2.3 Pourbaix Diagrams and Thermodynamic Predictions

**FIGURE 9:**
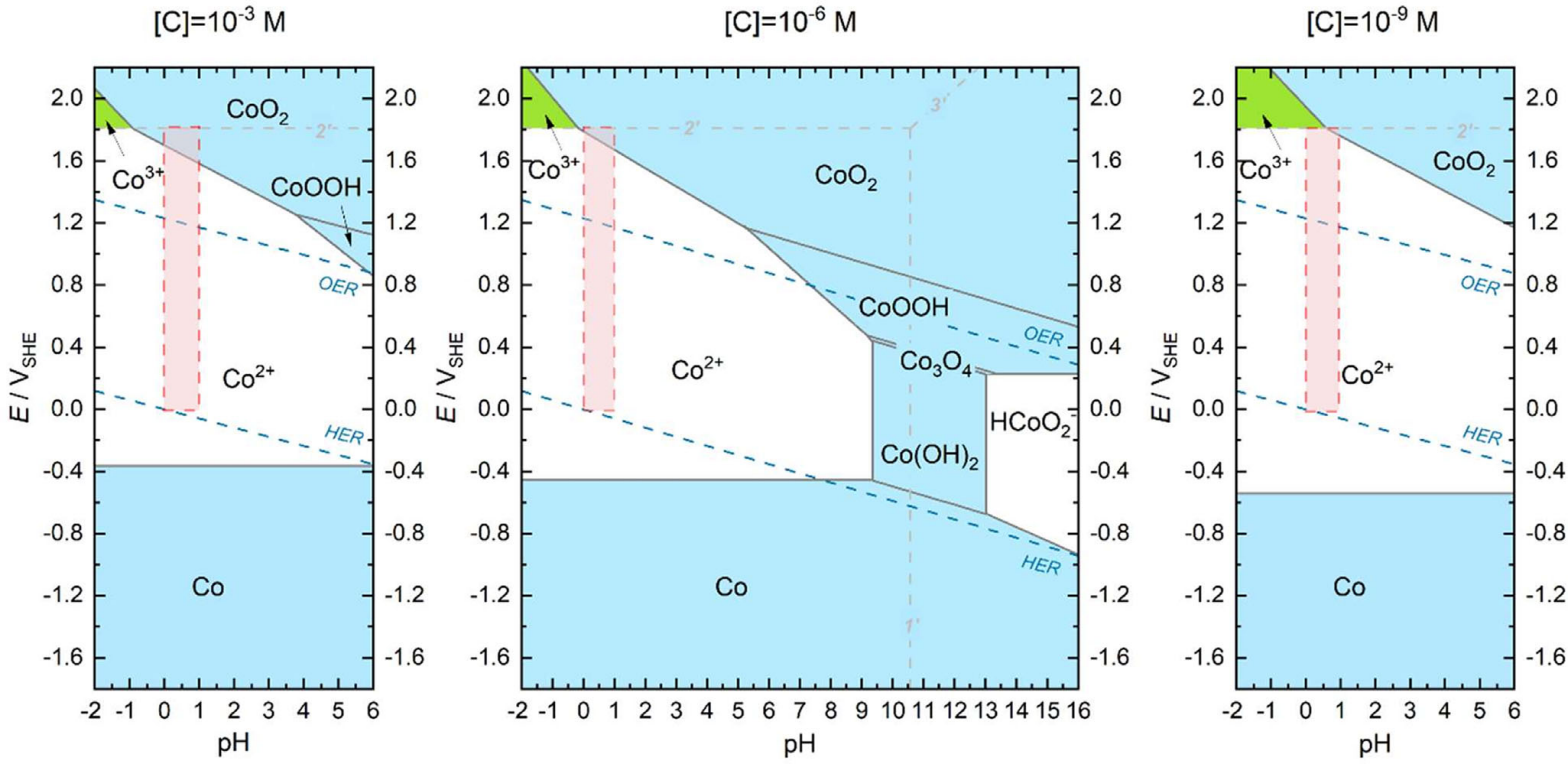
Pourbaix diagrams of cobalt (Co) calculated using experimental thermodynamic tables with aqueous ion concentrations of 10^−3^ m (left), 10^−6^ m (center), and 10^−9^ m (right) at 25°C. Reproduced with permission [458]. Copyright 2025, American Chemical Society.

Pourbaix (E–pH) diagrams are indispensable tools for assessing aqueous and electrochemical thermodynamic stability, as they map transitions among metallic, oxide, and dissolved species and delineate redox boundaries relevant to corrosion and dissolution processes (Figure 9) [452, 453, 454]. Unless otherwise specified, the Pourbaix diagrams discussed in this section primarily refer to conventional (bulk) thermodynamic Pourbaix diagrams, which evaluate the relative stability of bulk solid phases against dissolution into aqueous species as a function of pH and electrode potential [455, 456]. Such bulk Pourbaix maps have been widely adopted in acidic‐media OER studies as first‐order thermodynamic descriptors, despite their inherent limitations in capturing surface‐specific terminations and near‐surface reconstructions [457].

It should be emphasized that Pourbaix diagrams are not unique; they depend on the assumed activity (concentration) of dissolved metal ions in the electrolyte. As illustrated in Figure 9, decreasing the aqueous Co ion concentration from 10^−^^3^ to 10^−^^9^ M systematically shifts the phase boundaries and expands the thermodynamic corrosion regions [458]. This concentration dependence reflects the Nernstian contribution of dissolved species to solid–aqueous equilibria and is particularly relevant for acidic‐media OER conditions, where the initial concentration of dissolved metal ions is typically very low. Consequently, bulk Pourbaix diagrams should be interpreted as concentration‐dependent thermodynamic maps rather than absolute stability limits, especially when correlating them with operando dissolution measurements.

For Ir‐ and Ru‐based catalysts, bulk Pourbaix analyses—often combined with experimental, time‐ and potential‐resolved scanning flow cell inductively coupled plasma mass spectrometry (SFC‐ICP‐MS) measurements—indicate intrinsically restricted stability windows under strong acidic conditions and high anodic bias [133, 459, 460]. These results rationalize the long‐standing challenge of sustaining noble‐metal oxides during acidic‐media OER. In recent works, incorporating improved energetics and solvation treatments (e.g., SCAN‐based thermodynamic frameworks) has further refined bulk stability fields and enabled more consistent estimates of dissolution onset compared with experimental observations [166, 453].

It should be noted that catalyst degradation during acidic‐media OER frequently originates from surface terminations and near‐surface reconstructions that are not explicitly resolved in bulk Pourbaix diagrams. To address this limitation, surface Pourbaix diagrams, which explicitly account for surface‐specific structures and adsorbate‐covered states, have been developed for selected model systems and provided deeper insights into surface oxidation and reconstruction pathways. Nevertheless, due to their substantially increased computational complexity and strong dependence on specific surface models, surface Pourbaix analyses are currently applied to case studies rather than large‐scale catalyst screening. As a result, bulk Pourbaix diagrams remain the dominant thermodynamic framework in the broader acidic‐media OER literature.

Linking bulk Pourbaix analysis with operando dissolution measurements provides a kinetic–thermodynamic perspective that helps explain why experimentally measured Ir loss may deviate from equilibrium boundaries [22, 460]. In acidic‐media CO_2_ electrolyzers, spontaneous variations in local pH, ionic strength, and interfacial electric fields at the anode can further accelerate Ir dissolution, even when specific Ir–carbonate complexes are not dominant under acidic conditions [457].

Building on this thermodynamic foundation, the Pourbaix‐enabled guest synthesis (PEGS) strategy selects deposition conditions within electrochemical windows where both host and overlayer phases are thermodynamically stable [431, 461]. This approach enables the formation of self‐passivating overlayers (e.g., Ta_2_O_5_ or Nb_2_O_5_) that resist corrosion while preserving catalytic performance. Combining Pourbaix‐guided selection with electronic‐structure modeling thus provides predictive guidance for designing corrosion‐resistant supports and coatings for dynamic acidic environments.

#### 8.2.4 Integrated Theory–Experiment Feedback and Cross‐Scale Design

A comprehensive understanding of acidic‐media OER requires feedback across computation, operando characterization, and device‐level diagnostics. First‐principles predictions of Ir─O bonding, dissolution thermodynamics, and defect/strain effects are increasingly tested by operando XAS/XPS, ^1^ ^8^O‐DEMS, and online ICP‐MS, which together correlate atomic‐scale transformations with Ir loss [106, 436, 462]. Strain‐dependent modeling shows that lattice strain tunes Ir─O covalency and adsorbate binding, and operando X‐ray studies capture concomitant structural responses under OER bias, connecting activity gains to stability penalties when strain is excessive [391, 463]. Beyond idealized models, constant‐potential DFT/AIMD with explicit solvation improves the realism of interfacial energetics and reconstruction pathways [133].

At the device level, ICP‐MS mass balances and fluoride‐emission‐rate (FER) diagnostics identify membranes and cathodes as major sinks for dissolved Ir and link membrane degradation products (F^−^/HF) to anode durability, providing constraints back to materials design [436]. Closing the loop, integrating DFT/AIMD/ML with operando spectroscopy and ASTs could establish predictive, testable targets for Ir utilization, stability, and recyclability in acidic‐media CO_2_ electrolyzers [106, 462].

## 9 Characterization Techniques

A comprehensive understanding of acidic‐media OER in CO_2_ electrolyzers requires integrated theory–experiment feedback. The proton concentration fluctuations and pH gradients create dynamic interfaces distinct from PEMWEs [366, 464]. Characterizing these environments under realistic conditions is essential to validate theoretical predictions of metal dissolution, defect evolution, and lattice‐oxygen participation. More specifically, operando XAS/XPS can track oxidation and coordination changes, ^1^ ^8^O‐DEMS can identify lattice‐oxygen involvement, and online ICP‐MS can quantify Ir dissolution and redeposition [22, 106, 426, 436]. Such complementary techniques can link atomic‐scale reconstruction and ion migration to macroscopic degradation, bridging computational modeling with experimental observation and informing rational catalyst design for durable acidic‐media CO_2_R.

### 9.1 In Situ Characterization

In situ characterization serves as a powerful tool to identify degradation mechanisms and develop mitigation strategies. These methods enable real‐time monitoring of catalyst surface and micro‐reaction environment. Such insights can widen our understanding of micro‐reaction environments, bridging the gap between theoretical understanding and practical applications. In situ spectroscopies could also provide insights into the dynamic structural evolution of catalysts [465]. During OER, the highly oxidative micro‐reaction environment causes catalysts to undergo transformations, such as phase changes, surface restructuring, and oxidation‐state variations [319, 432, 466]. Mechanistic understanding of degradation mechanisms and degradation profiles, i.e., catalyst dissolution, over‐oxidation, and loss of structural integrity, could be instrumental in developing mitigation strategies. The most common in situ characterization techniques, along with the measurement principle and obtained information, are summarized in Table 3.

**TABLE 3:** A summary of in situ characterization, source of signal, and insights. Adapted from ref. [465]. Copyright 2024, Springer Nature.

Method	Source of signal	Available information
XAS	Synchrotron X‐ray	XANES probes the oxidation state of the catalysts EXAFS probes the bond geometry and coordination of the catalysts
XPS	X‐ray	Chemical environment of element in catalyst
XRD	X‐ray	Structural order and phase identification
IR	Laser	Spectra information of adsorbed species
Raman	Laser	Molecular structures in the low‐frequency region and reaction intermediates
ICP‐MS	Ion source	On‐line dissolution of catalysts
DEMS	Ion source	On‐line signals of volatile reaction intermediates/products
HRTEM	Electron beam	Morphology and structure parameters of crystal
EELS	Electron energy	Element identification and distribution
EQCM	Resonance frequency	Mass change of catalysts during electrocatalysis
UV‐Vis	Optical radiation	Electron and charge transfer transitions of transition metal ions

EIS evaluates resistive and capacitive behaviors [467], elucidating charge transfer resistance, ionic conductivity, and mass transport limitations (Table 3). EIS involves measuring the impedance, which is represented by Nyquist or Bode plots [468]. Obtaining these plots is critical for frequency‐dependent understanding of the system's resistive, capacitive, and inductive attribute [469]. These attributes are influenced by charge transfer, ion transport, and mass diffusion. Monitoring voltage/current changes could enable identification of performance inconsistencies induced by non‐uniform reactant distribution or component degradation. Identifying the voltage/current variations in three‐electrode electrochemical cells – when benefitted from control experiments – offers detailed analysis of individual constituents, including OER‐active anode and PTLs [470].

Raman spectroscopy and in situ infrared (IR) spectroscopy could track the evolution of active configurations and pinpoint the key catalytic species (Table 3). Such insights are critical to optimizing catalyst design and enhancing OER performance. Rationalizing the mechanistic pathways of OER is another avenue. The OER involves multiple intermediates, including *OH, *O, and *OOH. The formation and transformation of these intermediates dictate the overall reaction efficiency [73, 471]. Detecting the OER intermediates in real time could be effective in elucidating the major OER mechanisms, i.e., AEM and LOM. Additionally, surface‐enhanced Raman spectroscopy (SERS) enhances signal detection, enabling precise identification of reactive species and catalytic pathways. In situ IR spectroscopy could provide molecular insights, which can be instrumental in mapping OER pathways and identifying RDSs. Additionally, attenuated total reflection infrared (ATR‐IR) and surface‐enhanced infrared absorption (SEIRA) could identify the adsorbed reaction intermediates on the catalyst surface [472]. These methods detect key species, i.e., *OOH and *O, elucidating the RDSs and catalytic mechanisms [88, 252, 473]. ATR‐IR and SEIRA, by monitoring changes in vibrational spectra, offer direct evidence of intermediate formation, clarifying the roles of specific active sites in the OER process [474].

XAS based on X‐ray near‐edge structure (XANES) and extended X‐ray absorption fine structure (EXAFS) could elucidate the active species’ oxidation state and coordination environment (Table 3). XANES allows monitoring the changes in valance state, elucidating the oxidation and reduction processes that influence catalytic activity [475]. EXAFS could provide information on the local atomic structure, offering insights into bond lengths, coordination numbers, and dynamic rearrangements of metal‐oxygen bonds during OER. These features render XAS instrumental in correlating catalyst structure with OER performance [476, 477].

In situ XPS enables real‐time investigation of surface chemical states, providing information about oxidation states, hydroxylation, and compositional changes (Table 3). Unlike ex situ XPS, which can be affected by post‐reaction transformations, in situ XPS captures the surface modifications in real time [478]. This technique is useful in understanding how the catalyst surface evolves during OER, offering insights into stability and degradation mechanisms.

DEMS enables real‐time analysis of gaseous reaction products (Table 3). By detecting oxygen isotopes and tracking their evolution, DEMS can differentiate the source of oxygen, i.e., water molecules and lattice oxygen [99, 479, 480]. This distinction is crucial for revealing the dominant reaction mechanism and assessing catalyst stability [217].

In situ XRD enables monitoring the phase changes in real time [481]. Metal oxides and perovskite‐based materials typically undergo structural transformations. Such transformations can enhance or degrade performance (Table 3). By tracking the crystalline phases, in situ XRD can elucidate the interplay between electrochemical activity and material stability, guiding the design of robust catalysts.

High‐resolution transmission electron microscopy (HRTEM) and electron energy loss spectroscopy (EELS) provide nanoscale insights into catalyst morphology and elemental distribution (Table 3). They allow for the direct visualization of structural evolution, including particle dissolution, agglomeration, and active site formation [71]. EELS further assists in identifying the atomic‐level oxidation state changes, elucidating how catalysts evolve during OER.

Ultraviolet‐visible (UV–vis) spectroscopy identifies the electronic transitions and oxidation state changes (Table 3). UV–vis spectroscopy — by measuring the absorbance variations — determines the effect of electronic structure on catalytic activity. UV–vis spectroscopy could also probe the ligand‐field interactions and d‐band modifications – both are the key parameters impacting the efficiency of OER. In situ ICP‐MS enables online, ultra‐sensitive monitoring of precious metal dissolution during OER. Additionally, ICP‐MS quantifies S‐number and reveals transient dissolution mechanisms. When combined with other in situ techniques, it provides insights into stability correlations and structural evolution [471].

Electrochemical quartz crystal microbalance (EQCM) employs a catalyst‐coated AT‐cut quartz crystal to monitor real‐time resonant frequency shifts. These shifts are converted into mass changes, enabling synchronized mass‐charge coupling measurements [394]. This technique precisely quantifies minute metal dissolution rates and assesses short‐term stability via S‐number. Furthermore, analyzing the mass fluctuations at different potentials distinguishes oxide dissolution, bubble evolution, and adsorption/desorption processes [317]. Such insights provide critical evidence for surface restructuring and intermediate accumulation in the CL.

Together, these diagnostic tools could provide mechanistic insights into the dissolution–migration–redeposition cycle that governs the lifetime of acidic‐media CO_2_ electrolyzers. When combined with in situ monitoring of humidity and temperature across the electrode–membrane interface, these techniques could yield a comprehensive picture of how ionic transport and mass exchange dictate both catalyst durability and device efficiency.

### 9.2 Ex Situ Characterization

Ex situ characterization complements in situ/operando analyses by documenting the permanent structural, chemical, and morphological changes after extended acidic‐media CO_2_R. After disassembly, the CLs and PTLs performing the OER are examined to correlate long‐term structural evolution with stability mechanisms inferred from operando results. Microscopy techniques—SEM, AFM, and TEM—diagnose particle agglomeration, support corrosion, crack formation, and interface degradation. Upon completion of acidic‐media CO_2_R, these analyses often reveal Ir or Ti redeposition/amorphous surface films, consistent with a dissolution–migration–redeposition cycle [22, 403, 436]. HRTEM resolves lattice distortions and nanoscale amorphization aligned with reconstructions suggested by theory/operando spectroscopy [394]. Spectroscopic techniques—XPS and FTIR—identify oxidation‐state shifts and oxide/fluoride deposits at the anode, linking interfacial chemistry to durability [366, 482]. Complementary surface‐area/porosity probes (BET, mercury intrusion, and small‐angle scattering) quantify texture evolution that governs gas–liquid transport and electrolyte accessibility, holding a potential to connect microstructure to macroscopic performance degradation during acidic‐media CO_2_R [62].

Collectively, ex situ analyses provide post‐mortem validation of mechanisms revealed by operando studies. By linking microstructural and chemical degradation to measurable performance losses, they close the feedback loop between observation and design, offering a foundation for optimizing the architecture of CLs and PTLs in acidic‐media CO_2_ electrolyzers.

## 10 Electrode Architecture for Acidic‐Media OER

Electrode architecture plays a decisive role in governing the activity, stability, and scalability of acidic‐media OER systems. While catalyst composition determines intrinsic activity, the electrode structure dictates mass transport, interfacial contact, and long‐term durability at high current densities. In acidic‐media CO_2_ electrolyzers, the anodic environment is further complicated by local pH gradients/fluctuations, demanding advanced control of transport, wetting, and corrosion. Operando diagnostics have revealed that degradation often originates from structural and interfacial limitations within the PTL, CL, and their junction. Building on these insights, the following section discusses architecture‐driven strategies—including PTL engineering, PTL material substitution or surface modification, and CL design—to achieve durable, low‐resistance, and scalable electrode assemblies for sustained acidic‐media CO_2_R.

### 10.1 PTLs for Acidic‐Media OER

**FIGURE 10:**
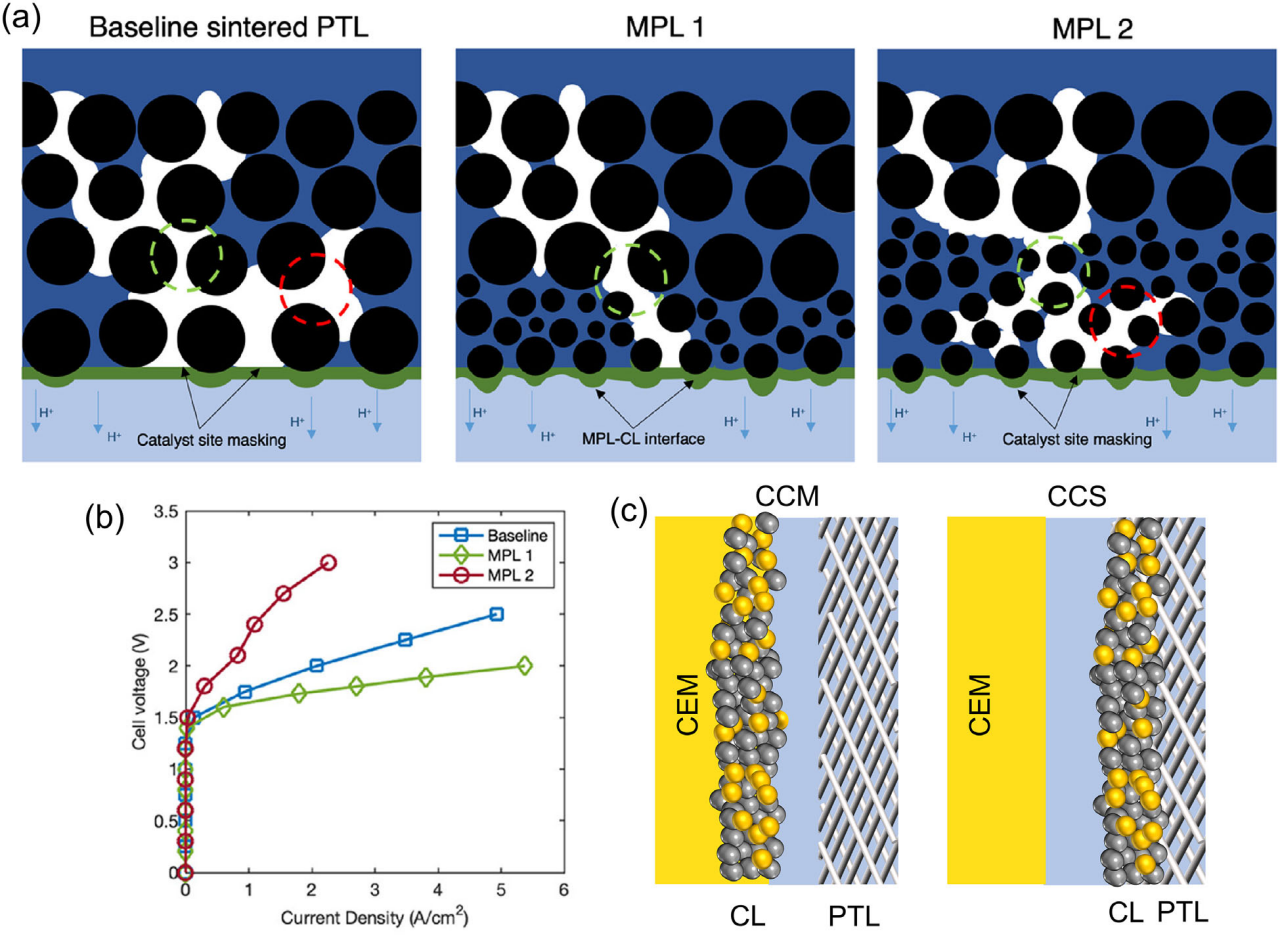
Time‐averaged oxygen saturation in a single layer baseline sintered PTL. (a) Oxygen gas invasion patterns through traditional PTL, PTL with thin MPL, and PTL with thick MPL. Green dotted circles represent the primary advancing oxygen finger, and red dotted circles represent the lateral displacement of oxygen fingers. (b) Polarization curves obtained from the PEMWEs with three different PTLs. (a,b) Reproduced with permission [485]. Copyright 2023, American Chemical Society. (c) Diagrams showing the configuration of CCM and CCS. Reproduced with permission [489]. Copyright 2023, American Chemical Society.

The PTL—a porous, hydrophilic, and oxidation‐resistant component positioned between the CL and bipolar plate (BP)—plays a decisive role in acidic‐media OER by facilitating mass, electron, and heat transport while providing mechanical support under oxidative conditions [483, 484, 485]. The PTL regulates electrolyte distribution and oxygen removal, with porosity, pore size, and thickness determining transport efficiency [486, 487]. As illustrated in Figure 10a,b, optimized pore‐size gradients enhance mass transfer within the PTL, minimizing diffusion resistance and improving overall performance [485]. Particularly, hydrophilic materials promote uniform electrolyte wetting and suppress bubble accumulation. Additionally, graded‐pore architectures further improve oxygen evacuation and reduce transport losses [488].

Besides mass transport, the PTL serves as a conductive pathway for electrons [490]. Ti‐based PTLs are widely adopted owing to their high conductivity and corrosion resistance in acidic media. However, uncoated Ti is prone to passivation through TiO_x_ formation, which increases interfacial resistance and necessitates protective coatings or alloying strategies. Although Pt‐ and Ir‐based coatings enhance conductivity and corrosion resistance, their high cost drives research toward alternative materials and coating methods that balance durability and affordability.

PTLs also contribute to thermal management, particularly in large‐area stacks (>100 cells, >1000 cm^2^). The OER generates substantial heat; thus, PTLs with high thermal conductivity dissipate heat effectively. Herein, optimization of PTL structure will be critical to maintaining the optimal temperature range of 40–45°C and preventing membrane or catalyst degradation in acidic‐media CO_2_R systems. Simultaneously, the PTL provides mechanical stability, maintaining intimate interfacial contact and mitigating stress from differential swelling or compression [484]. Advanced surface designs—such as microporous layers or smooth‐graded interfaces—enhance interfacial conformity and maximize catalyst utilization.

Recent designs exploit graded porosity, with finer pores adjacent to the CL and coarser pores near the flow channels [491]. This configuration improves interfacial contact, catalyst utilization, electrolyte delivery, and oxygen removal, reducing mass‐transport overpotentials and supporting operation at high current densities [492, 493]. Efforts to enhance the acid resistance of such graded PTLs include applying Ir or TiN coatings, which preserve structural integrity during long‐term electrolysis [494, 495].

Microstructural and interfacial engineering efforts have therefore become central to PTL optimization. Techniques, such as tape‐casting of Ti powders with polymeric pore formers, allow precise and scalable control of pore size and porosity [496]. Balancing mechanical integrity and permeability is crucial, since excessive densification hinders bubble release while larger pores facilitate gas evacuation. Diffusion‐bonded hybrid PTLs, combining sintered Ti layers with Ti meshes, deliver low contact resistance and efficient two‐phase flow, supporting current densities of 6 A cm^−^^2^ at 90°C and 90 bar with performance comparable to that of commercial devices [497]. X‐ray computed tomography (XCT) and modeling confirm that graded‐porosity architectures suppress oxygen buildup and enhance reactant accessibility, reducing polarization losses at high current densities [498]. Moreover, in situ XCT studies of catalyst‐coated membranes (CCMs) and PTLs reveal that surface flatness and contact uniformity critically influence proton transport and water accessibility; smoother PTLs consistently yield lower overpotentials and improved durability [498, 499]. Collectively, these results emphasize that achieving optimal PTL performance requires coordinated control of microstructure and interface, ensuring uniform reactant distribution, minimum resistance, and sustained stability.

### 10.2 Catalyst Layers (CLs) for Acidic‐Media OER

The CL serves as the electrochemically active interface where OER occurs, and its architecture dictates not only the ECSA and gas‐release behavior but also the mechanical adhesion to the PTL or PEM [483]. Two main configurations dominate acidic‐media OER systems: the CCM and the catalyst‐coated substrate (CCS) approaches [212, 500]. Both aim to optimize catalyst–membrane interfaces to maximize efficiency and durability under harsh acidic operation.

In the CCM configuration, the catalyst is deposited directly onto the PEM, ensuring intimate contact and minimizing ionic resistance (Figure 10c). This thin, uniform layer improves proton transport and noble‐metal utilization [501, 502]. However, mismatched thermal expansion and hydration between the CL and PEM can cause delamination or structural degradation. Thus, fabrication requires precise control to prevent defects, such as pinholes that could compromise electrolyzer performance [372, 503].

By contrast, the CCS configuration deposits catalysts onto a porous substrate—typically a Ti‐based PTL—which is later assembled with the membrane (Figure 10c). This design offers greater mechanical stability and tolerance to operational stress [504], while allowing diverse deposition techniques (i.e., spraying, sputtering, and electrodeposition) that tailor porosity and reactant transport [505]. The additional interface, however, introduces extra ionic resistance, reducing efficiency compared with CCM [506]. Despite this limitation, CCS structures are easier to fabricate, displaying longer operational lifetimes and remaining robust under high currents and pressures [507].

Optimization of CL composition and structure is therefore essential. Studies on IrO_2_–ionomer composites show that the ionomer content must balance proton conduction and gas transport: insufficient ionomer content increases charge‐transfer resistance, whereas excess ionomer blocks pores and impedes oxygen evacuation [508]. Additionally, matching catalyst morphology to PTL texture ensures uniform adhesion and minimizes interfacial voids. Electrochemical–microscopic analyses under ASTs confirm that noble‐metal coatings, such as Pt or Ir, gradually dissolve during potential cycling, releasing metal ions that migrate into the membrane and initiate radical formation [320]. The ions could also reach to negatively charged cathode electrode, blocking the active sites of the CO_2_R‐active catalyst, suppressing selectivity and triggering competing HER.

Emerging hybrid CCM/CCS designs, which integrate a dense ionomer‐rich sublayer with a porous catalytic overlayer, combine mechanical robustness with efficient proton access [18]. Moreover, using conductive oxides (i.e., Sb‐doped SnO_2_, Ta_2_O_5_, and Nb_2_O_5_), carbides (i.e., TiC and WC), or nitrides (i.e., TiN and TaN) as catalyst supports enhances conductivity and corrosion resistance [409]. Collectively, these developments highlight that coordinated control of composition, microstructure, and interfacial contact is essential to achieving durable, low‐resistance, and scalable electrode architectures for acidic‐media CO_2_R.

### 10.3 Substitution and Modification Strategies for PTL Materials

Ti foams and felts remain the dominant PTL choice for acidic‐media OER due to their mechanical robustness. However, under highly oxidative potentials, Ti surfaces progressively oxidize to form an insulating TiO_2_ layer, increasing interfacial contact resistance (ICR) and releasing Ti^4^^+^ ions into the electrolyte. These degradation phenomena, observed through operando ICP‐MS and cross sectional SIMS analyses, lead to voltage decay, reduced catalyst adhesion, and metal‐ion crossover, contaminating the membrane and cathode [320, 509]. Addressing these issues requires either replacing Ti as the bulk scaffold or modifying its surface to suppress passivation and dissolution while preserving mechanical and electrical properties.

Recent studies have explored several approaches to enhance PTL stability. One practical route employs hybrid metal architectures, where stainless steel substrates are coated with dense Ti layers using cold‐gas spraying or plasma deposition [510]. When the Ti layer exceeds ∼50 µm, corrosion resistance and conductivity match those of pure Ti, enabling cost reduction and scalability. Another effective solution is introducing refractory interlayers of Nb or Ta between Ti and the electrolyte; these elements form conductive and stable oxides that mitigate dissolution and lower ICR under prolonged electrolysis [509]. In parallel, conductive nitride or carbide barriers (i.e., TiN, TaN, and NbC) deposited by sputtering or atomic‐layer deposition offer high chemical inertness and electronic conductivity, acting as durable interfacial shields against acid‐induced oxidation. Additionally, electrodeposited noble‐metal (i.e., Pt, Ir, and Au) coatings create corrosion‐resistant skins; notably, uniform Au electroplating throughout Ti PTL pores enhances conductivity and extends lifetime, enabling a voltage improvement of 120 mV at a current density of 2 A cm^−^^2^ [510].

Surface structuring complements these chemical modifications. Laser texturing and MPLs help manage OER and water distribution, mitigating bubble accumulation [496, 498]. Additive manufacturing (AM) methods, such as laser powder‐bed fusion, enable precise control of pore morphology and channel orientation, allowing in situ integration of protective nitride or carbide coatings during printing [497]. Collectively, these advancements support the development of PTLs with tailored porosity, high corrosion resistance, and low resistance, bridging material innovation and system durability for next‐generation acidic‐media CO_2_ electrolyzers.

## 11 Effect of Operating Parameters on OER

This section examines how external operating parameters interact with electrode structure and material properties to dictate the performance, efficiency, and stability of acidic‐media OER. Key parameters—such as current density, voltage, electrolyte composition, temperature, and compression—exert complex, often coupled, influences on charge transfer, mass transport, and degradation kinetics. Understanding and managing these interactions are essential not only for achieving high energy efficiency but also for ensuring long‐term mechanical and electrochemical integrity under industrially relevant acidic‐media CO_2_R. Recent reports on acidic‐media CO_2_R further reveal that these operating parameters simultaneously influence OER and CO_2_R [511]. In these systems, parameters, such as temperature, current density, and electrolyte composition, jointly determine the local pH balance, ion migration across the membrane, flooding phenomena, and system sustainability [511].

### 11.1 Current Density and Potential

Current density and applied potential directly control the reaction rate, energy efficiency, and durability of acidic‐media OER. At low overpotentials, the process is limited by intrinsic kinetics; as current density increases, mass‐transport constraints and thermal effects dominate. High current densities enhance HER or CO_2_R productivity but simultaneously elevate overpotential, reducing overall energy efficiency. Moreover, excessive current density accelerates the oxidation of active sites and the formation of volatile species, such as IrO_4_ and RuO_4_, which dissolve into the electrolyte and cause catalyst loss. Operando studies show that these dissolution and redeposition processes are often accompanied by mechanical stress and particle detachment from the substrate, causing cumulative degradation [512].

In practical large‐area electrodes, current distribution is rarely uniform. Variations in PTL porosity, surface flatness, or interfacial voids can cause localized current “hot spots”, intensifying electrochemical stress and accelerating failure. Advanced current mapping and numerical simulations reveal that tailoring PTL porosity gradients and surface roughness can homogenize current flow, minimize overpotential, and delay degradation.

Recent investigations into acidic‐media CO_2_R/OER systems confirm that operating current density also dictates the ionic flux and pH gradients across the membrane, directly influencing OER kinetics and catalyst stability [511]. At high current densities, the cathodic environment becomes locally neutral due to alkali cation migration and (bi)carbonate formation, while the anodic side experiences increased acidity, resulting in uneven potential distribution and enhanced dissolution [511]. Therefore, determining an optimal current density requires balancing productivity with longevity. Adaptive voltage‐control strategies that limit transient over‐oxidation and local heating can sustain operation at high current densities while preventing irreversible structural damage, highlighting the need for operation–design co‐optimization.

### 11.2 Electrolyte and Chemical Environment

The chemical nature of the electrolyte profoundly affects reaction kinetics and catalyst durability. While strongly acidic environments (pH <1) ensure high proton conductivity, such conditions also accelerate the corrosion of even noble‐metal‐based catalysts. Buffering or mixed‐acid systems—such as H_2_SO_4_ combined with phosphate additives—can stabilize Ir─O bonding and reduce metal dissolution by moderating the local electric field [248, 513]. The introduction of CO_2_ further modifies the electrochemical interface, as (bi)carbonate formation alters the proton activity. These competing effects make the electrolyte composition a decisive factor in defining catalytic activity and degradation behavior.

Operando spectroscopy has revealed that pH‐dependent oxidation dynamics govern the intrinsic activity of transition‐metal‐based catalysts. A recent study on Co‐based oxides demonstrates that as the electrolyte becomes more acidic, the Co^2^^+^ → Co^3^^+^ oxidation potential shifts anodically, delaying the formation of Co^3^^+^–O active sites and resulting in higher OER overpotentials [319]. This finding establishes that interfacial Co oxidation controls the pH dependence of OER performance, explaining the lower activity and stability of Co‐based oxides in acidic media relative to alkaline media.

In acidic‐media CO_2_ electrolyzers, the electrolyte composition also determines ion transport, local pH gradients, and flooding risk [511]. Strongly acidic electrolytes facilitate CO_2_ regeneration (by promoting reaction between protons and locally generated (bi)carbonate ions), but the introduction of alkali cations renders the system vulnerable to salt precipitation within GDEs, which deteriorates their hydrophobicity and induces flooding [511]. Furthermore, ion migration across the PEM gradually neutralizes catholyte, thereby shifting both electrode microenvironments from their initial states [511]. Such electrolyte‐driven pH drifts strongly affect dissolution kinetics, and hence the stability of OER catalyst. Consequently, acidic‐media OER and CO_2_R are inherently coupled through electrolyte transport, and strategies that optimize electrolyte composition—such as cation‐free acidic‐media systems or selective ion‐blocking membranes—are crucial for maintaining balanced operation [511].

### 11.3 Temperature Effects

Temperature exerts a dual effect on OER performance by enhancing reaction kinetics while accelerating degradation [109]. Increasing temperature decreases activation barriers, improves proton conductivity, and reduces charge‐transfer resistance, thus boosting catalytic activity [514, 515]. However, elevated temperatures (>80°C) could exacerbate the dissolution of Ir and Ru oxides, increase Ti oxidation in PTLs, and accelerate membrane dehydration [516, 517, 518]. These effects might collectively raise the interfacial contact resistance and facilitate delamination at the catalyst–ionomer interface.

Importantly, temperature changes can fundamentally alter the reaction pathway. Operando differential electrochemical mass spectrometry on RhRu_3_O_x_ catalysts uncovered a temperature‐dependent mechanism evolution. At ambient‐level temperatures, the reaction proceeds via the AEM pathway. However, at temperatures greater than 60°C, the OER transitions to the LOM pathway, which greatly accelerates structural degradation [519]. DFT calculations indicate that this shift originates from the lowered oxygen‐vacancy formation energy at high temperatures, promoting lattice participation and irreversible metal dissolution.

In coupled acidic‐media CO_2_ electrolyzers, similar temperature‐dependent effects influence both OER and CO_2_R [511]. Elevated temperature improves ionic conductivity and lowers ohmic losses, but typically at the expense of intensified water crossover and gas flooding, indirectly affecting anode's local temperature and stability [511]. These findings confirm that temperature is not merely a kinetic enhancer but a mechanistic driver that links thermal energy input, reaction environment, and durability.

To mitigate such effects, uniform thermal management through flow‐field design, stack‐level heat management, and the use of high‐thermal‐conductivity PTLs are essential. Practicing these measures will be key for effective heat management and prolonged operational stability.

### 11.4 Flow, Gas Dynamics, and Compression

Flow‐field configuration, bubble dynamics, and mechanical compression jointly determine gas evacuation, water management, and interfacial contact at the anode during acidic‐media OER. Proper compression enhances electrical and thermal contact, reduces interfacial resistance, and ensures uniform pressure distribution between the PTL and CL. Moderate compression (i.e., 10–20%) typically yields a good balance between contact quality and gas permeability, whereas excessive pressure deforms the PTL microstructure, restricts oxygen channels, and increases mass‐transport resistance. On the other hand, over‐compression could cause oxygen accumulation, uneven water distribution, and mechanical cracking of the CL, PEM, and PTL, all of which deteriorate stability and lifetime.

Simultaneously, flow dynamics govern the mass transport of reactants and removal of bubbles, impacting both cathodic and anodic processes in acidic‐media CO_2_ electrolyzers [511]. At high current densities, accelerated OER coincides with CO_2_R, likely causing significant two‐phase flow through the electrodes. Recent operando studies show that salt precipitation and gas accumulation within the GDE alter electrolyte flow paths and increase pressure drop across the membrane, influencing both the CO_2_R selectivity and OER kinetics [511]. This interplay highlights the need for well‐optimized flow rate, compression, and porosity across both electrodes [511].

Therefore, an integrated approach that considers flow–compression coupling is required. Additively manufactured PTLs with hierarchical pore networks and tailored elasticity, combined with optimized flow‐field geometries, can sustain uniform gas transport and pressure balance, improving performance and durability [484].

### 11.5 Coupled Effects and Outlook

Operating parameters are inherently interconnected, with each factor influencing the others through electrochemical, mechanical, and thermal coupling. Elevated current densities increase bubble coverage and thermal stress; higher temperatures accelerate corrosion and delamination; and changes in electrolyte composition affect charge transfer and mass transport. Compression modifies porosity and conductivity, which in turn alter gas flow and local heating [520]. In acidic‐media CO_2_ electrolyzers, these parameters additionally govern ion migration, flooding behavior, CO_2_ regeneration, and CO_2_R efficiency, which directly affect durability and energy efficiency [511]. Consequently, optimizing one parameter in isolation is insufficient—a multidimensional control framework is required.

Recent advances in adaptive operating protocols provide promising directions. Dynamic voltage modulation mitigates transient over‐oxidation; temperature and flow regulation sustain mechanical integrity; and real‐time sensing of current and pressure distributions enables active feedback control. Integrating these adaptive strategies with advanced electrode architectures (Section 10) could yield self‐regulating acidic‐media CO_2_ electrolyzers capable of maintaining optimal operation under fluctuating loads.

Future efforts should aim to unify operando diagnostic insights, mechanistic modeling, and system engineering to design devices that harmonize efficiency, stability, and scalability for practical deployment.

## 12 Concluding Remarks

Acidic‐media CO_2_R represents a promising pathway toward carbon‐efficient chemical and fuel synthesis, yet its practical realization is fundamentally constrained by the anodic OER. This review has shown that acidic‐media OER is not merely a counter reaction, but system‐level phenomena that govern energy efficiency, durability, scalability, and resource sustainability in acidic‐media CO_2_ electrolyzers. Bridging atomic‐scale catalyst design, mesoscale electrode architecture, and macroscale operating conditions is therefore essential to translate mechanistic understanding into deployable acidic‐media CO_2_R technologies.

A central conclusion emerging from this review is that catalyst stability in acidic‐media OER is inherently dependent on the operating current‐density regime: stability trends derived from conventional low‐current half‐cell tests (e.g., ∼10 mA cm^−^^2^) are indispensable for intrinsic screening, but insufficient to predict durability under device‐relevant, high‐current operation (≥200 mA cm^−^^2^). This distinction underscores the need to evaluate OER catalysts under conditions that reflect practical electrolyzer operation when assessing their long‐term viability for acidic‐media CO_2_R systems.

**FIGURE 11:**
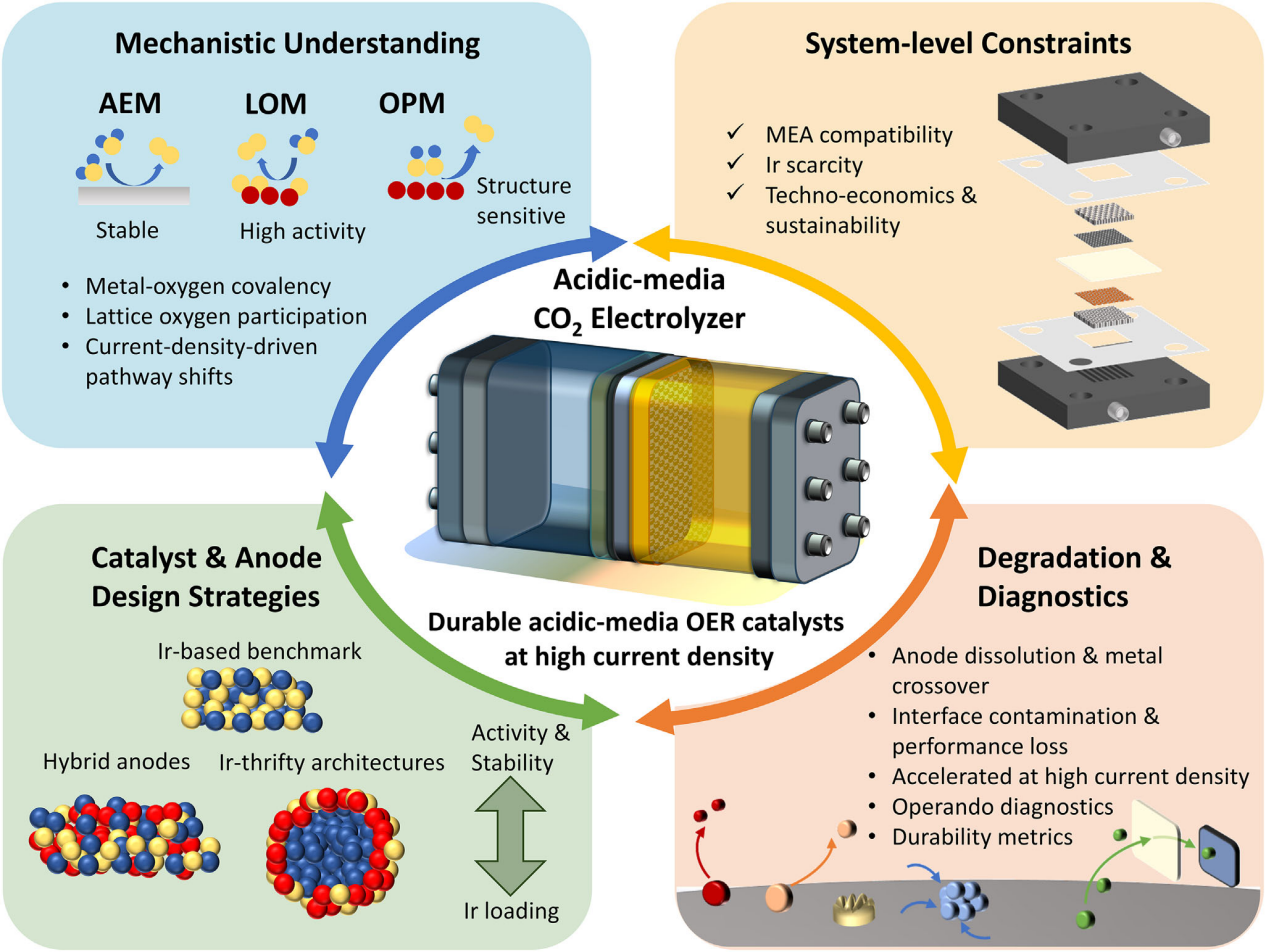
A schematic roadmap to industrial‐level deployment of acidic‐media CO_2_ electrolyzers.

Figure 11 provides a unified overview of the cross‐scale challenges and design priorities identified throughout this review. More specifically, it presents a schematic roadmap integrating mechanistic insights, catalyst design strategies, degradation phenomena, and system‐level constraints governing acidic‐media CO_2_ electrolyzers.

### 12.1 Priority Pathways and Quantitative Benchmarks for Acidic‐Media OER Electrodes

Based on the collective evidence reviewed herein, the development of acidic‐media OER electrodes for CO_2_ electrolyzers should be guided by a limited set of clearly defined, system‐integrated quantitative benchmarks, rather than by incremental activity optimization under idealized half‐cell conditions. In acidic‐media CO_2_ electrolyzers, anodic overpotential, proton flux, and catalyst stability cannot be meaningfully decoupled from cathodic CO_2_R behavior; instead, they manifest directly at the full‐cell level through operating voltage, energy efficiency, and long‐term durability. Consequently, roadmap benchmarks for OER electrode development must be formulated in a manner that reflects device‐relevant operating regimes and system‐level constraints.

First and foremost, durability under device‐relevant conditions represents the primary bottleneck. Practical acidic‐media CO_2_ electrolyzers require OER electrodes capable of sustained operation at current densities of at least ≥1 A cm^−^^2^ for ≥1000 h, with minimal voltage drift and strongly suppressed metal dissolution [376, 521, 522]. Stability trends derived from conventional low‐current half‐cell tests (e.g., ∼10 mA cm^−^^2^), while indispensable for intrinsic screening and mechanistic comparison, are insufficient to predict catalyst lifetime under realistic electrolyzer operation. High‐current durability therefore constitutes a defining benchmark for acidic‐media OER electrodes in CO_2_ electrolyzers.

Second, Ir utilization efficiency must be drastically improved to enable scalable deployment. To support MW‐scale systems within realistic resource constraints, target Ir loadings should be reduced toward <0.1 mg_Ir_ cm^−^^2^, corresponding to <0.1 g_Ir_ kW^−^^1^, without compromising activity or durability [63, 68, 522, 523]. Achieving this benchmark will likely rely on hybrid anode architectures that decouple catalytic function from structural support, including Ir‐based SACs, core–shell and thin‐film designs, and conductive or corrosion‐resistant scaffolds that enhance utilization efficiency while mitigating dissolution.

Third, anodic performance benchmarks must emphasize low overpotentials under practical operating conditions. To ensure competitive full‐cell energy efficiency and avoid excessive electricity consumption, acidic‐media OER electrodes should target anodic overpotentials below ∼300–350 mV at current densities of ≥1 A cm^−^^2^ [258, 524]. Because the anodic overpotential constitutes a dominant fraction of the full‐cell voltage in acidic‐media CO_2_ electrolyzers, meeting this benchmark is critical not only for energy efficiency but also for limiting thermodynamic driving forces that accelerate catalyst degradation.

Finally, system compatibility must be treated as a co‐equal design constraint alongside activity and stability. Acidic‐media OER electrodes must operate robustly within PEM‐ or BPM‐based membrane‐electrode assemblies, exhibit minimal metal‐ion crossover, and maintain interfacial integrity with membranes, PTLs, and CLs under prolonged operation. Collectively, these benchmarks define a set of actionable, quantitative milestones that transform the development of acidic‐media OER electrodes from a qualitative materials challenge into an engineering‐guided pathway aligned with the practical requirements of scalable acidic‐media CO_2_R technologies.

### 12.2 Methodological Consensus and Enabling Tools

Meeting the quantitative benchmarks defined in Section 12.1 requires a corresponding methodological consensus on how acidic‐media OER electrodes are evaluated under device‐relevant conditions. Despite substantial progress in catalyst development, inter‐laboratory variability and limited reproducibility remain persistent challenges, arising from differences in cell configuration, catalyst loading, electrolyte composition, operating protocols, and test duration. Such variations can significantly alter local reaction environments, transport limitations, and degradation pathways, leading to inconsistent assessments of activity, stability, and apparent durability across studies. As a result, absolute performance metrics reported in the literature must be interpreted with caution unless experimental conditions and evaluation protocols are explicitly aligned.

A central methodological distinction that emerges from acidic‐media OER studies is the difference between low‐current‐density screening and high‐current‐density durability assessment. Stability trends derived from conventional half‐cell tests at low current densities (e.g., ∼10 mA cm^−^^2^) remain indispensable for probing intrinsic electrochemical robustness, elucidating reaction mechanisms, and enabling meaningful cross‐study comparison. However, such conditions suppress many of the coupled transport, interfacial, and mechanical stresses that dominate catalyst degradation under practical electrolyzer operation. Consequently, evaluation protocols must explicitly separate low‐current intrinsic screening from durability assessments conducted at device‐relevant current densities (≥200 mA cm^−^^2^, and ultimately ≥1 A cm^−^^2^), in order to avoid overestimating catalyst lifetime and misinterpreting stability trends.

To enhance practical relevance, standardized durability metrics should be adopted alongside conventional activity descriptors. In addition to reporting overpotential and Tafel slopes, dissolution‐sensitive parameters, such as the S‐number, mass‐loss ratios measured by ICP‐MS, and voltage drift rates during sustained galvanostatic operation, should be systematically included. These metrics provide a direct link between catalyst degradation mechanisms—particularly metal dissolution and lattice‐oxygen redox—and long‐term anode performance under acidic conditions. ASTs should further be designed to emulate realistic operating environments, incorporating dynamic potential cycling, intermittent current loads, temperature gradients, and gas crossover effects, rather than relying solely on static potential holds.

Advanced operando characterization techniques constitute critical enabling tools for establishing mechanistic–durability correlations. Techniques, such as operando XAS, Raman spectroscopy, online ICP‐MS, and EQCM measurements, enable real‐time tracking of oxidation states, surface reconstruction, and metal dissolution during OER operation. When combined with post‐mortem structural and compositional analyses, these approaches provide a comprehensive picture of degradation pathways that cannot be captured by electrochemical data alone. Importantly, such diagnostics should be deployed not to increase experimental complexity, but to directly inform stability‐oriented design rules aligned with the quantitative benchmarks defined in Section 12.1.

Finally, theoretical frameworks play an increasingly important role in supporting methodological convergence. Constant‐potential DFT, grand‐canonical approaches, and AIMD simulations enable more realistic modeling of electrochemical interfaces, dissolution phenomena, and potential‐dependent surface reconstruction. When integrated with operando experimental data, these tools offer a pathway to bridge atomic‐scale insights with device‐level durability metrics. Establishing harmonized testing protocols and machine‐readable datasets that integrate electrochemical, structural, and durability descriptors will be essential for improving cross‐laboratory comparability and accelerating convergence toward practically deployable acidic‐media OER electrodes for CO_2_ electrolyzers.

### 12.3 Scalability, Techno‐Economics, and Sustainability Constraints

Beyond catalyst‐level performance, the quantitative benchmarks outlined in Section 12.1 are ultimately enforced by techno‐economic and resource constraints that emerge at scale. In acidic‐media CO_2_ electrolyzers, the OER electrode is not an isolated functional component, but a dominant contributor to full‐cell voltage, durability, capital cost, and materials sustainability. As a result, limitations associated with anodic overpotential, catalyst lifetime, and noble‐metal utilization directly translate into system‐level feasibility boundaries.

From a resource perspective, Ir scarcity constitutes a hard upper bound on global deployment. Recent geological and market surveys indicate that global primary Ir production in the past few years has remained on the order of ∼7–10 metric tons per year [525]. In parallel, recent scale‐up analyses of CO_2_R suggest that meaningful market penetration—even at partial substitution of major chemical and fuel markets—would require electrolyzer capacities in the hundreds‐of‐megawatts range [521]. When these projected deployment scales are contrasted with Ir‐based anode architectures derived from PEMWE, even gram‐level Ir utilization per kilowatt of installed capacity would translate into kilogram‐scale Ir demand per megawatt, rapidly approaching or exceeding current annual Ir supply if Ir utilization is not aggressively reduced. This order‐of‐magnitude mismatch underscores that Ir‐thrifty anode design is not an optional optimization, but a prerequisite for scalable acidic‐media CO_2_R.

Techno‐economic analyses further reveal that anodic overpotential, stack lifetime, and catalyst utilization jointly dominate the levelized cost of CO_2_‐derived products. Reducing anodic overpotentials directly lowers electricity consumption, which remains the largest operational expenditure in CO_2_R, while extending anode lifetime mitigates frequent stack replacement and associated capital costs. Consequently, performance improvements that appear incremental at the catalyst level can yield disproportionately large economic benefits when propagated across MW‐scale systems operating continuously. In this context, system‐level indicators—such as cell‐specific energy consumption (kWh kg^−^^1^ product), stack‐level capital expenditure ($ kW^−^^1^), Ir utilization intensity (g Ir kW^−^^1^), and, where applicable, the levelized cost of product (LCOX, $ kg^−^^1^)—provide the most meaningful framework for assessing the practical impact of OER electrode design strategies [521].

Sustainability considerations impose additional constraints that further reinforce these benchmarks. Long‐term deployment of acidic‐media CO_2_ electrolyzers will require materials circularity and supply‐chain resilience, particularly for platinum‐group metals and Ti‐based components. Closed‐loop recycling of Ir, Ru, and Ti, together with the use of recyclable membranes and PTLs, will be essential to reduce embodied carbon and alleviate resource supply risks. At the same time, minimizing metal dissolution and crossover is not only a durability requirement but also a sustainability imperative, as metal loss directly undermines recycling efficiency and increases environmental footprint.

Taken together, scalability, techno‐economics, and sustainability considerations converge on a consistent conclusion: the quantitative benchmarks defined for acidic‐media OER electrodes are not arbitrary targets, but system‐enforced constraints dictated by resource availability, economic viability, and long‐term deployment requirements. Addressing these constraints requires a coordinated approach that integrates catalyst design, electrode architecture, operating strategy, and recycling pathways, ensuring that advances in acidic‐media OER translate into deployable and sustainable acidic‐media CO_2_R technologies at scale.

## Author Contributions

**Mingcheng Huang**: investigation, methodology, visualization, formal analysis, writing – original draft. **Adnan Ozden**: writing – original draft, writing – review & editing, investigation, methodology, investigation, conceptualization, visualization, supervision, resources, project administration, funding acquisition.

## Conflicts of Interest

The authors declare no conflicts of interest.

## Data Availability

No data was used for the research described in the article.
